# Bioprospecting Sponge-Associated Microbes for Antimicrobial Compounds

**DOI:** 10.3390/md14050087

**Published:** 2016-05-02

**Authors:** Anak Agung Gede Indraningrat, Hauke Smidt, Detmer Sipkema

**Affiliations:** 1 Laboratory of Microbiology, Wageningen University, Dreijenplein 10, Wageningen 6703 HB, The Netherlands; anak1.indraningrat@wur.nl (A.A.G.I.); hauke.smidt@wur.nl (H.S.); 2 Department of Biology, Faculty of Mathematics and Science Education, Institut Keguruan dan Ilmu Pendidikan Persatuan Guru Republik Indonesia (IKIP PGRI) Bali, Jl. Seroja Tonja, Denpasar 80238, Indonesia

**Keywords:** antimicrobial compounds, sponges, sponge-associated microbes

## Abstract

Sponges are the most prolific marine organisms with respect to their arsenal of bioactive compounds including antimicrobials. However, the majority of these substances are probably not produced by the sponge itself, but rather by bacteria or fungi that are associated with their host. This review for the first time provides a comprehensive overview of antimicrobial compounds that are known to be produced by sponge-associated microbes. We discuss the current state-of-the-art by grouping the bioactive compounds produced by sponge-associated microorganisms in four categories: antiviral, antibacterial, antifungal and antiprotozoal compounds. Based on *in vitro* activity tests, identified targets of potent antimicrobial substances derived from sponge-associated microbes include: human immunodeficiency virus 1 (HIV-1) (2-undecyl-4-quinolone, sorbicillactone A and chartarutine B); influenza A (H1N1) virus (truncateol M); nosocomial Gram positive bacteria (thiopeptide YM-266183, YM-266184, mayamycin and kocurin); *Escherichia coli* (sydonic acid), *Chlamydia trachomatis* (naphthacene glycoside SF2446A2); *Plasmodium* spp. (manzamine A and quinolone 1); *Leishmania donovani* (manzamine A and valinomycin); *Trypanosoma brucei* (valinomycin and staurosporine); *Candida albicans* and dermatophytic fungi (saadamycin, 5,7-dimethoxy-4-*p*-methoxylphenylcoumarin and YM-202204). Thirty-five bacterial and 12 fungal genera associated with sponges that produce antimicrobials were identified, with *Streptomyces*, *Pseudovibrio*, *Bacillus*, *Aspergillus* and *Penicillium* as the prominent producers of antimicrobial compounds. Furthemore culture-independent approaches to more comprehensively exploit the genetic richness of antimicrobial compound-producing pathways from sponge-associated bacteria are addressed.

## 1. Introduction

Antimicrobial resistance (AMR) is an emerging global threat, decreasing the possibilities for prevention and treatment of infectious diseases caused by viruses, bacteria, parasites and fungi [1,2]. A global surveillance report by the World Health Organization (WHO) [2] indicated an increase of morbidity and mortality of infectious diseases due to AMR, which could lead to a world-wide economic loss of up to 100 trillion US dollars (USD) in 2050 as the result of a 2%–3% reduction in the gross domestic product (GDP) [1]. A conservative estimation is that AMR now annually attributes to 700,000 deaths globally, with a potential leap to 10 million in 2050 [1]. AMR is a response of microorganisms against antimicrobial compounds, which can arise via several mechanisms such as chromosomal mutations [1], binding site modifications [2] or horizontal transfer of genes conferring resistance [3]. For several pathogenic bacteria such as *Staphylococcus aureus* [4], *Pseudomonas aeruginosa* [1,5], and *Mycobacterium tuberculosis* [6], the emergence of multi drug resistant (MDR) strains has been reported, which make infections with these strains increasingly difficult to treat with currently available antibiotics [3].

In the context of the arms race between humans and infectious agents, the discovery and development of new types of antimicrobial compounds with pronounced bioactivity and clinical significance are urgent [4,5]. The efforts to modify existing drugs are often not effective to overcome the mutation rate of pathogens and do not lead to the introduction of new classes of antimicrobial compounds [6]. The terrestrial environment has been the main focus of microbial-derived drug discovery since the first report on Penicillin in 1929 [7], followed by the booming of new classes of antibiotics in 1960s [8]. Although novel antimicrobials are still being discovered from the soil niche, e.g., turbomycin A and B [9] and teixobactin [10], there are issues with de-replication, which significantly reduces the discovery rate of new compounds from heavily screened environments [11].

In comparison with soils, the marine environment has been largely neglected for discovery of antibiotics until recently, mainly because of accessibility issues, but yet hold a huge biodiversity and potential novelty of antimicrobial compounds [12]. Of many marine organisms, sponges (phylum Porifera) are considered as the most prolific source of therapeutic compounds as these animals harbour a large variety of secondary metabolites, many of which are beneficial for human health purposes [13,14,15,16,17]. The “Supply Issue” is the main obstacle to exploit the biological activity of sponges’ metabolites since a large quantity of biomaterial is required for experimental purposes [13]. Interestingly, in recent years an increasing number of studies highlighted that many active substances from sponges are of bacterial origin due to similarity to chemical structures found in terrestrial microorganisms [13,14,15]. Furthermore, several studies have reported a wide diversity of antimicrobial activities from sponge-associated microbes, which make these microbial communities a valuable source for novel antimicrobials [14,16,17,18,19,20].

This review highlights the current knowledge of antimicrobial compounds produced by sponge-associated microbes. Our definition of “antimicrobial” is not limited to antibacterial agents, but also includes compounds active against viruses, fungi and infectious protozoa. For each of the four biological activities, a few substances are highlighted because of their high activity, along with the most complete overview to date of other known compounds with antimicrobial activity from sponge-associated microorganisms. To compare different bioactive compounds and crude extracts, inhibitory concentrations of substances reviewed have been as much as possible expressed in the same unit (µg/mL). Original articles use minimum inhibitory concentrations (MIC), half maximum inhibitory concentrations (IC_50_) and the concentration of a drug that give the half-maximal response (EC_50_). As they are not easily converted, we sticked to the original measures.

Moreover, we analyzed the distribution of bacterial and fungal genera associated with sponges that have been reported to produce antimicrobial compounds to identify the most prolific genera. In addition, the potential for application of metagenomics to complement culture-dependent antimicrobial screening strategies is also discussed.

## 2. Antiviral Compounds

New antiviral compounds are needed due to the increased occurrence of diseases caused by viral infections and because of antiviral escape strategies [21]. Marine organisms, and sponges in particular, have been shown to be a valuable source for antivirals. For example, the discovery of the nucleosides spongothymidine and spongouridine from the sponge *Tethya crypta* was the basis for the compound Ara-A (vidarabine) that is active against the herpes simplex virus [21,22,23,24].

**Figure 1:**
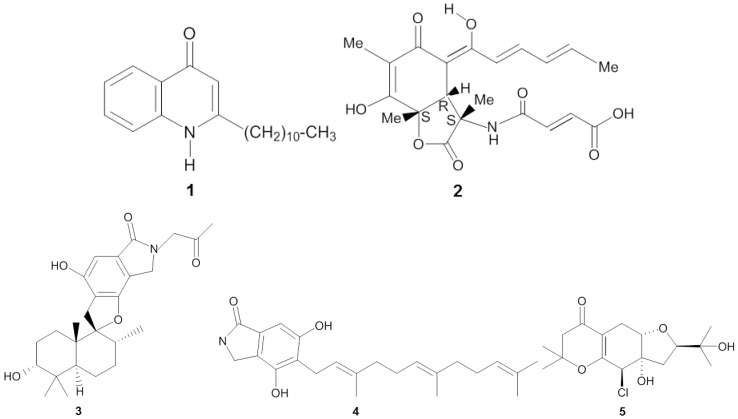
Chemical structures of the antiviral compounds 2-undecyl-4-quinolone (**1**), sorbicillactone A (**2**), stachybotrin D (**3**), chartarutine B (**4**), and truncateol M (**5**).

Screening of sponge-associated microbes yielded several prospective anti-HIV-1 (human immunodeficiency virus-1) compounds (Table 1 and Figure 1). Bultel-Poncé *et al.* [25] isolated *Pseudomonas* sp. 1531-E7 from the marine sponge *Homophymia* sp. resulting in the discovery of the antiviral compound 2-undecyl-4-quinolone (**1**) (Figure 1). The compound had an IC_50_ concentration as low as 10^−3^ µg/mL *in vitro* against HIV-1. Bringmann *et al.* [26] elucidated the chemical structure of sorbicillactone A (**2**) which was isolated from *Penicillium chrysogenum*, a fungus associated with the sponge *Ircinia fasciculata*. Sorbicillactone A displayed cytoprotective effects on HIV-1-infected cells of the human cell line H9 at concentrations of 0.1–1 µg/mL. In addition, *in vitro* testing using H9 cells indicated that sorbicillactone A reduced the appearance of the HIV-1 protein up to 70% at a concentration of 0.3 μg/mL [26]. The sponge-associated fungus *Stachybotrys chartarum* MXH-X73 produces the compound stachybotrin D (**3**), which exhibited anti-HIV-1 activity by targeting reverse transcriptase [27]. At EC_50_ concentrations from 2.73 µg/mL to 10.51 µg/mL, stachybotrin D was active not only against the wild type HIV-1 but also against several non-nucleoside reverse transcriptase inhibitor (NNRTI) resistant HIV-1 strains. Li *et al.* [28] reported identification of three other anti-HIV-1 compounds from *Stachybotrys chartarum*: chartarutine B, G, and H. Of these three chartarutine compounds, chartarutine B (**4**) showed the lowest concentration that resulted in 50% inhibition of HIV-1 (IC_50_ of 1.81 µg/mL), followed by chartarutine G (IC_50_ of 2.05 µg/mL) and chartarutine H (IC_50_ of 2.05 µg/mL), respectively.

Sponge-associated microbes have also been found to produce anti-influenza compounds (Table 1). Zhao *et al.* [29] elucidated 14 new isoprenylated cyclohexanols coined as truncateols A-N from the sponge-associated fungus *Truncatella angustata*, and these compounds were tested *in vitro* against the influenza A (H1N1) virus. Truncateols C, E and M displayed bioactivity against H1N1, with truncateol M (**5**) being the most potent inhibitor, as shown by its IC_50_ value of 2.91 µg/mL. This inhibitory concentration was almost six fold lower than that of the positive control oseltamivir at 14.52 µg/mL. Truncateol M was predicted to be active at the late stage of the virus infection, likely during the assembly or release step of the virion [29] due to resemblance of the inhibition patterns observed for neuraminidase-inhibitor drugs, e.g., zanamivir and oseltamivir [30]. In addition, the presence of a chlorine atom in the chemical structure of trucanteol M is of particular interest since halogenation often enhances bioactivity of a given compound [31,32].

## 3. Antibacterial Compounds

**Figure 2:**
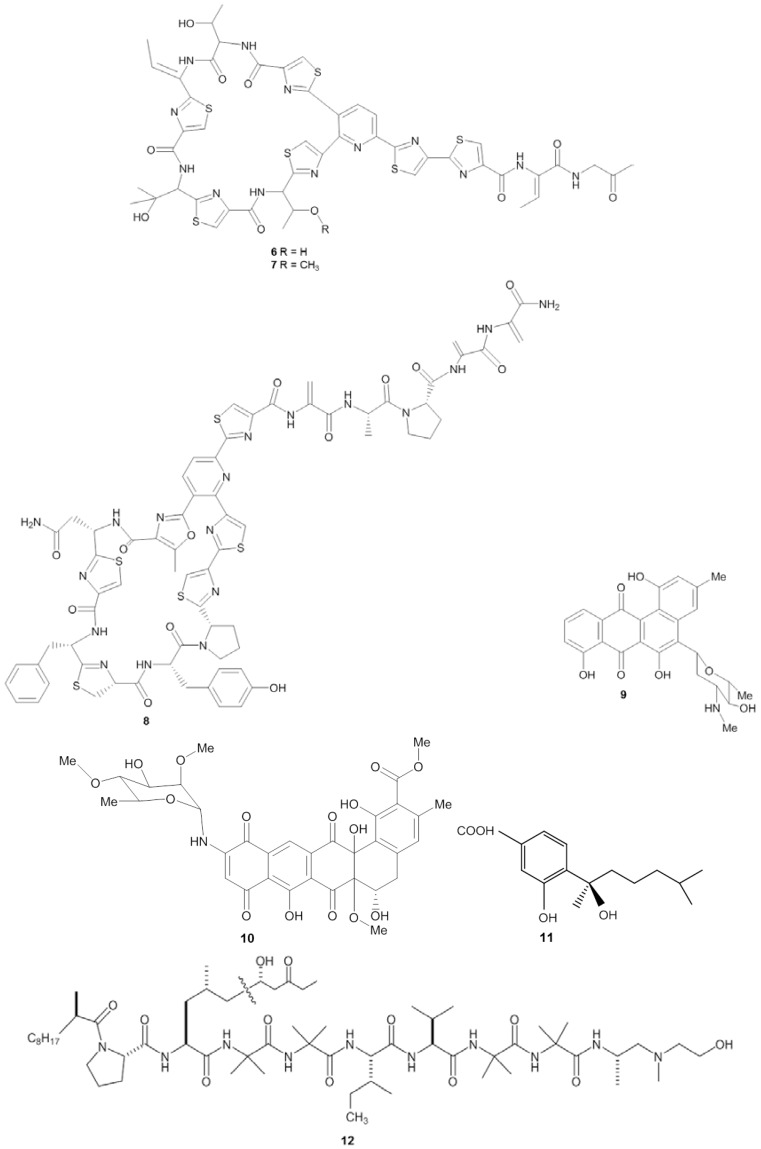
Chemical structures of the antibacterial compounds YM-266183 (**6**), YM-266184 (**7**), kocurin (**8**), mayamycin (**9**), naphthacene glycoside SF2446A2 (**10**), sydonic acid (**11**) and trichoderin A (**12**).

The screening procedure for antibacterial activity often includes both Gram positive and Gram negative target strains, including, e.g., *Staphylococcus* spp., *Streptococcus* spp., *Bacillus* spp., *Clostridium* spp., *Escherichia* spp., and *Pseudomonas* spp. From a medical point of view, these genera receive attention because they are well represented among the causative agents for human infectious diseases, such as pneumonia, urinary tract and blood stream infections [38,39]. Microbial isolates from marine sponges have been shown to exhibit bioactivity against a wide spectrum of pathogenic bacteria (Table 2). The novel thiopeptide antibiotics YM-266183 (**6**) and YM-266184 (**7**) (Figure 2), which were isolated from the sponge-associated bacterium *Bacillus cereus* QN03323, showed antibacterial activity against nosocomial infectious Gram positive bacteria *in vitro* [40,41]. Both YM-266183 and YM-266184 effectively inhibited *Staphylococcus aureus* and vancomycin-resistant *Enterococcus faecium* as indicated by minimal inhibition concentration (MIC) values as low as 0.025 µg/mL. In addition, compound YM-266184 was found particularly active against methicillin resistant *Staphylococcus aureus* (MRSA) with a MIC of 0.39 µg/mL. Compound YM-266183 also inhibited MRSA but required a two-fold higher concentration of the pure compound. Bioactivity of these thiopeptides was also observed against *Streptococcus epidermidis* and *Enterococcus* spp. (Table 2). The compound kocurin (**8**) was identified from three sponge-associated actinobacteria: *Kocuria marina* F-276,310, *Kocuria palustris* F-276,345, and *Micrococcus yunnanensis* F-256,446 [42,43]. Kocurin is a new member of the thiazolyl peptide family and exhibited anti-MRSA activity with an MIC of 0.25 µg/mL, which to date is the most potent anti-MRSA compound reported from sponge-associated microbes. Scheenemaan *et al.* [44] isolated *Streptomyces* sp. HB202 from the sponge *Haliclona simulans*, which lead to discovery of the polyketide mayamycin. *In vitro* assays with mayamycin (**9**) showed bioactivity against *S. aureus* and MRSA with IC_50_ values of 1.16 µg/mL and 0.58 µg/mL respectively, along with an IC_50_ of 0.14 µg/mL against *Staphylococcus epidermidis* [45].

Although many studies on antibacterial activity from sponge-associated microbes included Gram negative strains (Table 2), reports on pronounced antibacterial compounds active against Gram negative bacteria are limited in comparison to those that inhibit Gram positive strains. One of the examples of an inhibitor of a Gram negative bacterium is the compound naphthacene glycoside SF2446A2 (**10**) isolated from *Streptomyces* sp. RV15 that was originally obtained from the marine sponge *Dysidea tupha* [46]. Naphthacene glycoside SF2446A2 (**10**) inhibited the Gram-negative bacterium *Chlamydia trachomatis* at an IC_50_ value of 2.81 ± 0.24 µg/mL. Reimer *et al.* [46] underlined that compound **10** not only effectively inhibited the formation of chlamydial inclusion bodies during the primary infection but also affected the ability of *C. trachomatis* in producing viable progeny during the developmental cycle. *Chlamydia trachomatis* is an obligate intracellular Gram negative bacterium which is a leading cause of sexually transmitted diseases, and currently no methods are available to treat this infectious microorganism [46,47]. Li *et al.* [48] isolated four new bisabolane-typesesquiterpenoids: aspergiterpenoid A, (−)-sydonol, (−)-sydonic acid, (−)-5-(hydroxymethyl)-2-(2′,6′,6′-trimethyltetrahydro-2*H*-pyran-2-yl)phenol and a known compound (Z)-5-(Hydroxymethyl)-2-(6′-methylhept-2′-en-2′-yl)phenol from a sponge-associated *Aspergillus* sp. (Table 2). Of these five substances, the compound sydonic acid (**11**) exhibited the lowest MIC value against *Escherichia coli* at 1.33 µg/mL. This is the lowest inhibition concentration against *E.coli* reported from a compound produced by sponge-associated microbes although the inhibition concentration is still higher than the positive control ciprofloxacin (0.21 µg/mL) (Table 2).

Pruksakorn *et al.* [49] reported three prospective anti-tuberculosis compounds: trichoderin A (**12**), A1 and B from the sponge-associated fungus *Trichoderma* sp. 05FI48. Both under standard aerobic growth and dormancy-inducing hypoxic conditions, these three compounds inhibited *Mycobacterium smegmatis*, *M. bovis* BCG, and *M. tuberculosis* H37Rv with MIC values in the range of 0.02–2.0 µg/mL. Of these three compounds, trichoderin A was the most potent compound indicated by the lowest MIC values against those *Mycobacterium* strains. Additional analysis revealed that bioactivity of trichoderin A is based on its ability to inhibit adenosine triphosphate (ATP) synthesis of mycobacteria [50]. Compounds such as trichoderin A are particularly important because in many cases, pathogens such as *Campylobacter* spp., *Helicobacter pylori*, and *Legionella pneumophila* are difficult to treat due to the fact that they are present in a dormant state [51]. Such physiologically inactive cells highly contribute to the need for prolongued antibiotic treatments, which may lead to the emergence of resistant strains [52,53].

## 4. Antifungal Activity

The incidence rate of fungal infections has increased significantly over the past decades. This is mainly caused by clinical use of antibacterial drugs and immunosuppressive agents after organ transplantation, cancer chemotherapy, and advances in surgery [102,103]. Several fungal species that often cause human infections include *Candida albicans*, *Candida glabrata*, *Cryptococcus neoformans* and *Aspergillus fumigatus* [102,104,105]. The story becomes more complex as many of these pathogenic fungi develop resistance against available antifungal drugs, which will prolong duration of treatments [106].

**Figure 3:**
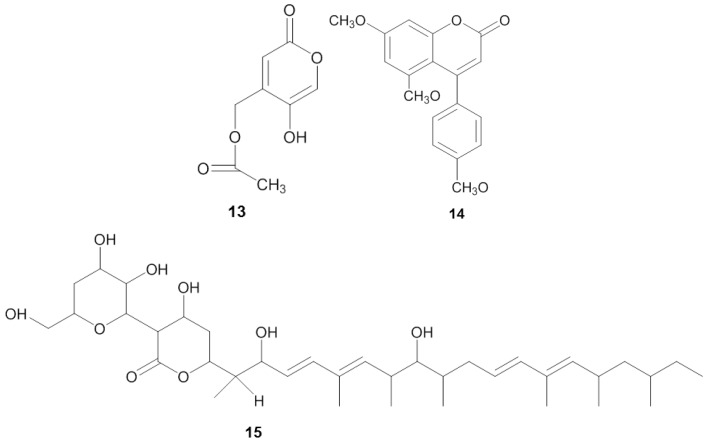
Chemical structures of the antifungal compounds saadamycin (**13**), 5,7-dimethoxy-4-*p*-methoxylphenylcoumarin (**14**) and YM-202204 (**15**).

Screening for antifungals is often focused on finding compounds active against *Candida albicans*, the prominent agent for candidiasis (Table 3). Invasive candidiasis is accounted as the most common nosocomial fungal infection resulting in an average mortality rate between 25%–38% [103]. El-Gendy *et al.* [107] isolated *Streptomyces* sp. Hedaya 48 from the sponge *Aplysina fistularis* and identified two compounds: the novel compound saadamycin (**13**) and the known compound 5,7-dimethoxy-4-*p*-methoxylphenylcoumarin (**14**) (Figure 3). Bioassays indicated that both saadamycin and 5,7-dimethoxy-4-*p*-methoxylphenylcoumarin displayed pronounced antifungal activity against *Candida albicans* with MIC values of 2.22 µg/mL and 15 µg/mL, respectively. In addition, both compounds displayed bioactivity against some pathogenic dermatophytes (skin-infecting fungi), such as *Epidermophyton floccosum*, *Trichophyton rubrum*, *Trichophyton mentagrophytes*, *Microsporum gypseum*, *Aspergillus niger*, *Aspergillus fumigatus*, *Fusarium oxysporum*, and *Cryptococcus humicolus* (Table 3). Further analysis showed that saadamycin displayed a more potent bioactivity indicated by a 3875 fold lower MIC than that of the reference compound, miconazole, whereas 5,7-dimethoxy-4-*p*-methoxylphenylcoumarin was around a 200 fold more potent than miconazole.

Antifungal activity was also detected from the sponge-associated fungus *Phoma* sp. Q60596. The sponge-derived fungus produced a new lactone compound, YM-202204 (15) [108], which was effective against *C. albicans* (IC_80_ of 6.25 µg/mL), along with *Cryptococcus neoformans* (IC_80_ of 1.56 µg/mL), *Saccharomyces cerevisiae* (IC_80_ of 1.56 µg/mL) and *Aspergillus fumigatus* (IC_80_ of 12.5 µg/mL). Furthermore, Nagai *et al.* [108] showed that YM-202204 was able to block the glycophosphatidylinositol (GPI) anchor, an important structure for protein attachment in the membrane of eukaryotic cells and one of the targets in developing antifungal drugs [109,110].

## 5. Antiprotozoal Activity

Malaria, caused by *Plasmodium* spp. infections, represents the most devastating protozoal disease worldwide, and results in both mortality and economic loss, mainly in developing countries [116]. Developing drugs with a better therapeutic profile against the parasite is one of the key aims of current malaria research, which includes screening for antimalarial substances from marine organisms [117,118]. 

**Figure 4:**
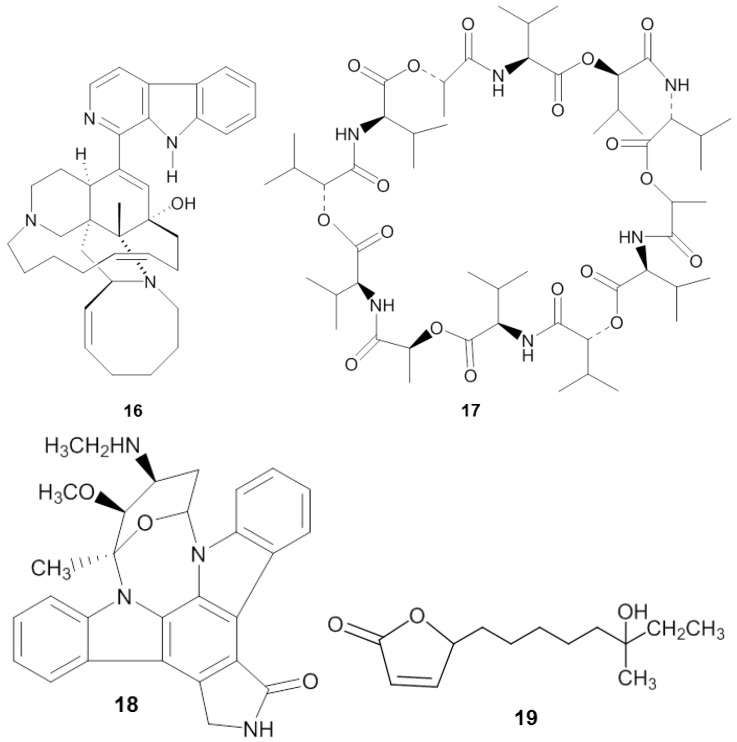
Chemical structures of the antiprotozoal compounds manzamine A (**16**), valinomycin (**17**), staurosporine (**18**) and butenolide (**19**).

Manzamine A (**16**) (Figure 4), first reported by Sakai and co-workers [119] from the sponge *Haliclona* sp., is a promising substance against *Plasmodium* spp. Initially, its antitumor property was of main interest, but subsequently diverse antimicrobial activities such as: anti-HIV, antibacterial, and antifungal were identified from the compound [120]. Currently the antimalaria properties of manzamine A are considered its most promising bioactivity. Manzamine A was shown to inhibit *P. falciparum* D6 and W3 clonal cell lines that are sensitive and resistant against the antimalarial chloroquine [121], with IC_50_ values of 0.0045 and 0.008 µg/mL, respectively [122]. Furthermore, *in vivo* screening by Ang *et al.* [116] showed that manzamine A at concentration of 0.008 µg/mL inhibited 90% growth of the parasite *Plasmodium berghei* that causes malaria in rodents. In addition, Rao *et al.* reported [122] that manzamine A displayed anti-*Leishmania* activity, indicated by IC_50_ and IC_90_ values of 0.9 µg/mL and 1.8 µg/mL, respectively, against *Leishmania donovani*.

Isolation of manzamine A from several other sponge species [120] raised the hypothesis that it was of microbial origin [123,124]. Hill *et al.* [125] confirmed this hypothesis by isolating *Micromonospora* sp. M42 as the microbial producer of manzamine A from the Indonesian sponge *Acanthostrongylophora* *ingens*. A series of analyses using molecular-microbial community analysis, and Matrix Assisted Laser Desorption Ionization-Mass Spectrometry (MALDI-MS) corroborated that indeed the strain *Micromonospora* sp. M42 synthesizes manzamine A [126,127]. Considering the therapeutic potential of manzamine A for treating malaria and leishmaniasis, *Micromonospora* sp. M42 could be a sustainable provider of the substance, because the “Sponge Supply Problem” has been overcome [127]. Moreover, identification of several manzamine-derivatives e.g. manzamine E, F, J, and 8-hydroxymanzamine A, from marine sponges which displayed antibacterial, antifungal and antiprotozoal activity [122,124], could also lead to isolation of associated microbial producers in the future.

Pimentel-Elardo *et al.* [128] identified three compounds with anti-Leishmania and anti-Trypanosoma activity from a sponge-associated *Streptomyces* sp, namely the cyclic depsipeptide valinomycin (**17**), the indolocarbazole alkaloid staurosporine (**18**) and butenolide (**19**) (Table 4). Valinomycin and staurosporine inhibited the growth of *L. major* with IC_50_ values of 0.12 µg/mL and 1.24 µg/mL, respectively. In addition, the three compounds displayed bioactivity against *Trypanosoma brucei* with IC_50_ values of 0.0036 µg/mL for valinomycin, 0.0051 µg/mL for staurosporine and 7.92 µg/mL for butenolide.

Scopel *et al.* [129] isolated two sponge-associated fungi, namely *Hypocrea lixii* F02 and *Penicillium citrinum* F40 (Table 4) that were active against the protozoal parasite *Trichomonas vaginalis*, which causes trichomoniasis, a sexually transmitted disease [130]. Culture filtrates of both isolates inhibited *T. vaginalis* ATCC 30236 and fresh clinical isolates, including the metronidazole-resistant TV-LACM2, with MIC values of 2.5 mg/mL. Further observation indicated that culture filtrates of these two fungi had no haemolytic effect against mammalian cells, which is one of the important criteria to further develop anti-protozoal drugs [129].

## 6. Dicussion

### 6.1. Antimicrobial Compounds from Sponge-Associated Microbes: What We Learned So Far

Bioprospecting is the effort to discover natural compounds with therapeutic and biological applications [140]. In line with this definition, sponge-associated microbes offer a huge potential as the source of antimicrobial substances as shown by many microbial isolates being reported to inhibit pathogenic reference strains *in vitro* and to synthesize active substances against one or several groups of infectious agents. Based on our review, antimicrobial compounds produced by sponge-associated microbes with the most pronounced bioactivity include: 2-undecyl-4-quinolone, sorbicillactone A, stachybotrin D and chartarutine B against HIV-1; truncateol M against H1N1 M; YM-266183, YM-266184, kocurin, mayamycin, sydonic acid, naphthacene glycoside SF2446A2 and trichoderin A against a variety of bacterial strains; saadamycin and YM-202204 against fungi; manzamine-A against malaria; and valinomycin against Trypanosoma. In this case the most pronounced activity is solely based on reported inhibition data and does not yet take potential side effects into account. Therefore the most promising compounds may be ones that have higher IC_50_ values, but cause less side effects. As these data are not available for the majority of the reported compounds, we have focused on the most potent compounds.

**Figure 5:**
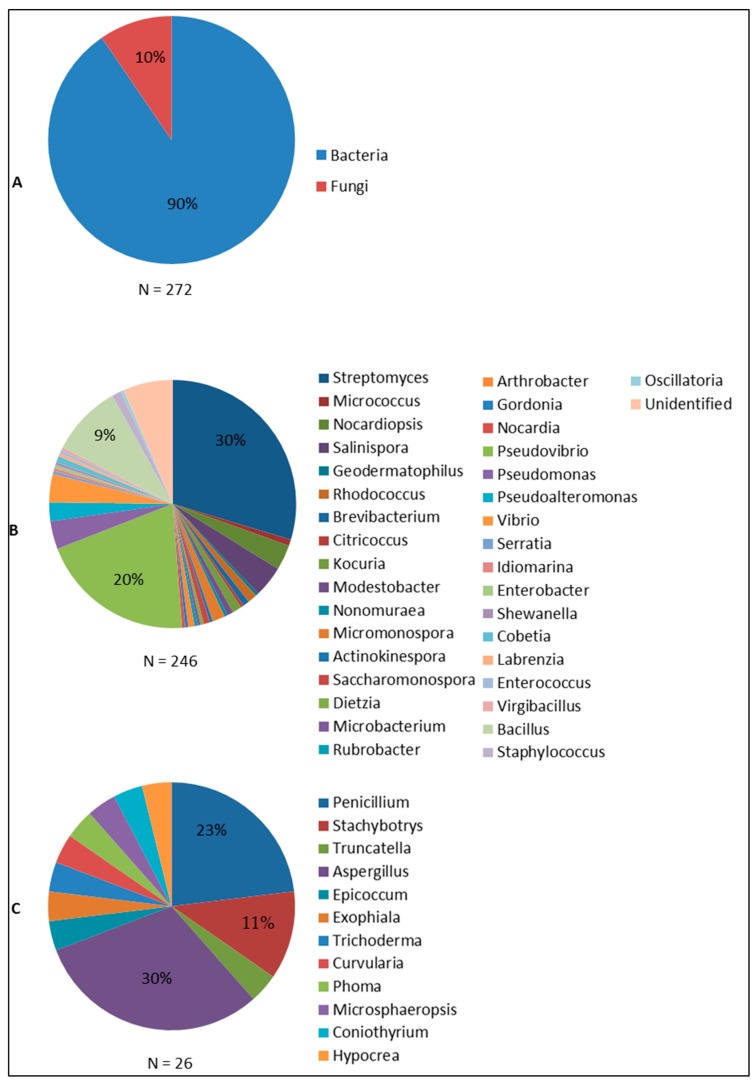
Distribution of sponge-associated microorganisms found to produce antimicrobial compounds: (**A**) Bacteria and Fungi; (**B**) Bacterial genera; and (**C**) Fungal genera. Figure 5 was made based on the summary of the taxonomic affiliations of sponge-associated microbes (*N* = 272) that were found to produce antimicrobials.

Sponge-associated bacteria and fungi are the two groups of microorganisms that have been found to produce antimicrobial compounds (Figure 5). The large majority of the antimicrobial compounds found in sponge-associated microbiota is produced by bacteria (90%), while fungi account for approximately 10% of the compounds reported. Sponge-associated bacteria derived antimicrobial compounds were found from 35 genera (Figure 5B). At a higher taxonomic level, these 35 bacterial genera can be classified into the four phyla Actinobacteria, Proteobacteria, Firmicutes and Cyanobacteria with percentages of 48.8%, 36.6%, 11.4% and 0.4% respectively. In contrast, sponge-associated fungi that have been found to produce antimicrobials are affiliated solely to the phylum Ascomycota.

*Streptomyces* is the most prominent genus as indicated by 30% of sponge bacteria-derived compounds. *Streptomyces* has become a main target for screening for bioactive compounds both from terrestrial and marine environments due to the high diversity of secondary metabolites they produce [141,142]. Of the many sponge-associated *Streptomyces* isolates reported, *Streptomyces* sp. HB202 and *Streptomyces* sp. RV15 are of particular interest in term of producing antibacterial compounds. *Streptomyces* sp. HB202, isolated from the sponge *Halichondria panicea* has been documented to produce three antibacterial substances: mayamycin, streptophenazine G and K, which are mainly active against Gram positive pathogenic bacteria (Table 2). *Streptomyces* sp. RV15, on the other hand, produces the compound naphthacene glycoside which up to now is the only anti-Chlamydia reported from sponge-associated microbes [46]. In addition, the report on crude extract inhibition of *Streptomyces* sp. RV15 against *S. aureus* and *E. faecalis* [82] may give a hint to discover other antibacterial substances from this strain. *Streptomyces* sp. Hedaya48 is currently the most potent sponge-associated bacterial isolate for antifungal activities with the production of saadamycin and 5,7-dimethoxy-4-*p*-methoxylphenylcoumarin [107]. In addition, isolation of the anti-Trypanosoma and anti-Leishmania compounds valinomycin, staurosporine and butenolide from *Streptomyces* sp. 43, 21 and 11 [128], affirms *Streptomyces* as the currently most prominent producer of antimicrobial substances from sponges.

*Pseudovibrio* follows as the second most prolific bacterial genus isolated from sponges (20%) with respect to antimicrobial activities. Reports on *Pseudovibrio* spp. are concentrated on antibacterial activity and are mainly based on screening of crude extracts. Up to now, tropodithietic acid is the only antibacterial compound that has been identified from *Pseudovibrio* [72]. Although representing a lower percentage of the sponge-associated bacteria found to produce antimicrobials than *Streptomyces* and *Pseudovibrio*, 9% of the currently known bioactives was found to be produced by sponge-associated *Bacillus* spp., with activities against viruses, bacteria and fungi. *Bacillus cereus* QNO3323 is currently the most prominent antimicrobial producer from this genus with the very potent thiopeptides YM-266183 and YM-266184 that are active against Gram positive bacteria.

Sponge-associated Ascomycota found to produce antimicrobials can be further classified into 12 genera. Of these 12 fungal genera, *Aspergillus* (30%) and *Penicillium* (23%) are currently the two most prominent groups of sponge-associated fungi reported as antimicrobial producers. This finding is not suprising since both *Aspergillus* and *Penicillium* are known prolific producers of secondary metabolites from other sources [143]. *Aspergillus versicolor* [58] and an unidentified *Aspergillus* sp. isolated from the sponge *Xestospongia testudinaria* [48] showed a strong antibacterial activity as indicated by potent inhibition of pathogenic bacteria. The antimicrobial activities found from sponge-associated *Penicillium* spp. are particular remarkable as it is the only fungal genus that is found to produce antivirals, antibacterials antifungals and antiprotozoals. *Penicillium chrysogenum* [26] and *Penicillium* sp. FF01 [57] are to date the most promising sponge-associated *Penicillium* isolates for which anti-HIV activity (sorbicillactone) and antibacterial activity (citrinin) were reported, respectively. Sponge-derived *Stachybotrys* spp. are only known for antiviral activity, particularly against HIV and enterovirus 71 (EV71), and there are no reports of other antimicrobial activities. Generally, although the number of produced antimicrobials is outnumbered by those of sponge-associated bacteria, sponge-associated fungi should be considered as an important reservoir of antimicrobial compounds.

When the chemical structures of sponge-microbe-derived compounds are considered, a rather diverse array of structures is observed, including peptides, terpenoids, phenazines, indoles, phenoles and polyketides. Sixty percent of the antivirals from sponge-associated microbes are ketone derivatives (quinolone, sorbicillactone, isoindolinone, butyrolactone, furanone, xanthone, methanone, phenone). Peptide derivatives constitute 19% of the total identified antibacterial substances and roughly 12.5% from the total antimicrobial compounds reviewed here. Phenazine derivatives are the second most frequently isolated class of antibacterial compounds from sponge-associated microbes (15%) as exemplified in this review by the antibacterial compounds streptophenazine [89], phenazine alkaloid antibiotics [55], 6-hydroxymethyl-1-phenazine-carboxamide and 1,6-phenazinedimethanol [94]. Phenazine is a nitrogen-containing heterocyclic compound with a wide range of biological activities [67,144], and several studies from terrestrial environments and chemically synthesized phenazines have been reported as antiviral [145], antibacterial [146], and antimalaria [147]. Moreover, this group of compounds is attractive for therapeutic application since their structures are relatively small and hence can easily reach tissues and organs [67,148].

### 6.2. Discovering Antimicrobial Compounds from Sponge-Associated Microbes: From Culture-Dependent to Culture-Independent Methods

Isolation of antimicrobial producers provides a valuable basis for assessing the biotechnological potential of sponge-associated microbes. In a wider perspective, however, only a small fraction of this sponge-microbial community has been isolated under laboratory conditions leaving the majority resistant to *in vitro* growth with current cultivation approaches [15,149,150]. Several studies have focused on improving cultivability of sponge-associated microbes. Some of the approaches include using low nutrient media [151], floating filter cultures [152], employing different carbon sources, e.g., lectin [153], sponge extracts [152], and *in situ* cultivation using a diffusion growth chamber [154]. Furthermore, flow-cytometry and density gradient centrifugation have been applied to separate sponge cells from their associated bacteria to enrich the inoculum [155,156]. Additionally, co-cultivation through mixing of two or more microbial isolates *in vitro* [157] is an approach proposed to discover more natural compounds from sponge-associated microbes. The idea behind co-culture lies in the fact that many biosynthetic gene clusters found in microorganisms remain cryptic under standard laboratory conditions, and co-cultivation might provide a possibility to activate these silent genes [158,159]. As an example, the co-culture by Dashti *et al.* [98] of the sponge-associated Actinobacteria, *Actinokinespora* sp. EG49 and *Nocardiopsis* sp. RV163, resulted in isolation of the antibacterial compound 1,6-dihydroxyphenazine, which was not found from the individual isolates. However, even if the cultivability of sponge-associated microbes is improved, there is a long way ahead to reach a point that we will be able to isolate and routinely cultivate 50% of the microbes that are found in sponges. At the same time, the advance of genetic and molecular studies has resulted in the development of tools to study genes, transcripts and proteins by directly analyzing environmental DNA, RNA and proteins, thus bypassing cultivation procedures [157]. In relation to screening for antimicrobial activity, metagenomics has been applied to identify antimicrobials of uncultivated microorganisms from terrestrial environments, such as the antimycobacterial nocardamine, the putative antibacterial activity of terragines A–E [160], violacein that is active against *S. aureus*, *Bacillus* sp. and *Streptococcus* sp. [161] and a polyketide with activity against the yeast *Saccharomyces cerevisiae* [162].

Two main metagenomic approaches, functional screening and sequence homology-based methods, are generally distinguished [163]. Functional screening relies on detection of the metabolic activities of metagenomic library clones without requiring any prior sequence information [163,164,165]. Gillespie *et al.* [9] applied function-based metagenomics with *E. coli* as expression host, to identify the antibiotics turbomycin A and B from a soil sample. MacNeil *et al.* [166] identified the antimicrobial indirubin by constructing a BAC (bacterial artificial chromosome) library in *E.coli*. Yung *et al.* [167] reported two hydrolytic enzymes from fosmid clones CcAb1 and CcAb2, which were derived from a metagenome of the sponge *Cymbastela concentrica* using *E. coli* as the host. Both fosmid clones inhibited the growth of *Bacillus* sp. with an inhibition diameter of 20 mm, and clone CcAb1 showed additional inhibition of *S. aureus* and an *Alteromonas* sp. with diameters of inhibition of 50 mm and 60 mm, respectively. Further phylogenetic analysis showed that active genes encoding for these enzymes were of microbial origin [167]. He *et al.* [168] constructed a fosmid library of the sponge *Discodermia calyx* using *E. coli* as the host and identified antimicrobial activity of the enzyme 3-hydroxypalmitic acid against *B. cereus* and *C. albicans*. In addition, using the same approach He *et al.* [169] observed an active clone, pDC113, that displayed a clear inhibition zone against *B. cereus*. Subsequently, 11 cyclodipeptides were identified from this clone. Generally, it can be stated that although a number of antimicrobials have been discovered through functional screening of metagenomic libraries from sponges, the expression of large gene clusters such as those encoding (polyketide synthase( PKS) and (non-ribosomal peptide synthetase (NRPS) is still a difficult hurdle to take. Several key elements need to be considered to achieve successful expression of biosynthetic gene clusters; namely mobilizing the biosynthetic pathway into a suitable vector, selecting an appropriate heterologous host and stably maintaining the gene clusters in the host [170]. The size of many of these gene clusters requires the use of cloning vectors that can accept large inserts, such as fosmids, or BACs if the required insert size is over 100 kb [171]. Selection of heterologous expression systems in particular is a crucial factor before applying functional metagenomics to identify antimicrobials, because expression hosts are microbes as well and especially clones that express genes encoding for enzymes involved in production of antimicrobials may therefore be non-viable. Ongley *et al.* [170] pointed out some considerations in selecting an expression host such as relatedness to the native producer, availability of genetic tools and precursors, a high growth rate, and suitability for fermentation at a large scale. *E. coli*, the most commonly used expression host, has limitations for expressing parts of metagenomes because, e.g., of the sheer size of some gene clusters, genes with deviating codon usage, incompatible regulatory elements, lack of biosynthesis precursors or unavailability of posttranslational modifications [165,172]. Therefore, in order to make screening for antimicrobials through metagenomic libraries more efficient, it is of utmost importance to diversify the suite of expression hosts used. Several non-*E.coli* hosts, such as *Agrobacterium tumefaciens*, *Bacillus subtilis*, *Burkholderia graminis*, *Caulobacter vibrioides*, *Pseudoalteromonas* *haloplanktis*, *Pseudomonas putida*, *Ralstonia metallidurans*, *Rhizobium leguminosarum*, *Streptomyces avermitilis*, *S. albus*, *Pseudomonas putida*, *Sulfolobus solfataricus*, *Thermus thermophilus*, *Thiocapsa roseopersicina* and *Saccharopolyspora* sp. have been developed and should be more seriously considered as expression hosts when performing metagenomic screenings for antimicrobials [165,172,173].

Sequence-based screening, on the other hand, requires information on the sequence of genes involved in the production of a natural product as guidance to search for similar sequences in a sequenced metagenomic library or scaffolds reconstructed from direct metagenomic sequencing [165]. Homology-based screening is suitable to identify a compound with highly conserved biosynthesis pathways, e.g., those mediated by PKS and NRPS [174]. Piel and colleagues [175,176,177,178,179] applied this method, and identified the antitumor polyketide onnamide from uncultivated bacteria of the sponge *T. swinhoei*. Sequence-based screening was applied by Fisch [180] to unravel the complete pathway of the polyketide psymberin that was found to possess a potent antitumor activity, from uncultivated sponge-associated microbes. By sequence-based screening of metagenomic libraries, Schirmer *et al.* [181] reported diverse polyketide gene clusters in microorganisms from the sponge *Discodermia dissoluta*. The development of techniques that yield longer read lengths, such as Pacific Biosciences (PacBio) RS II SMRT (Single Molecule Real-Time) sequencing technology, in which a single read can be extended over 10 kbp [182], can be instrumental in increasing the accuracy in assembling large gene clusters. Application of PacBio for secondary metabolite gene clusters has been reported by Alt and Wilkinson [183], who identified the 53,253 bp genomic fragment encoding the transacyltransferase (trans-AT) polyketide synthase (PKS) from a marine *Streptomyces* sp responsible for the production of the antibiotic anthracimycin (atc). Furthermore, using *Streptomyces coelicolor* as heterologous expression host, the authors confirmed production of anthracimycin [183]. Furthermore, single cell analysis by combining cell separation and fluorescence-assisted cell sorting (FACS) could be a strategy to overcome the complexity of the microbial community in sponges since this method can be used to select for genomes from microbes that are present in low abundance in the sponge leading to a simplified reconstruction of secondary metabolite gene clusters present in these bacteria [184]. This strategy has been applied by Wilson *et al.* [185] for resolving the gene clusters encoding the machinery needed for the production of the polytheonamides produced by the candidate genus *Entotheonella* from the sponge *Theonella swinhoei.*

**Figure 6:**
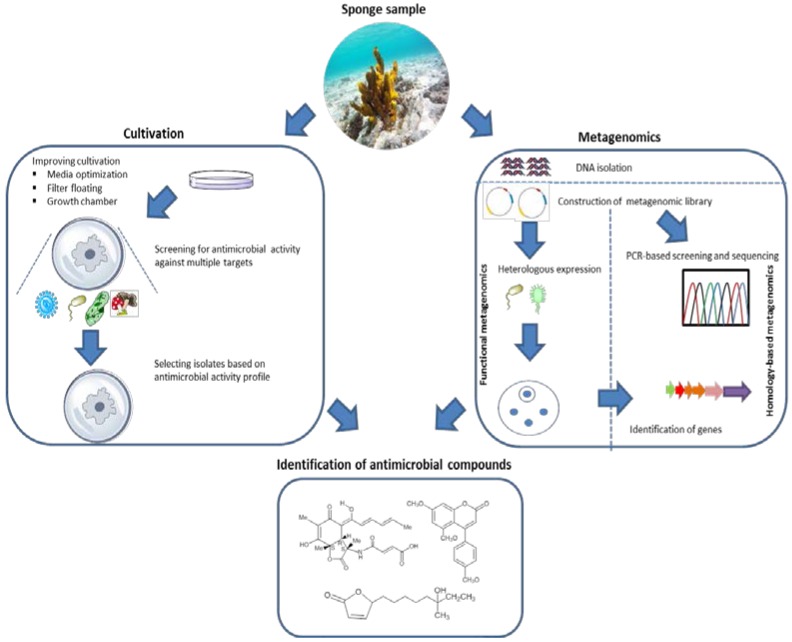
General overview of the strategies used to discover antimicrobial compounds from sponge-associated microorganisms.

Inspired by these examples, homology-based screening could be further exploited to identify biosynthesis gene sequences that could lead to the identification of novel antimicrobial substances from Nature’s excessive diversity. Moreover, application of homology-based screening can benefit from publicly available metagenomic sequencing data and prediction tools for analyzing biosynthesis gene clusters, e.g., AntiSMASH (Antibiotics and Secondary Metabolite Analysis Shell) [186,187]. Application of sequence-based screening, however, is limited by the fact that the found sequences need to be related to known compounds, inherently limiting the potential for novelty. Furthermore, information on gene sequences is no guarantee that the acquisition of a complete gene pathway has been obtained [188]. Therefore, sequence-based methagenomics should ideally be complemented by chemical analysis to confirm whether the predicted compound exists and is fully functional (Figure 6).

## 7. Conclusions and Outlook

Sponge-associated microbes already offer a rich source of potent antimicrobial compounds against viruses, bacteria, protozoa and fungi, and currently available compounds are predominantly active against HIV-1, H1N1, nosocomial Gram positive bacteria, *Escherichia coli*, *Plasmodium* spp, *Leishmania donovani*, *Trypanosoma brucei*, *Candida albicans* and dermatophytic fungi. *Streptomyces*, *Pseudovibrio*, *Bacillus*, *Aspergillus* and *Penicillium* are the microbial genera associated with sponges from which potent antimicrobial compounds are most frequently isolated. However, none of the antimicrobial compounds highlighted in this review have been succcesfully marketed as pharmaceuticals. To clearly translate bioactivity of these important compounds it is crucial to further unravel their mode of actions and measure their level of toxicity, since the majority of these studies has been focused on *in vitro* bioassays and elucidation of the chemical structures only.

The known versatility of antimicrobial activities found in sponge-associated microorganims could easily be expanded even without considering additional sponge sampling campaigns. Bioactivity screens of identified compounds or undefined sponge extracts is often restricted to a specific antimicrobial activity. The selection, for instance, relies on the specific research activities of the groups involved in isolating the microbes [117]. Consequently, it is probably safe to assume that other potent antimicrobial properties from many sponge isolates and their bioactive compounds remain undetected. Therefore, known antimicrobial compounds and producer strains are a valuable source for additional antimicrobial activities screenings using different target types (viruses, bacteria, fungi, protozoa and beyond). In addition, sponge-derived strain collections that comprise isolates that tested negative for antimicrobial activity at first may have done so, because the compound of interest is not produced under standard laboratory conditions. Exposure of these strains to potential microbial targets may lead to recovery of bioactivity that would otherwise go unnoticed.

Ideally, researchers who isolate microbes from sponges will deposit them to publicly available culture collections so that laboratories with complementary expertise and interests could benefit and screen the deposited isolates for different antimicrobial activities. This will make exchange of materials and knowledge that can be obtained much more efficient. Importantly, a fair agreement on intellectual property rights needs to be established for translating this into reality. Lastly, the revolutionary advance of next generation sequencing technologies combined with more diversified heterologous expression systems (Figure 6) are expected to open up the large unexplored reservoir of antimicrobials produced by yet uncultivated sponge-associated microbes.

## Figures and Tables

**Table 1:** Bioactive compounds with antiviral activity from sponge-associated microbes.

Sponge	Origin (Depth)	Microorganism	Phylum	Compound	Property	Target	Reference
*Homophymia* sp.	Touho, New Caledonia (ND)	*Pseudomonas* sp. 1531-E7	Proteobacteria	2-undecyl-4-quinolone	IC_50_ (10^−3^ µg/mL)	HIV-1	[25]
*Ircinia fasciculata*	Bight of Fetovaia, Italy (17.5 m)	*Penicillium chrysogenum*	Ascomycota	Sorbicillactone A	Reducing protein expression and activity of reverse transcriptase (0.3–1 µg/mL)	HIV-1	[26]
*Xestospongia testudinaria*	Paracel Islands (ND)	*Stachybotrys chartarum* MXH-X73	Ascomycota	Stachybotrin D	EC_50_ (3.71 µg/mL)	HIV-1	[27]
*Xestospongia testudinaria*	Paracel Islands (ND)	*Stachybotrys chartarum* MXH-X73	Ascomycota	Stachybotrin D	EC_50_ (3.09 µg/mL)	Non-nucleoside reverse transcriptase inhibitor (NNRTI) resistant HIV-1 strain _1RT-K103N_	[27]
*Xestospongia testudinaria*	Paracel Islands (ND)	*Stachybotrys chartarum* MXH-X73	Ascomycota	Stachybotrin D	EC_50_ (10.51 µg/mL)	NNRTI resistant HIV-_1RT-L100I, K103N_	[27]
*Xestospongia testudinaria*	Paracel Islands (ND)	*Stachybotrys chartarum* MXH-X73	Ascomycota	Stachybotrin D	EC_50_ (5.87 µg/mL)	NNRTI resistant HIV-_1RT-K103N, V108I_	[27]
*Xestospongia testudinaria*	Paracel Islands (ND)	*Stachybotrys chartarum* MXH-X73	Ascomycota	Stachybotrin D	EC_50_ (6.27 µg/mL)	NNRTI resistant HIV-_1RT-K103N, G190A_	[27]
*Xestospongia testudinaria*	Paracel Islands (ND)	*Stachybotrys chartarum* MXH-X73	Ascomycota	Stachybotrin D	EC_50_ (2.73 µg/mL)	NNRTI resistant HIV-_1RT-K103N, P225H_	[27]
*Niphates* sp.	Beibuwan Bay, China (10 m)	*Stachybotrys chartarum*	Ascomycota	Chartarutine B	IC_50_ (1.81 µg/mL)	HIV-1	[28]
*Niphates* sp.	Beibuwan Bay, China (10 m)	*Stachybotrys chartarum*	Ascomycota	Chartarutine G	IC_50_ (2.05 µg/mL)	HIV-1	[28]
*Niphates* sp.	Beibuwan Bay, China (10 m)	*Stachybotrys chartarum*	Ascomycota	Chartarutine H	IC_50_ (2.05 µg/mL)	HIV-1	[28]
*Amphimedon* sp.	Yongxin island, China (10 m)	*Truncatella angustata*	Ascomycota	Truncateol M	IC_50_ (2.91 µg/mL)	H1N1	[29]
*Callyspongia* sp.	Sanya, China (ND)	*Epicoccum* sp. JJY40	Ascomycota	Pyronepolyene C-glucoside iso-D8646-2-6	IC_50_ (56.06 µg/mL)	H1N1	[33]
*Callyspongia* sp.	Sanya, China (ND)	*Epicoccum* sp. JJY40	Ascomycota	Pyronepolyene C-glucoside, 8646-2-6	IC_50_ (62.07 µg/mL)	H1N1	[33]
Unidentified	Naozhou Sea, China (ND)	*Aspergillus terreus* MXH-23	Ascomycota	Butyrolactone III	Percentage of inhibition (53.9% ± 0.53% at 50 µg/L)	H1N1	[34]
Unidentified	Naozhou Sea, China (ND)	*Aspergillus terreus* MXH-23	Ascomycota	5-[(3,4-dihydro-2,2-dimethyl-2H-1-benzopyran-6-yl)-methyl]-3-hydroxy-4-(4-hydroxyphenyl)-2(5H)-furanone	Percentage of inhibition (57.8% ± 1.99% at 50 µg/L)	H1N1	[34]
Unidentified	Paracel Islands (ND)	*Aspergillus sydowii* ZSDS1-F6	Ascomycota	(*Z*)-5-(Hydroxymethyl)-2-(60)-methylhept-20-en-20-yl)-phenol	IC_50_ (14.30 µg/mL)	H3N2	[35]
Unidentified	Paracel Islands (ND)	*Aspergillus sydowii* ZSDS1-F6	Ascomycota	Diorcinol	IC_50_ (15.31 µg/mL)	H3N2	[35]
Unidentified	Paracel slands (ND)	*Aspergillus sydowii* ZSDS1-F6	Ascomycota	Cordyol C	IC_50_ (19.33 µg/mL)	H3N2	[35]
Unidentified	Paracel Islands (ND)	*Stachybotrys* sp. HH1 ZSDS1F1-2	Ascomycota	Stachybogrisephenone B	IC_50_ (10.2 µg/mL)	Enterovirus 71 (EV71)	[36]
Unidentified	Paracel Islands (ND)	*Stachybotrys* sp. HH1 ZSDS1F1-2	Ascomycota	Grisephenone A	IC_50_ (16.94 µg/mL	Enterovirus 71 (EV71)	[36]
Unidentified	Paracel Islands (ND)	*Stachybotrys* sp. HH1 ZSDS1F1-2	Ascomycota	3,6,8-Trihydroxy-1-methylxanthone	IC_50_ (10.4 µg/mL)	Enterovirus 71 (EV71)	[36]
*Petromica citrina*	Saco do Poço, Brazil (5–15 m)	*Bacillus* sp. B555	Firmicutes	Unidentified	IC_50_ (27.35 μg/mL) EC_50_ (>500 μg/mL)	Bovine viral diarrhea virus	[37]
*Petromica citrina*	Saco do Poço, Brazil (5–15 m)	*Bacillus* sp. B584	Firmcutes	Unidentified	IC_50_ (10.24 μg/mL) EC_50_ (277 μg/mL)	Bovine viral diarrhea virus	[37]
*Petromica citrina*	Saco do Poço, Brazil (5–15 m)	*Bacillus* sp. B616	Firmicutes	Unidentified	IC_50_ (47 μg/mL) EC_50_ (1500 μg/mL)	Bovine viral diarrhea virus	[37]

Table 1 is organised according to the target virusses. IC_50_: half maximum inhibitory concentration; EC_50_: the concentration of a drug that give the half-maximal response; ND: not determined; HIV: human immunodeficiency virus; H1N1 and H3N2 are influenza A virus subtypes.

**Table 2:** Bioactive compounds with antibacterial activity from sponge-associated microbes.

Sponge	Origin (Depth)	Microorganism	Phylum	Compound	Property	Target		References
*Halichondria japonica*	Iriomote island, Japan (ND)	*Bacillus cereus* QNO3323	Firmicutes	Thiopeptide YM-266183	MIC (0.025 µg/mL)	*Staphylococcus aureus*		[40,41]
*Halichondria japonica*	Iriomote island, Japan (ND)	*Bacillus cereus* QNO3323	Firmicutes	Thiopeptide YM-266184	MIC (0.025 µg/mL)	*S*. *aureus*		[40,41]
*Halichondria panicea*	Kiel Fjord, Baltic Sea, Germany (ND)	*Streptomyces* sp. HB202	Actinobacteria	Mayamycin	IC_50_ (1.16 µg/mL)	*S*. *aureus*		[45]
*Spheciospongia vagabunda*	Red Sea (ND)	*Micrococcus* sp. EG45	Actinobacteria	Microluside A	MIC (12.42 µg/mL)	*S. aureus* NCTC 8325		[54]
*Isodictya setifera*	Ross island, Antartica (30–40 m)	*Pseudomonas aeruginosa*	Proteobacteria	Phenazine-1-carboxylic acid	MIC (>4.99 µg/mL)	*S. aureus*		[55]
*Isodictya setifera*	Ross island, Antartica (30–40 m)	*Pseudomonas aeruginosa*	Proteobacteria	Phenazine-1-carboxamide	MIC (>4.99 µg/mL)	*S. aureus*		[55]
*Hymeniacidon perleve*	Bohai Sea, China (ND)	*Aspergillus versicolor* MF359	Ascomycota	5-Methoxydihydrosterigmatocystin	MIC (12.5 µg/mL)	*S. aureus*		[56]
*Melophus* sp.	Lau group, Fiji islands (10 m)	*Penicillium* sp. FF001	Ascomycota	Citrinin	MIC (1.95 µg/mL)	*S. aureus*		[57]
*Petrosia* sp.	Jeju island, Korea (20 m)	*Aspergillus versicolor*	Ascomycota	Averantin	MIC (3.13 µg/mL)	*S. aureus* SG511		[58]
*Petrosia* sp.	Jeju island, Korea (20 m)	*Aspergillus versicolor*	Ascomycota	Nidurufin	MIC (6.25 µg/mL)	*S. aureus* SG511		[58]
*Petrosia* sp.	Jeju island, Korea (20 m)	*Aspergillus versicolor*	Ascomycota	Averantin and nidurufin	MIC (3.13 µg/mL)	*S. aureus* 285		[58]
*Petrosia* sp.	Jeju island, Korea (20 m)	*Aspergillus versicolor*	Ascomycota	Averantin	MIC (1.56 µg/mL)	*S. aureus* 503		[58]
*Petrosia* sp.	Jeju island, Korea (20 m)	*Aspergillus versicolor*	Ascomycota	Nidurufin	MIC (3.13 µg/mL)	*S aureus* 503		[58]
*Hymeniacidon perleve*	Nanji island, China (ND)	*Pseudoalteromonas piscicida* NJ6-3-1	Ascomycota	Norharman (beta-carboline alkaloid)	MIC (50 µg/mL)	*S. aureus*		[59]
*Halichondria panicea*	Bogil island, Korea (ND)	*Exophiala* sp.	Ascomycota	Chlorohydroaspyrones A	MIC (62.5 µg/mL)	*S. aureus*		[60]
*Halichondria panicea*	Bogil island, Korea (ND)	*Exophiala* sp.	Ascomycota	Chlorohydroaspyrones B	MIC (62.5 µg/mL)	*S. aureus*		[60]
*Axinella* sp.	South China Sea, China (ND)	*Eupenicillium* sp.	Ascomycota	αβ-Dehydrocurvularin	MIC (375 µg/mL)	*S. aureus*		[61]
*Haliclona* sp.	Cagarras Archipelago, Brazil (4–20 m)	*Pseudomonas fluorescens* H40, H41 and *Pseudomonas aeruginosa* H51	Proteobacteria	Diketopiperazine	MIC (512 µg/mL)	*S. aureus*		[62]
*Spongia officinalis*	Southeast Coast India (10–15 m)	*Streptomyces* sp. MAPS15	Actinobacteria	2-pyrrolidone	MIC (500 µg/mL)	*S. aureus* PC6		[63]
*Dysidea herbacea*	Koror, Republic Palau (1 m)	*Oscillatoria spongeliae*	Cyanobacteria	2-(2′,4′-dibromophenyl)-4,6-dibromophenol	ND	*S. aureus*		[64]
*Hyrtios altum*	Aragusuku island, Japan (ND)	*Vibrio* sp.	Proteobacteria	Trisindoline	DOI (10 mm)	*S. aureus*		[65]
*Xestospongia testudinaria*	Bidong Island, Malaysia (ND)	*Serratia marcescens* IBRL USM 84	Proteobacteria	Prodigiosin	DOI (≤9 mm)	*S. aureus*		[66]
unidentified	South China Sea (10 m)	*Nocardiopsis* sp. 13-33-15 and 13-12-13	Actinobacteria	1,6-Dihydroxyphenazine	DOI (25 ± 0.6 mm)	*S. aureus* SJ51		[67]
				1,6-Dimethoxyphenazine	DOI (21 ± 0.1 mm)	*S. aureus* SJ51		
*Aplysina aerophoba*	Banyuls-sur-Mer, France (5–15 m)	*Bacillus subtilis* A184	Firmicutes	Surfactin Iturin Fengycin	ND	*S. aureus*		[68]
*Aplysina aerophoba*	Banyuls-sur-Mer, France (5–15 m)	*Bacillus subtilis* A190	Firmicutes	Surfactin	ND	*S. aureus*		[68]
*Halichondria* sp.	West Coast of India (10 m)	*Bacillus licheniformis* SAB1	Firmicutes	Indole	DOI (7–10 mm)	*S. aureus*		[69]
				3-Phenylpropionic	DOI (4–6 mm)	*S. aureus*		
*Niphates olemda*	Bali Bata National Park, Indonesia (ND)	*Curvularia lunata*	Ascomycota	1,3,8-Trihydroxy-6-methoxyanthraquinone (lunatin)	DOI (10 mm)	*S. aureus*		[70]
				Bisanthraquinone cytoskyrin A				
*Haliclona simulans*	Gurraig Sound Kilkieran Bay, Ireland (15 m)	*Bacillus subtilis* MMA7	Firmicutes	Subtilomycin	ND	*S. aureus*		[71]
*Polymastia boletiformis*, *Axinella dissimilis* and *Haliclona simulans*	Gurraig Sound, Kilkieran Bay, Ireland (15 m)	*Pseudovibrio* sp. W64, W69, W89, W74	Proteobacteria	Tropodithietic acid	DOI (≥2 mm)	*S. aureus*		[72]
*Polymastia boletiformis*, *Axinella dissimilis* and *Haliclona simulans*	Gurraig Sound, Kilkieran Bay, Ireland (15 m)	*Pseudovibrio* sp. JIC17, W10, W71, W74, W78, W96, WM33, WC15, WC30, HMMA3	Actinobacteria	Unidentified	DOI (≥1 mm)	*S. aureus*		[72]
*Polymastia boletiformis*, *Axinella dissimilis* and *Haliclona simulans*	Gurraig Sound, Kilkieran Bay, Ireland (15 m)	*Pseudovibrio* sp. JIC5, JIC6, W62, W63, W65, W99, WC43, W85, W94, WM31, WM34, WM40, WC13, WC21, WC22, WC32, WC41, HC6,	Proteobacteria	Unidentified	DOI (≥4 mm)	*S. aureus*		[72]
*Dendrilla nigra*	Vizhinjam coast, India (10–15 m)	*Streptomyces* sp. MSI051	Ascomycota	Unidentified	MIC (68 ± 2.8 µg protein/mL)	*S. aureus*		[73]
*Hymeniacidon perleve*	Nanji Island, China (ND)	*Pseudomonas* sp. NJ6-3-1	Proteobacteria	Unidentified	DOI (3–5 mm)	*S. aureus*		[74]
*Callyspongia* spp	Kovalam Coast, India (5–10 m)	*Aspergillus flavus* GU815344	Proteobacteria	Unidentified	DOI (27 mm)	*S. aureus*		[75]
*Haliclona* sp.	Cagarras Archipelago, Brazil (4–20 m)	*Pseudomonas fluorescens* H41	Proteobacteria	Unidentified	DOI (20 mm)	*S. aureus*		[76]
*Haliclona* sp.	Cagarras Archipelago, Brazil (4–20 m)	*Pseudomonas fluorescens* H40	Proteobacteria	Unidentified	DOI (23 mm)	*S. aureus*		[77]
*Haliclona* sp.	Cagarras Archipelago, Brazil (4–20 m)	*Pseudomonas fluorescens* H41	Firmicutes	Unidentified	DOI (20 mm)	*S. aureus*		[77]
*Haliclona* sp.	Cagarras Archipelago, Brazil (4–20 m)	*Pseudomonas aeruginosa* H51	Proteobacteria	Unidentified	DOI (30 mm)	*S. aureus*		[77]
*Dragmacidon reticulatus*	Cagarras Archipelago, Brazil (4–20 m)	*Bacillus pumilus* Dr31	Actinobacteria	Unidentified	DOI (19 mm)	*S. aureus*		[77]
*Petromica citrina*	Cagarras Archipelago, Brazil (4–20 m)	*Bacillus pumilus* Pc31	Firmicutes	Unidentified	DOI (40 mm)	*S. aureus*		[77]
*Petromica citrina*	Cagarras Archipelago, Brazil (4–20 m)	*Bacillus pumilus* Pc32	Firmicutes	Unidentified	DOI (40 mm)	*S. aureus*		[77]
*Clathrina aurea*	Cagarras Archipelago, Brazil (4–20 m)	*Pseudovibrio ascidiaceicola* Ca31	Proteobacteria	Unidentified	DOI (28 mm)	*S. aureus*		[77]
*Paraleucilla magna*	Cagarras Archipelago, Brazil (4–20 m)	*Pseudovibrio ascidiaceicola* Pm31	Proteobacteria	Unidentified	DOI (27 mm)	*S. aureus*		[77]
*Mycale microsigmatosa*	Cagarras Archipelago, Brazil (4–20 m)	*Pseudovibrio denitrificans* Mm37	Proteobacteria	Unidentified	DOI (20 mm)	*S. aureus*		[77]
*Axinella dissimilis*	Gurraig Sound, Kilkieran Bay, Ireland (15 m)	*Pseudovibrio* Ad30	Proteobacteria	Unidentified	ND	*S. aureus*		[78]
*Pseudoceratina clavata*	Heron Island, Great Barrier Reef (14 m)	*Salinispora* sp. M102, M403, M412, M413, M414, SW10, SW15 and SW17	Actinobacteria	Unidentified	DOI (>5 mm)	*S. aureus*		[79]
*Pseudoceratina clavata*	Heron Island, Australia (14 m)	*Salinispora* sp. SW02	Actinobacteria	Unidentified	DOI (<5 mm)	*S. aureus*		[79]
*Dendrilla nigra*	Southeast coast of India (ND)	*Streptomyces* sp. BTL7	Actinobacteria	Unidentified	DOI (16 mm)	*S. aureus*		[80]
*Mycale* sp.	Gulei Port, Fujian, China (ND)	*Bacillus* sp. HNS004, HNS010	Firmicutes	Unidentified	DOI (15–30 mm)	*S. aureus*		[81]
*Mycale* sp.	Gulei Port, Fujian, China (ND)	*Vibrio* sp. HNS022, HNS029	Proteobacteria	Unidentified	DOI (15–30 mm)	*S. aureus*		[81]
*Mycale* sp.	Gulei Port, Fujian, China (ND)	*Streptomyces* sp. HNS054	Actinobacteria	Unidentified	DOI (15–30 mm)	*S. aureus*		[81]
*Mycale* sp.	Gulei Port, Fujian, China (ND)	*Bacillus* sp. HNS005	Firmicutes	Unidentified	DOI (10–15 mm)	*S. aureus*		[81]
*Mycale* sp.	Gulei Port, Fujian, China (ND)	*Cobetia* sp. HNS027; *Streptomyces* sp. HNS047, HNS056; *Nocardiopsis* sp. HNS048, HNS051, HNS055; *Nocardia* sp. HNS052	Actinobacteria	Unidentified	DOI (10–15 mm)	*S. aureus*		[81]
*Mycale* sp.	Gulei Port, Fujian, China (ND)	*Bacillus* sp. HNS015	Firmicutes	Unidentified	DOI (8–10 mm)	*S. aureus*		[81]
*Mycale* sp.	Gulei Port, Fujian, China (ND)	*Pseudomonas* sp. HNS021	Proteobacteria	Unidentified	DOI (8–10 mm)	*S. aureus*		[81]
*Mycale* sp.	Gulei Port, Fujian, China (ND)	*Cobetia* sp. HNS023; *Vibrio* sp. HNS038; *Labrenzia* sp. HNS063;* Streptomyces* sp. HNS049;* Nocardiopsis* sp. HNS058	Actinobacteria	Unidentified	DOI (8–10 mm)	*S. aureus*		[81]
unidentified	Rovinj, Croatia (3–20 m)	*Streptomyces* sp. RV15	Actinobacteria	Unidentified	DOI (17 mm)	*S. aureus*		[82]
unidentified	Rovinj, Croatia (3–20 m)	*Dietzia* sp*.* EG67	Actinobacteria	Unidentified	DOI (13 mm)	*S. aureus*		[82]
unidentified	Rovinj, Croatia (3–20 m)	*Microbacterium* sp*.* EG69	Actinobacteria	Unidentified	DOI (13 mm)	*S. aureus*		[82]
unidentified	Rovinj, Croatia (3–20 m)	*Micromonospora* sp. RV115	Actinobacteria	Unidentified	DOI (12 mm)	*S. aureus*		[82]
unidentified	Rovinj, Croatia (3–20 m)	*Rhodococcus* sp. EG33	Actinobacteria	Unidentified	DOI (12 mm)	*S. aureus*		[82]
unidentified	Rovinj, Croatia (3–20 m)	*Rubrobacter* sp. RV113	Actinobacteria	Unidentified	DOI (9 mm)	*S. aureus*		[82]
*Suberites carnosus*	Lough Hyne, Co. Cork, Ireland (15 m)	*Arthrobacter* sp. W13C11	Actinobacteria	Unidentified	ND	*S. aureus*		[83]
*Suberites carnosus*	Lough Hyne, Co. Cork, Ireland (15 m)	*Pseudovibrio* sp. W13S4, W13S21, W13S23, W13S26, W13S31	Proteobacteria	Unidentified	ND	*S. aureus*		[83]
*Aplysina aerophoba* and *Aplysina cavernicola*	Marseille and Banyuls sur Mer, France (ND)	*Bacillus* SB8, SB17, *Enterococcus* SB91	Firmicutes	Unidentified	DOI (12–16 mm)	*S. aureus*		[84]
*Aplysina aerophoba* and *Aplysina cavernicola*	Marseille and Banyuls sur Mer, France (ND)	*Arthrobacter* SB95	Actinobacteria	Unidentified	DOI (12–16 mm)	*S. aureus*		[84]
*Aplysina aerophoba* and *Aplysina cavernicola*	Marseille and Banyuls sur Mer, France (ND)	unidentified low G + C Gram positive SB122 and SB144	Unidentified	Unidentified	DOI (12–16 mm)	*S. aureus*		[84]
*Aplysina aerophoba* and *Aplysina cavernicola*	Marseille and Banyuls sur Mer, France (ND)	α-Proteobacteria SB6, SB55, SB63, SB89, SB156, SB197, SB202, SB207, SB214	Proteobacteria	Unidentified	DOI (12–16 mm)	*S. aureus*		[84]
*Dysidea granulosa*	Kavaratti Island, India (ND)	*Enterobacter* sp. TTAG	Proteobacteria	Unidentified	DOI (22 mm)	*S. aureus*		[85]
*Petrosia ficiformis*	Paraggi, Ligurian Sea, Italy (8 m)	*Rhodococcus* sp. E1	Actinobacteria	Unidentified	ND	*S. aureus*		[86]
Unidentified	Atlantic coast, USA (ND)	*Kocuria palustris* F-276,310; *Kocuria marina* F-276,345 *Micrococcus yunnanensis* F-256,446	Actinobacteria	Kocurin	MIC (0.25 µg/mL)	methicillin-resistant *Staphylococcus aureus* (MRSA)		[42,43]
*Halichondria japonica*	Iriomote island, Japan (ND)	*Bacillus cereus* QNO3323	Firmicutes	Thiopeptide YM-266183	MIC (0.78 µg/mL)	MRSA		[40,41]
*Halichondria japonica*	Iriomote island, Japan (ND)	*Bacillus cereus* QNO3323	Firmicutes	Thiopeptide YM-266184	MIC (0.39 µg/mL)	MRSA		[40,41]
*Halichondria panicea*	Kiel Fjord, Baltic Sea, Germany (ND)	*Streptomyces* sp. HB202	Actinobacteria	Mayamycin	IC50 (0.58 µg/mL)	MRSA		[45]
*Melophus* sp.	Lau group, Fiji islands (10 m)	*Penicillium* sp. FF001	Ascomycota	Citrinin	MIC (3.90 µg/mL)	MRSA		[57]
*Halichondria panicea*	Bogil island, Korea (ND)	*Exophiala* sp.	Ascomycota	Chlorohydroaspyrones A	MIC (125 µg/mL)	MRSA		[60]
				Chlorohydroaspyrones B	MIC (62.5 µg/mL)	MRSA		
*Callyspongia* spp.	Gulf of Mannar, India (ND)	*Pseudomonas* spp. RHLB 12	Proteobacteria	Chromophore compound	DOI (4 mm) at 50 µM	MRSA		[87]
*Xestospongia testudinaria*	Bidong Island, Malaysia (ND)	*Serratia marcescens* IBRL USM 84	Proteobacteria	Prodigiosin	DOI (22.5 mm)	MRSA		[66]
*Halichondria* sp.	West Coast of India (10 m)	*Bacillus licheniformis* SAB1	Firmicutes	Indole 3-phenylpropionic	DOI (4–6 mm)	MRSA		[69]
*Haliclona simulans*	Gurraig Sound Kilkieran Bay, Ireland (15 m)	*Bacillus subtilis* MMA7	Firmicutes	Subtilomycin	ND	MRSA		[71]
*Haliclona* sp.	Cagarras Archipelago, Brazil (4–20 m)	*Pseudomonas fluorescens* H40	Proteobacteria	Unidentified	DOI (23 mm)	MRSA		[77]
*Haliclona* sp.	Cagarras Archipelago, Brazil (4–20 m)	*Pseudomonas fluorescens* H41	Proteobacteria	Unidentified	DOI (27 mm)	MRSA		[77]
*Haliclona* sp.	Cagarras Archipelago, Brazil (4–20 m)	*Pseudomonas aeruginosa* H51	Proteobacteria	Unidentified	DOI (17 mm)	MRSA		[77]
*Axinella dissimilis*	Gurraig Sound, Kilkieran Bay, Ireland (15 m)	*Pseudovibrio* Ad30	Proteobacteria	Unidentified	ND	MRSA		[78]
*Haliclona simulans*	Gurraig Sound, Kilkieran Bay, Ireland (15 m)	*Streptomyces* sp. SM2 and SM4	Actinobacteria	Unidentified	ND	MRSA		[88]
*Haliclona* sp.	Cagarras Archipelago, Brazil (4–20 m)	*Pseudomonas fluorescens* H40	Proteobacteria	Unidentified	DOI (20 mm)	community-associated MRSA		[77]
*Haliclona* sp.	Cagarras Archipelago, Brazil (4–20 m)	*Pseudomonas fluorescens* H41	Proteobacteria	Unidentified	DOI (22 mm)	community-associated MRSA		[77]
*Haliclona* sp.	Cagarras Archipelago, Brazil (4–20 m)	*Pseudomonas aeruginosa* H51	Proteobacteria	Unidentified	DOI (43 mm)	community-associated MRSA		[77]
*Petromica citrina*	Cagarras Archipelago, Brazil (4–20 m)	*Bacillus pumilus* Pc31	Firmicutes	Unidentified	DOI (40 mm)	community-associated MRSA		[77]
Petromica citrina	Cagarras Archipelago, Brazil (4–20 m)	*Bacillus pumilus* Pc32	Firmicutes	Unidentified	DOI (40 mm)	community-associated MRSA		[77]
Clathrina aurea	Cagarras Archipelago, Brazil (4–20 m)	*Pseudovibrio ascidiaceicola* Ca31	Proteobacteria	Unidentified	DOI (17 mm)	community-associated MRSA		[77]
*Paraleucilla magna*	Cagarras Archipelago, Brazil (4–20 m)	*Pseudovibrio ascidiaceicola* Pm31	Proteobacteria	Unidentified	DOI (25 mm)	community-associated MRSA		[77]
*Mycale microsigmatosa*	Cagarras Archipelago, Brazil (4–20 m)	*Pseudovibrio denitrificans* Mm37	Proteobacteria	Unidentified	DOI (20 mm)	community-associated MRSA		[77]
*Aplysina aerophoba*	Banyuls-sur-Mer, France (15 m)	*Bacillus subtilis* A202	Firmicutes	Iturin	ND	multi drug-resistant *S. aureus*		[68]
*Halichondria panicea*	Bogil island, Korea (ND)	*Exophiala* sp.	Ascomycota	Chlorohydroaspyrones A	MIC (125 µg/mL)	multi drug-resistant *S. aureus*		[60]
				Chlorohydroaspyrones B	MIC (125 µg/mL)	multi drug-resistant *S. aureus*		[60]
*Haliclona simulans*	Gurraig Sound Kilkieran Bay, Ireland (15 m)	*Bacillus subtilis* MMA7	Firmicutes	Subtilomycin	ND	heterogeneous vancomycin intermediate *Staphylococcus aureus* (hVISA)		[71]
*Axinella dissimilis*	Gurraig Sound, Kilkieran Bay, Ireland (15 m)	*Pseudovibrio* Ad30	Proteobacteria	Unidentified	ND	hVISA		[78]
*Haliclona simulans*	Gurraig Sound Kilkieran Bay, Ireland (15 m)	*Streptomyces* sp. SM2 and SM4	Proteobacteria	Unidentified	ND	hVISA		[88]
*Haliclona simulans*	Gurraig Sound Kilkieran Bay, Ireland (15 m)	*Streptomyces* sp. SM2 and SM4	Proteobacteria	Unidentified	ND	vancomycin intermediate *Staphylococcus aureus* (VISA)		[88]
*Melophus* sp.	Lau group, Fiji islands (10 m)	*Penicillium* sp. FF001	Ascomycota	Citrinin	MIC (0.97 µg/mL)	rifampicin-resistant *S.aureus*		[57]
*Halichondria panicea*	Baltic Sea (ND)	*Streptomyces* sp. HB202	Actinobacteria	Mayamycin	IC_50_ (0.14 µg/mL)	*Staphylococcus epidermidis*		[45]
*Halichondria panicea*	Kiel Fjord, Baltic Sea, Germany (ND)	*Streptomyces* sp. HB202	Actinobacteria	Streptophenazines G	IC_50_ (3.57 ± 0.21 µg/mL)	*S. epidermidis*		[89]
*Halichondria panicea*	Kiel Fjord, Baltic Sea, Germany (ND)	*Streptomyces* sp. HB202	Actinobacteria	Streptophenazines K	IC_50_ (6.16 ± 0.85 µg/mL)	*S. epidermidis*		[89]
*Axinella corrugata*	Arvoredo Biological Marine Reserve, Brazil (ND)	*Penicillium* sp.	Ascomycota	Dipeptide cis-cyclo(leucyl-tyrosyl)	reducing 85% of biofilm formation at 1000 µg/mL	*S. epidermidis*		[90]
unidentified sponge	Vizhijam coast (10–12 m)	*Aspergillus clavatus* MFD15	Ascomycota	1*H*-1,2,4-Triazole-3-carboxaldehyde 5-methyl	MIC (800 ± 10 µg/mL)	*S. epidermidis*		[91]
*Spongia officinalis*	Southeast Coast India (10–15 m)	*Streptomyces* sp. MAPS15	Actinobacteria	2-Pyrrolidone	MIC (500 µg/mL)	*S. epidermidis* PC5		[63]
*Xestospongia testudinaria*	Bidong Island, Malaysia (ND)	*Serratia marcescens* IBRL USM 84	Proteobacteria	Prodigiosin	DOI (<9 mm)	*S. epidermidis*		[66]
*Aplysina aerophoba*	Banyuls-sur-Mer, France (5–15 m)	*Bacillus subtilis* A184	Firmicutes	Surfactin Iturin Fengycin	ND	*S. epidermidis*		[68]
*Aplysina aerophoba*	Banyuls-sur-Mer, France (5–15 m)	*Bacillus subtilis* A190	Firmicutes	Surfactin	ND	*S. epidermidis*		[68]
*Haliclona* sp.	Cagarras Archipelago, Brazil (4–20 m)	*Pseudomonas fluorescens* H40	Proteobacteria	Unidentified	DOI (35 mm)	*S. epidermidis*		[77]
*Haliclona* sp.	Cagarras Archipelago, Brazil (4–20 m)	*Pseudomonas fluorescens* H41	Proteobacteria	Unidentified	DOI (30 mm)	*S. epidermidis*		[77]
*Haliclona* sp.	Cagarras Archipelago, Brazil (4–20 m)	*Pseudomonas aeruginosa* H51	Proteobacteria	Unidentified	DOI (28 mm)	*S. epidermidis*		[77]
*Dragmacidon* *reticulatus*	Cagarras Archipelago, Brazil (4–20 m)	*Bacillus pumilus* Dr31	Firmicutes	Unidentified	DOI (20 mm)	*S. epidermidis*		[77]
*Petromica citrina*	Cagarras Archipelago, Brazil (4–20 m)	*Bacillus pumilus* Pc31	Firmicutes	Unidentified	DOI (45 mm)	*S. epidermidis*		[77]
*Petromica citrina*	Cagarras Archipelago, Brazil (4–20 m)	*Bacillus pumilus* Pc32	Firmicutes	Unidentified	DOI (38 mm)	*S. epidermidis*		[77]
*Clathrina aurea*	Cagarras Archipelago, Brazil (4–20 m)	*Pseudovibrio ascidiaceicola* Ca31	Proteobacteria	Unidentified	DOI (25 mm)	*S. epidermidis*		[77]
*Paraleucilla magna*	Cagarras Archipelago, Brazil (4–20 m)	*Pseudovibrio ascidiaceicola* Pm31	Proteobacteria	Unidentified	DOI (35 mm)	*S. epidermidis*		[77]
*Mycale microsigmatosa*	Cagarras Archipelago, Brazil (4–20 m)	*Pseudovibrio denitrificans* Mm37	Proteobacteria	Unidentified	DOI (30 mm)	*S. epidermidis*		[77]
*Pseudoceratina clavata*	Heron Island, Australia (14 m)	*Salinispora* sp. M102, M403, M412, M413, M414, SW10, SW15, SW17	Actinobacteria	Unidentified	DOI (<5 mm)	*S. epidermidis*		[79]
*Pseudoceratina clavata*	Heron Island, Australia (14 m)	*Salinispora* sp. SW02	Actinobacteria	Unidentified	DOI (>5 mm)	*S. epidermidis*		[79]
*Callyspongia diffusa*	Bay of Bengal, India (10–15 m)	*Streptomyces* sp. CPI 13	Actinobacteria	Unidentified	DOI (6.6 mm)	*S. epidermidis*		[92]
		*Micromonospora* sp. CPI 12	Actinobacteria	Unidentified	DOI (6.6 mm)	*S. epidermidis*		[92]
		*Saccharomonospora* sp. CPI 3	Actinobacteria	Unidentified	DOI (6.3 mm)	*S. epidermidis*		[92]
*Haliclona* sp.	Cagarras Archipelago, Brazil (4–20 m)	*Pseudomonas fluorescens* H40	Proteobacteria	Unidentified	DOI (25 mm)	*S. epidermidis* 57s (susceptibile to amp, cip, pen, tet)		[77]
*Haliclona* sp.	Cagarras Archipelago, Brazil (4–20 m)	*Pseudomonas fluorescens* H41	Proteobacteria	Unidentified	DOI (25 mm)	*S. epidermidis* 57s		[77]
*Haliclona* sp.	Cagarras Archipelago, Brazil (4–20 m)	*Pseudomonas aeruginosa* H51	Proteobacteria	Unidentified	DOI (33 mm)	*S. epidermidis* 57s		[77]
*Petromica citrina*	Cagarras Archipelago, Brazil (4–20 m)	*Bacillus pumilus* Pc31	Firmicutes	Unidentified	DOI (30 mm)	*S. epidermidis* 57s		[77]
*Petromica citrina*	Cagarras Archipelago, Brazil (4–20 m)	*Bacillus pumilus* Pc32	Firmicutes	Unidentified	DOI (30 mm)	*S. epidermidis* 57s		[77]
*Clathrina aurea*	Cagarras Archipelago, Brazil (4–20 m)	*Pseudovibrio ascidiaceicola* Ca31	Proteobacteria	Unidentified	DOI (15 mm)	*S. epidermidis* 57s		[77]
*Paraleucilla magna*	Cagarras Archipelago, Brazil (4–20 m)	*Pseudovibrio ascidiaceicola* Pm31	Proteobacteria	Unidentified	DOI (17 mm)	*S. epidermidis* 57s		[77]
*Mycale microsigmatosa*	Cagarras Archipelago, Brazil (4–20 m)	*Pseudovibrio denitrificans* Mm37	Proteobacteria	Unidentified	DOI (16 mm)	*S. epidermidis* 57s		[77]
*Xestospongia testudinaria*	Weizhou coral reef, China (ND)	*Aspergillus* sp.	Ascomycota	(*Z*)-5-(Hydroxymethyl)-2-(6′-methylhept-2′-en-2′-yl)phenol	MIC (4.66 µg/mL)	*Staphylococcus albus*		[48]
				Aspergiterpenoid A	MIC (1.24 µg/mL)			
				(−)-5-(Hydroxymethyl)-2-(2′,6′,6′-trimethyltetrahydro-2*H*-pyran-2-yl)phenol	MIC (1.26 µg/mL)			
*Haliclona* sp.	Cagarras Archipelago, Brazil (4–20 m)	*Pseudomonas fluorescens* H40	Proteobacteria	Unidentified	DOI (27 mm)	*Staphylococcus haemolyticus*		[77]
*Haliclona* sp.	Cagarras Archipelago, Brazil (4–20 m)	*Pseudomonas fluorescens* H41	Proteobacteria	Unidentified	DOI (27 mm)	*S. haemolyticus*		[77]
*Haliclona* sp.	Cagarras Archipelago, Brazil (4–20 m)	*Pseudomonas aeruginosa* H51	Proteobacteria	Unidentified	DOI (35 mm)	*S. haemolyticus*		[77]
*Petromica citrina*	Cagarras Archipelago, Brazil (4–20 m)	*Bacillus pumilus* Pc31	Firmicutes	Unidentified	DOI (40 mm)	*S. haemolyticus*		[77]
*Petromica citrina*	Cagarras Archipelago, Brazil (4–20 m)	*Bacillus pumilus* Pc32	Firmicutes	Unidentified	DOI (40 mm)	*S. haemolyticus*		[77]
*Clathrina aurea*	Cagarras Archipelago, Brazil (4–20 m)	*Pseudovibrio ascidiaceicola* Ca31	Proteobacteria	Unidentified	DOI (38 mm)	*S. haemolyticus*		[77]
*Paraleucilla magna*	Cagarras Archipelago, Brazil (4–20 m)	*Pseudovibrio ascidiaceicola* Pm31	Proteobacteria	Unidentified	DOI (40 mm)	*S. haemolyticus*		[77]
*Mycale microsigmatosa*	Cagarras Archipelago, Brazil (4–20 m)	*Pseudovibrio denitrificans* Mm37	Proteobacteria	Unidentified	DOI (43 mm)	*S. haemolyticus*		[77]
*Haliclona* sp.	Cagarras Archipelago, Brazil (4–20 m)	*Pseudomonas fluorescens* H40	Proteobacteria	Unidentified	DOI (19 mm)	*S. haemolyticus* 109s (susceptible to amp, gen, oxa, pen)		[77]
*Haliclona* sp.	Cagarras Archipelago, Brazil (4–20 m)	*Pseudomonas fluorescens* H41	Proteobacteria	Unidentified	DOI (15 mm)	*S. haemolyticus* 109s		[77]
*Haliclona* sp.	Cagarras Archipelago, Brazil (4–20 m)	*Pseudomonas aeruginosa* H51	Proteobacteria	Unidentified	DOI (35 mm)	*S. haemolyticus* 109s		[77]
*Petromica citrina*	Cagarras Archipelago, Brazil (4–20 m)	*Bacillus pumilus* Pc31	Firmicutes	Unidentified	DOI (31 mm)	*S. haemolyticus* 109s		[77]
*Petromica citrina*	Cagarras Archipelago, Brazil (4–20 m)	*Bacillus pumilus* Pc32	Firmicutes	Unidentified	DOI (36 mm)	*S. haemolyticus* 109s		[77]
*Clathrina aurea*	Cagarras Archipelago, Brazil (4–20 m)	*Pseudovibrio ascidiaceicola* Ca31	Proteobacteria	Unidentified	DOI (23 mm)	*S. haemolyticus* 109s		[77]
*Paraleucilla magna*	Cagarras Archipelago, Brazil (4–20 m)	*Pseudovibrio ascidiaceicola* Pm31	Proteobacteria	Unidentified	DOI (30 mm)	*S. haemolyticus* 109s		[77]
*Mycale microsigmatosa*	Cagarras Archipelago, Brazil (4–20 m)	*Pseudovibrio denitrificans* Mm37	Proteobacteria	Unidentified	DOI (20 mm)	*S. haemolyticus* 109s		[77]
*Haliclona* sp.	Cagarras Archipelago, Brazil (4–20 m)	*Pseudomonas fluorescens* H40	Proteobacteria	Unidentified	DOI (31mm)	*Staphylococcus hominis*		[77]
*Haliclona* sp.	Cagarras Archipelago, Brazil (4–20 m)	*Pseudomonas fluorescens* H41	Proteobacteria	Unidentified	DOI (28 mm)	*S. hominis*		[77]
*Haliclona* sp.	Cagarras Archipelago, Brazil (4–20 m)	*Pseudomonas aeruginosa* H51	Proteobacteria	Unidentified	DOI (37 mm)	*S. hominis*		[77]
*Petromica citrina*	Cagarras Archipelago, Brazil (4–20 m)	*Bacillus pumilus* Pc31	Firmicutes	Unidentified	DOI (41 mm)	*S. hominis*		[77]
*Petromica citrina*	Cagarras Archipelago, Brazil (4–20 m)	*Bacillus pumilus* Pc32	Firmicutes	Unidentified	DOI (43 mm)	*S. hominis*		[77]
*Clathrina aurea*	Cagarras Archipelago, Brazil (4–20 m)	*Pseudovibrio ascidiaceicola* Ca31	Proteobacteria	Unidentified	DOI (23 mm)	*S. hominis*		[77]
*Paraleucilla magna*	Cagarras Archipelago, Brazil (4–20 m)	*Pseudovibrio ascidiaceicola* Pm31	Proteobacteria	Unidentified	DOI (25 mm)	*S. hominis*		[77]
*Mycale microsigmatosa*	Cagarras Archipelago, Brazil (4–20 m)	*Pseudovibrio denitrificans* Mm37	Proteobacteria	Unidentified	DOI (24 mm)	*S. hominis*		[77]
*Haliclona* sp.	Cagarras Archipelago, Brazil (4–20 m)	*Pseudomonas fluorescens* H40	Proteobacteria	Unidentified	DOI (25 mm)	*Staphylococcus hominis* 79s (susceptible to amp, pen)		[77]
*Haliclona* sp.	Cagarras Archipelago, Brazil (4–20 m)	*Pseudomonas fluorescens* H41	Proteobacteria	Unidentified	DOI (27 mm)	*S. hominis* 79s		[77]
*Haliclona* sp.	Cagarras Archipelago, Brazil (4–20 m)	*Pseudomonas aeruginosa* H51	Proteobacteria	Unidentified	DOI (20 mm)	*S. hominis* 79s		[77]
*Petromica citrina*	Cagarras Archipelago, Brazil (4–20 m)	*Bacillus pumilus* Pc31	Firmicutes	Unidentified	DOI (35 mm)	*S. hominis* 79s		[77]
*Petromica citrina*	Cagarras Archipelago, Brazil (4–20 m)	*Bacillus pumilus* Pc32	Firmicutes	Unidentified	DOI (30 mm)	*S. hominis* 79s		[77]
*Clathrina aurea*	Cagarras Archipelago, Brazil (4–20 m)	*Pseudovibrio ascidiaceicola* Ca31	Proteobacteria	Unidentified	DOI (25 mm)	*S. hominis* 79s		[77]
*Paraleucilla magna*	Cagarras Archipelago, Brazil (4–20 m)	*Pseudovibrio ascidiaceicola* Pm31	Proteobacteria	Unidentified	DOI (25 mm)	*S. hominis* 79s		[77]
*Mycale microsigmatosa*	Cagarras Archipelago, Brazil (4–20 m)	*Pseudovibrio denitrificans* Mm37	Proteobacteria	Unidentified	DOI (28 mm)	*S. hominis* 79s		[77]
*Xestospongia testudinaria*	Bidong Island, Malaysia (ND)	*Serratia marcescens* IBRL USM 84	Proteobacteria	Prodigiosin	DOI (≤9 mm)	*Staphylococcus saprophyticus*		[66]
*Halichondria panicea*	Kiel Fjord, Baltic Sea, Germany (ND)	*Streptomyces* sp. HB202	Actinobacteria	Mayamycin	IC_50_ (3.71 µg/mL)	*Staphylococcus lentus*		[45]
*Halichondria panicea*	Kiel Fjord, Baltic Sea, Germany (ND)	*Streptomyces* sp. HB062	Actinobacteria	Unidentified	ND	*S. lentus*		[44]
*Halichondria panicea*	Kiel Fjord, Baltic Sea, Germany (ND)	*Streptomyces* sp. HB117	Actinobacteria	Unidentified	ND	*S. lentus*		[44]
*Halichondria panicea*	Kiel Fjord, Baltic Sea, Germany (ND)	*Streptomyces* sp. HB122	Actinobacteria	Unidentified	ND	*S. lentus*		[44]
*Halichondria panicea*	Kiel Fjord, Baltic Sea, Germany (ND)	*Streptomyces* sp. HB132	Actinobacteria	Unidentified	ND	*S. lentus*		[44]
*Halichondria panicea*	Kiel Fjord, Baltic Sea, Germany (ND)	*Streptomyces* sp. HB138	Actinobacteria	Unidentified	ND	*S. lentus*		[44]
*Halichondria panicea*	Kiel Fjord, Baltic Sea, Germany (ND)	*Streptomyces* sp. HB149	Actinobacteria	Unidentified	ND	*S. lentus*		[44]
*Halichondria panicea*	Kiel Fjord, Baltic Sea, Germany (ND)	*Streptomyces* sp. HB184	Actinobacteria	Unidentified	ND	*S. lentus*		[44]
*Halichondria panicea*	Kiel Fjord, Baltic Sea, Germany (ND)	*Streptomyces* sp. HB253	Actinobacteria	Unidentified	ND	*S. lentus*		[44]
*Halichondria panicea*	Kiel Fjord, Baltic Sea, Germany (ND)	*Streptomyces* sp. HB272	Actinobacteria	Unidentified	ND	*S. lentus*		[44]
*Halichondria panicea*	Kiel Fjord, Baltic Sea, Germany (ND)	*Streptomyces* sp. HB288	Actinobacteria	Unidentified	ND	*S. lentus*		[44]
*Halichondria panicea*	Kiel Fjord, Baltic Sea, Germany (ND)	*Streptomyces* sp. HB298	Actinobacteria	Unidentified	ND	*S. lentus*		[44]
*Halichondria panicea*	Kiel Fjord, Baltic Sea, Germany (ND)	*Streptomyces* sp. HB328	Actinobacteria	Unidentified	ND	*S. lentus*		[44]
**	Kiel Fjord, Baltic Sea, Germany (ND)	*Streptomyces* sp. HB375	Actinobacteria	Unidentified	ND	*S. lentus*		[44]
*Halichondria panicea*	Kiel Fjord, Baltic Sea, Germany (ND)	*Streptomyces* sp. HB383	Actinobacteria	Unidentified	ND	*S. lentus*		[44]
*Dendrilla nigra*	Southwest Coast of India (10-12 m)	*Nocardiopsis dassonvillei* MAD08	Actinobacteria	Unidentified	MIC (600 µg/mL)	*Staphylococcus* sp. PC8		[93]
*Halichondria japonica*	Iriomote island, Japan (ND)	*Bacillus cereus* QNO3323	Firmicutes	Thiopeptide YM-266183	MIC (1.56 µg/mL)	Methicillin-Resistant *Streptococcus epidermidis* (MRSE)		[40,41]
*Halichondria japonica*	Iriomote island, Japan (ND)	*Bacillus cereus* QNO3323	Firmicutes	Thiopeptide YM-266184	MIC (0.2 µg/mL)	MRSE		[40,41]
*Aplysina aerophoba*	Banyuls-sur-Mer, France (5–15 m)	*Bacillus subtilis* A202	Firmicutes	Iturin	ND	Multi drug-resistant *S. epidermidis*		[68]
*Dysidea granulosa*	Kavaratti Island, India (ND)	*Enterobacter* sp. TTAG	Proteobacteria	Unidentified	DOI (23 mm), MIC crude extract (5 mg/mL)	*Streptococcus* sp.	[85]	
*Petrosia* sp.	Jeju island, Korea (20 m)	*Aspergillus versicolor*	Ascomycota	Averantin	MIC (0.78 µg/mL)	*Streptococcus pyogenes* 308A	[58]	
*Petrosia* sp.	Jeju island, Korea (20 m)	*Aspergillus versicolor*	Ascomycota	Nidurufin	MIC (3.13 µg/mL)	*Streptococcus pyogenes* 308A	[58]	
*Petrosia* sp.	Jeju island, Korea (20 m)	*Aspergillus versicolor*	Ascomycota	Averantin	MIC (3.13 µg/mL)	*Streptococcus pyogenes* 77A	[58]	
*Petrosia* sp.	Jeju island, Korea (20 m)	*Aspergillus versicolor*	Ascomycota	Nidurufin	MIC (6.25 µg/mL)	*Streptococcus pyogenes* 77A	[58]	
*Halichondria* sp.	West Coast of India (10 m)	*Bacillus licheniformis* SAB1	Firmicutes	Indole	DOI (1–3 mm)	*Streptococcus pyogenes*	[69]	
*Halichondria* sp.	West Coast of India (10 m)	*Bacillus licheniformis* SAB1	Firmicutes	3-Phenylpropionic	DOI (4–6 mm)	*Streptococcus pyogenes*	[69]	
*Haliclona simulans*	Gurraig Sound Kilkieran Bay, Ireland (15 m)	*Streptomyces* sp. SM2 and SM4	Actinobacteria	Unidentified	ND	*Streptococcus pneumoniae*	[88]	
*Callyspongia diffusa*	Bay of Bengal, India (10–15 m)	*Saccharomonospora* sp. CPI 9	Actinobacteria	Unidentified	ND	haemolytic* Streptococcus* sp (6.3)	[92]	
*Halichondria japonica*	Iriomote island, Japan (ND)	*Bacillus cereus* QNO3323	Firmicutes	Thiopeptide YM-266183	MIC (1.56 µg/mL)	*Bacillus subtilis* ATCC 633	[40,41]	
*Halichondria japonica*	Iriomote island, Japan (ND)	*Bacillus cereus* QNO3323	Firmicutes	Thiopeptide YM-266184	MIC (1.56 µg/mL)	*B. subtilis* ATCC 633	[40,41]	
*Halichondria panicea*	Kiel Fjord, Baltic Sea, Germany (ND)	*Streptomyces* sp. HB202	Actinobacteria	Mayamycin	IC_50_ (3.71 µg/mL)	*B. subtilis*	[45]	
*Halichondria panicea*	Kiel Fjord, Baltic Sea, Germany (ND)	*Streptomyces* sp. HB202	Actinobacteria	Streptophenazines G	IC_50_ (3.49 ± 0.38 µg/mL)	*B. subtilis*	[89]	
*Halichondria panacea*	Kiel Fjord, Baltic Sea, Germany (ND)	*Streptomyces* sp. HB202	Actinobacteria	Streptophenazines K	IC_50_ (9.18 ± 2.89 µg/mL)	*B. subtilis*	[89]	
*Halichondria panicea*	Kiel Fjord, Baltic Sea, Germany (ND)	*Streptomyces* sp. HB084	Actinobacteria	Unidentified	ND	*B. subtilis*	[44]	
*Halichondria panicea*	Kiel Fjord, Baltic Sea, Germany (ND)	*Streptomyces* sp. HB095	Actinobacteria	Unidentified	ND	*B. subtilis*	[44]	
*Halichondria panicea*	Kiel Fjord, Baltic Sea, Germany (ND)	*Streptomyces* sp. HB096	Actinobacteria	Unidentified	ND	*B. subtilis*	[44]	
*Halichondria panicea*	Kiel Fjord, Baltic Sea, Germany (ND)	*Streptomyces* sp. HB105	Actinobacteria	Unidentified	ND	*B. subtilis*	[44]	
*Halichondria panicea*	Kiel Fjord, Baltic Sea, Germany (ND)	*Streptomyces* sp. HB107	Actinobacteria	Unidentified	ND	*B. subtilis*	[44]	
*Halichondria panicea*	Kiel Fjord, Baltic Sea, Germany (ND)	*Streptomyces* sp. HB116	Actinobacteria	Unidentified	ND	*B. subtilis*	[44]	
*Halichondria panicea*	Kiel Fjord, Baltic Sea, Germany (ND)	*Streptomyces* sp. HB117	Actinobacteria	Unidentified	ND	*B. subtilis*	[44]	
*Halichondria panicea*	Kiel Fjord, Baltic Sea, Germany (ND)	*Streptomyces* sp. HB118	Actinobacteria	Unidentified	ND	*B. subtilis*	[44]	
*Halichondria panicea*	Kiel Fjord, Baltic Sea, Germany (ND)	*Streptomyces* sp. HB122	Actinobacteria	Unidentified	ND	*B. subtilis*	[44]	
*Halichondria panicea*	Kiel Fjord, Baltic Sea, Germany (ND)	*Streptomyces* sp. HB132	Actinobacteria	Unidentified	ND	*B. subtilis*	[44]	
*Halichondria panicea*	Kiel Fjord, Baltic Sea, Germany (ND)	*Streptomyces* sp. HB138	Actinobacteria	Unidentified	ND	*B. subtilis*	[44]	
*Halichondria panicea*	Kiel Fjord, Baltic Sea, Germany (ND)	*Streptomyces* sp. HB181	Actinobacteria	Unidentified	ND	*B. subtilis*	[44]	
*Halichondria panicea*	Kiel Fjord, Baltic Sea, Germany (ND)	*Streptomyces* sp. HB184	Actinobacteria	Unidentified	ND	*B. subtilis*	[44]	
*Halichondria panicea*	Kiel Fjord, Baltic Sea, Germany (ND)	*Streptomyces* sp. HB202	Actinobacteria	Unidentified	ND	*B. subtilis*	[44]	
*Halichondria panicea*	Kiel Fjord, Baltic Sea, Germany (ND)	*Streptomyces* sp. HB253	Actinobacteria	Unidentified	ND	*B. subtilis*	[44]	
*Halichondria panicea*	Kiel Fjord, Baltic Sea, Germany (ND)	*Streptomyces* sp. HB272	Actinobacteria	Unidentified	ND	*B. subtilis*	[44]	
*Halichondria panicea*	Kiel Fjord, Baltic Sea, Germany (ND)	*Streptomyces* sp. HB298	Actinobacteria	Unidentified	ND	*B. subtilis*	[44]	
*Halichondria panicea*	Kiel Fjord, Baltic Sea, Germany (ND)	*Streptomyces* sp. HB328	Actinobacteria	Unidentified	ND	*B. subtilis*	[44]	
*Halichondria panicea*	Kiel Fjord, Baltic Sea, Germany (ND)	*Streptomyces* sp. HB375	Actinobacteria	Unidentified	ND	*B. subtilis*	[44]	
*Halichondria panicea*	Kiel Fjord, Baltic Sea, Germany (ND)	*Streptomyces* sp. HB383	Actinobacteria	Unidentified	ND	*B. subtilis*	[44]	
*Callyspongia* sp.	Kyung-Po beach, Korea (12 m)	*Brevibacterium* sp. KMD 003	Actinobacteria	6-Hydroxymethyl-1-phenazine-carboxamide	MIC (5.06 µg/mL)	*B. subtilis*	[94]	
		*Brevibacterium* sp. KMD 003		1,6-Phenazinedimethanol	MIC (4.80 µg/mL)	*B. subtilis*	[94]	
*Haliclona simulans*	Gurraig Sound Kilkieran Bay, Ireland (15 m)	*Streptomyces* sp. SM8	Actinobacteria	Mixture Kitamycin A or B and Antimycin A3 or A7	MIC (7.42 µg/mL)	*B. subtilis*	[95]	
				Antimycin A2, A8, A11 or A17	MIC (9.40 µg/mL)			
				Antimycin A3 or A7	MIC (400 µg/mL)			
				Antimycin A2, A8, A11 or A17, antimycin A3 or A7	MIC (400 µg/mL)			
*Hymeniacidon perleve*	Bohai Sea, China (ND)	*Aspergillus versicolor* MF359	Ascomycota	5-Methoxydihydrosterigmatocystin	MIC (3.125 µg/mL)	*B. subtilis*	[56]	
*Hymeniacidon perleve*	Nanji island, China (ND)	*Pseudoalteromonas piscicida* NJ6-3-1	Proteobacteria	Norharman (beta-carboline alkaloid)	MIC (50 µg/mL)	*B. subtilis*	[59]	
*Xestospongia testudinaria*	Weizhou coral reef, China (ND)	*Aspergillus* sp.	Ascomycota	(−)-Sydonic acid	MIC (0.66 µg/mL)	*B. subtilis*	[48]	
				(*Z*)-5-(Hydroxymethyl)-2-(6′-methylhept-2′-en-2′-yl)phenol	MIC (2.33 µg/mL)			
				(−)-5-(Hydroxymethyl)-2-(2′,6′,6′-trimethyltetrahydro-2*H*-pyran-2-yl)phenol	MIC (0.62 µg/mL)			
*Dysidea herbacea*	Koror, Republic Palau (1 m)	*Oscillatoria spongeliae*	Cyanobacteria	2-(2′,4′-Dibromophenyl)-4,6-dibromophenol	ND	*B. subtilis*	[64]	
*Hyrtios altum*	Aragusuku island, Japan (ND)	*Vibrio* sp.	Proteobacteria	Trisindoline	DOI (17 mm)	*B. subtilis*	[65]	
*Xestospongia testudinaria*	Bidong Island, Malaysia (ND)	*Serratia marcescens* IBRL USM 84	Proteobacteria	Prodigiosin	DOI (<9 mm)	*B. subtilis*	[66]	
*Niphates olemda*	Bali Bata National Park, Indonesia (ND)	*Curvularia lunata*	Ascomycota	1,3,8-Trihydroxy-6-methoxyanthraquinone (lunatin)	DOI (9 mm)	*B. subtilis*	[70]	
				Bisanthraquinone cytoskyrin A	DOI (12 mm)	*B. subtilis*		
*Hymeniacidon perleve*	Nanji Island, China (ND)	*Pseudomonas* sp. NJ6-3-1	Proteobacteria	Unidentified	5 mm	*B. subtilis*	[74]	
*Axinella dissimilis*	Gurraig Sound, Kilkieran Bay, Ireland (15 m)	*Pseudovibrio* Ad30	Proteobacteria	Unidentified	ND	*B. subtilis*	[78]	
*Pseudoceratina clavata*	Heron Island, Great Barrier Reef (14 m)	*Salinispora* sp. M102, M403, M412, M413, M414, SW02, SW10, SW 15 and SW 17	Actinobacteria	Unidentified	ND	*B. subtilis*	[79]	
*Dendrilla nigra*	Southeast coast of India (ND)	*Streptomyces* sp. BTL7	Actinobacteria	Unidentified	DOI (15 mm)	*B. subtilis*	[80]	
*Mycale* sp.	Gulei Port, Fujian, China (ND)	*Bacillus* sp. HNS004 HNS015;	Firmicutes	Unidentified	DOI (8–10 mm)	*B. subtilis*	[81]	
*Mycale* sp.	Gulei Port, Fujian, China (ND)	*Pseudomonas* sp. HNS021; HNS027; *Vibrio* sp. HNS038	Proteobacteria	Unidentified	DOI (8–10 mm)	*B. subtilis*	[81]	
*Mycale* sp.	Gulei Port, Fujian, China (ND)	*Labrenzia* sp. HNS063; *Streptomyces* sp. HNS047; *Nocardiopsis* sp. HNS048, HNS055, HNS058;* Cobetia* sp. HNS023,	Actinobacteria	Unidentified	DOI (8–10 mm)	*B. subtilis*	[81]	
*Mycale* sp.	Gulei Port, Fujian, China (ND)	*Bacillus* sp. HNS005, HNS010,	Firmicutes	Unidentified	DOI (10–15 mm)	*B. subtilis*	[81]	
*Mycale* sp.	Gulei Port, Fujian, China (ND)	*Streptomyces* sp. HNS049, HNS056	Actinobacteria	Unidentified	DOI (10–15 mm)	*B. subtilis*	[81]	
*Mycale* sp.	Gulei Port, Fujian, China (ND)	*Vibrio* sp. HNS022, HNS029;	Firmicutes	Unidentified	DOI (15–30 mm)	*B. subtilis*	[81]	
*Mycale* sp.	Gulei Port, Fujian, China (ND)	*Streptomyces* sp. HNS054	Actinobacteria	Unidentified	DOI (15–30 mm)	*B. subtilis*	[81]	
*Sigmadocia fibulatus*	Hare Island, India (5-10 m)	*Bacillus* sp. SC3	Firmicutes	Unidentified	ND	*B. subtilis*	[96]	
*Amphilectus fucorum*	Lough Hyne, Ireland (8–15 m)	*Pseudovibrio* sp. 113V *Pseudovibrio* 83V1	Proteobacteria	Unidentified	ND	*B. subtilis*	[97]	
*Eurypon major*	Lough Hyne, Ireland (8–15 m)	*Pseudovibrio* sp. 107L, 108L, 109L	Proteobacteria	Unidentified	ND	*B. subtilis*	[97]	
*Suberites carnosus*	Lough Hyne, Co. Cork, Ireland (15 m)	*Arthrobacter* sp. W13C11	Actinobacteria	Unidentified	ND	*B. subtilis*	[83]	
*Suberites carnosus*	Lough Hyne, Co. Cork, Ireland (15 m)	*Pseudovibrio* sp. W13S4, W13S21, W13S23, W13S26, W13S31	Proteobacteria	Unidentified	ND	*B. subtilis*	[83]	
*Haliclona simulans*	Gurraig Sound, Kilkieran Bay, Ireland (15 m)	*Streptomyces* sp. SM2 and SM4	Actinobacteria	Unidentified	ND	*B. subtilis*	[88]	
*Isodictya setifera*	Ross island, Antartica (30–40 m)	*Pseudomonasaeruginosa*	Proteobacteria	Phenazine-1-carboxylic acid and phenazine-1-carboxamide	MIC (<0.49 µg/mL)	*Bacillus cereus*	[55]	
*Xestospongia testudinaria*	Weizhou coral reef, China (ND)	*Aspergillus* sp.	Ascomycota	(*Z*)-5-(Hydroxymethyl)-2-(6′-methylhept-2′-en-2′-yl)phenol	MIC (2.33 µg/mL)	*B. cereus*	[48]	
*Xestospongia testudinaria*	Bidong Island, Malaysia (ND)	*Serratia marcescens* IBRL USM 84	Proteobacteria	Prodigiosin	DOI (10–14 mm)	*B. cereus*	[66]	
*Dendrilla nigra*	Vizhinjam coast, India (10–15 m)	*Streptomyces* sp. MSI051	Actinobacteria	Unidentified	MIC (46 ± 1.62 µg protein/mL)	*B. cereus*	[73]	
*Axinella dissimilis*	Gurraig Sound, Kilkieran Bay, Ireland (15 m)	*Pseudovibrio* Ad30	Proteobacteria	Unidentified	ND	*B. cereus*	[78]	
*Dendrilla nigra*	Southeast coast of India (ND)	*Streptomyces* sp. BTL7	Actinobacteria	Unidentified	DOI (16 mm)	*B. cereus*	[80]	
*Haliclona simulans*	Gurraig Sound Kilkieran Bay, Ireland (15 m)	*Streptomyces* sp. SM2 and SM4	Actinobacteria	Unidentified	ND	*B. cereus*	[88]	
*Xestospongia testudinaria*	Bidong Island, Malaysia (ND)	*Serratia marcescens* IBRL USM 84	Proteobacteria	Prodigiosin	DOI (10–14 mm)	*Bacillus licheniformis*	[66]	
*Xestospongia testudinaria*	Bidong Island, Malaysia (ND)	*Serratia marcescens* IBRL USM 84	Proteobacteria	Prodigiosin	DOI (<9 mm)	*Bacillus thuringiensis*	[66]	
unidentified	South China Sea (10 m)	*Nocardiopsis sp.* 13-33-15 and 13-12-13	Actinobacteria	1,6-Dihydroxyphenazine	DOI (16 ± 0.5 mm)	*Bacillus mycoides* SJ14	[67]	
				1,6-Dimethoxyphenazine	DOI (20 ± 0.4 mm)			
*Aplysina aerophoba*	Banyuls-sur-Mer, France (5–15 m)	*Bacillus subtilis* A184	Firmicutes	Surfactin iturin fengycin	ND	*Bacillus megaterium*	[68]	
*Aplysina aerophoba*	Banyuls-sur-Mer, France (5–15 m)	*Bacillus subtilis* A190	Firmicutes	Surfactin	ND	*B. megaterium*	[68]	
*Aplysina aerophoba*	Banyuls-sur-Mer, France (5–15 m)	*Bacillus subtilis* A202	Firmicutes	Iturin	ND	*B. megaterium*	[68]	
*Haliclona simulans*	Gurraig Sound Kilkieran Bay, Ireland (15 m)	*Bacillus subtilis* MMA7	Firmicutes	Subtilomycin	ND	*B. megaterium*	[71]	
*Dysidea avara*	Mediterranean sea (ND)	*Actinokinespora* sp. EG49	Actinobacteria	1,6-Dihydroxyphenazine (result of the co-culture)	DOI (11 mm)	*Bacillus* sp. P25	[98]	
*Spheciospongia vagabunda*	Red Sea (ND)	*Nocardiopsis* sp. RV163						
*Callyspongia diffusa*	Bay of Bengal, India (10–15 m)	*Streptomyces* sp. CPI 13	Actinobacteria	Unidentified	DOI (6.6 mm)	*Bacillus* sp.	[92]	
*Callyspongia diffusa*	Bay of Bengal, India (10–15 m)	*Micromonospora* sp. CPI 12	Actinobacteria	Unidentified	DOI (8 mm)	*Bacillus* sp.	[92]	
*Haliclona* sp.	Cagarras Archipelago, Brazil (4–20 m)	*Pseudomonas fluorescens* H40	Proteobacteria	Unidentified	DOI (19 mm)	*Enterococcus faecalis*	[77]	
*Haliclona* sp.	Cagarras Archipelago, Brazil (4–20 m)	*Pseudomonas fluorescens* H41	Proteobacteria	Unidentified	DOI (17 mm)	*E. faecalis*	[77]	
*Haliclona* sp.	Cagarras Archipelago, Brazil (4–20 m)	*Pseudomonas aeruginosa* H51	Proteobacteria	Unidentified	DOI (32 mm)	*E. faecalis*	[77]	
*Clathrina aurea*	Cagarras Archipelago, Brazil (4–20 m)	*Pseudovibrio ascidiaceicola* Ca31	Proteobacteria	Unidentified	DOI (11 mm),	*E. faecalis*	[77]	
*Paraleucilla magna*	Cagarras Archipelago, Brazil (4–20 m)	*Pseudovibrio ascidiaceicola* Pm31	Proteobacteria	Unidentified	DOI (12 mm),	*E. faecalis*	[77]	
*Mycale microsigmatosa*	Cagarras Archipelago, Brazil (4–20 m)	*Pseudovibrio denitrificans* Mm37	Proteobacteria	Unidentified	DOI (14 mm)	*E. faecalis*	[77]	
unidentified	Rovinj, Croatia (3–20 m)	*Streptomyces* sp. RV15	Actinobacteria	Unidentified	DOI (11 mm)	*E. faecalis*	[82]	
unidentified	Rovinj, Croatia (3–20 m)	*Microbacterium* sp*.* EG69	Actinobacteria	Unidentified	DOI (9 mm)	*E. faecalis*	[82]	
unidentified	Rovinj, Croatia (3–20 m)	*Micromonospora* sp. RV115	Actinobacteria	Unidentified	DOI (10 mm)	*E. faecalis*	[82]	
unidentified	Rovinj, Croatia (3–20 m)	*Rhodococcus* sp. EG33	Actinobacteria	Unidentified	DOI (8 mm)	*E. faecalis*	[82]	
*Halocondria japonica*	Iriomote island, Japan (ND)	*Bacillus cereus* QNO3323	Firmicutes	Thiopeptide YM-266183	MIC (0.1 µg/mL)	*E. faecalis* CAY 04_1	[40,41]	
*Halocondria japonica*	Iriomote island, Japan (ND)	*Bacillus cereus* QNO3323	Firmicutes	Thiopeptide YM-266184	MIC (0.025 µg/mL)	*E. faecalis* CAY 04_1	[40,41]	
*Spheciospongia vagabunda*	Red Sea (ND)	*Micrococcus* sp. EG45	Actinobacteria	Microluside A	MIC (9.55 µg/mL)	*E. faecalis* JH212	[54]	
*Haliclona* sp.	Cagarras Archipelago, Brazil (4–20 m)	*Pseudomonas fluorescens* H41	Proteobacteria	Unidentified	DOI (20 mm)	*E. faecalis* 5AE (susceptible to van)	[77]	
*Clathrina aurea*	Cagarras Archipelago, Brazil (4–20 m)	*Pseudovibrio ascidiaceicola* Ca31	Proteobacteria	Unidentified	DOI (12 mm)	*E. faecalis* 5AE	[77]	
*Mycale microsigmatosa*	Cagarras Archipelago, Brazil (4–20 m)	*Pseudovibrio denitrificans* Mm37	Proteobacteria	Unidentified	DOI (15 mm)	*E. faecalis* 5AE	[77]	
*Halocondria japonica*	Iriomote island, Japan (ND)	*Bacillus cereus* QNO3323	Firmicutes	Thiopeptide YM-266183	MIC 0.2 µg/mL	*Enterococcus faecium* CAY 09_1	[40,41]	
*Halocondria japonica*	Iriomote island, Japan (ND)	*Bacillus cereus* QNO3323	Firmicutes	Thiopeptide YM-266184	MIC (0.05 µg/mL)	*E. faecium* CAY 09_1	[40,41]	
*Halocondria japonica*	Iriomote island, Japan (ND)	*Bacillus cereus* QNO3323	Firmicutes	Thiopeptide YM-266183	MIC (0.025 µg/mL)	Vancomycin-Resistant *E. faecium* CAY 09_2	[40,41]	
*Halocondria japonica*	Iriomote island, Japan (ND)	*Bacillus cereus* QNO3323	Firmicutes	Thiopeptide YM-266184	MIC (0.025 µg/mL)	Vancomycin-Resistant *E. faecium* CAY 09_2	[40,41]	
*Melophus* sp.	Lau group, Fiji islands (10 m)	*Penicillium* sp. FF001	Ascomycota	Citrinin	MIC (1.95 µg/mL)	Vancomycin-resistant *E. faecium*	[57]	
*Haliclona simulans*	Gurraig Sound Kilkieran Bay, Ireland (15 m)	*Bacillus subtilis* MMA7	Firmicutes	Subtilomycin	ND	*E. faecium*	[71]	
*Haliclona* sp.	Cagarras Archipelago, Brazil (4–20 m)	*Pseudomonas fluorescens* H40	Proteobacteria	Unidentified	DOI (18 mm)	*E. faecium*	[77]	
*Haliclona* sp.	Cagarras Archipelago, Brazil (4–20 m)	*Pseudomonas fluorescens* H41	Proteobacteria	Unidentified	DOI (21 mm)	*E. faecium*	[77]	
*Haliclona* sp.	Cagarras Archipelago, Brazil (4–20 m)	*Pseudomonas aeruginosa* H51	Proteobacteria	Unidentified	DOI (30 mm)	*E. faecium*	[77]	
*Dragmacidon* *reticulatus*	Cagarras Archipelago, Brazil (4–20 m)	*Bacillus pumilus* Dr31	Firmicutes	Unidentified	DOI (20 mm)	*E. faecium*	[77]	
*Petromica citrina*	Cagarras Archipelago, Brazil (4–20 m)	*Bacillus pumilus* Pc31	Firmicutes	Unidentified	DOI (23 mm)	*E. faecium*	[77]	
*Petromica citrina*	Cagarras Archipelago, Brazil (4–20 m)	*Bacillus pumilus* Pc32	Firmicutes	Unidentified	DOI (20 mm)	*E. faecium*	[77]	
*Clathrina aurea*	Cagarras Archipelago, Brazil (4–20 m)	*Pseudovibrio ascidiaceicola* Ca31	Proteobacteria	Unidentified	DOI (22 mm)	*E. faecium*	[77]	
*Paraleucilla magna*	Cagarras Archipelago, Brazil (4–20 m)	*Pseudovibrio ascidiaceicola* Pm31	Proteobacteria	Unidentified	DOI (20 mm)	*E. faecium*	[77]	
*Mycale microsigmatosa*	Cagarras Archipelago, Brazil (4–20 m)	*Pseudovibrio denitrificans* Mm37	Proteobacteria	Unidentified	DOI (15 mm)	*E. faecium*	[77]	
*Axinella dissimilis*	Gurraig Sound, Kilkieran Bay, Ireland (15 m)	*Pseudovibrio* Ad30	Proteobacteria	Unidentified	ND	*E. faecium*	[78]	
*Axinella dissimilis*	Gurraig Sound, Kilkieran Bay, Ireland (15 m)	*Pseudovibrio* Ad30	Proteobacteria	Unidentified	ND	Vancomycin-resistant *Enterococcus* sp.	[78]	
*Callyspongia* sp.	Kyung-Po beach, Korea (12 m)	*Brevibacterium* sp. KMD 003	Actinobacteria	6-Hydroxymethyl-1-phenazine-carboxamide	MIC (1.26 µg/mL)	*Enterococcus hirae*	[94]	
				1,6-Phenazinedimethanol	MIC (1.20 µg/mL)	*E. hirae*	[94]	
*Haliclona* sp.	Cagarras Archipelago, Brazil (4–20 m)	*Pseudomonas fluorescens* H40	Proteobacteria	Unidentified	DOI (22 mm)	*Enterobacter cloacae*	[77]	
*Haliclona* sp.	Cagarras Archipelago, Brazil (4–20 m)	*Pseudomonas fluorescens* H41	Proteobacteria	Unidentified	DOI (25 mm)	*E. cloacae*	[77]	
*Haliclona* sp.	Cagarras Archipelago, Brazil (4–20 m)	*Pseudomonas aeruginosa* H51	Proteobacteria	Unidentified	DOI (18 mm)	*E. cloacae*	[77]	
*Callyspongia diffusa*	Southwest Coast of India (6–7 m)	*Shewanella algae* VCDB KC623651	Proteobacteria	Unidentified	DOI (11mm)	*E. cloacae*	[99]	
*Haliclona* sp.	Cagarras Archipelago, Brazil (4–20 m)	*Pseudomonas fluorescens* H40	Proteobacteria	Unidentified	DOI (19 mm)	*E. cloacae* AE (susceptible to amp, cef, fox, tet)	[77]	
*Haliclona* sp.	Cagarras Archipelago, Brazil (4–20 m)	*Pseudomonas fluorescens* H41	Proteobacteria	Unidentified	DOI (12 mm)	*E. cloacae* AE	[77]	
*Haliclona* sp.	Cagarras Archipelago, Brazil (4–20 m)	*Pseudomonas aeruginosa* H51	Proteobacteria	Unidentified	DOI (23 mm)	*E. cloacae* AE	[77]	
*Petromica citrina*	Cagarras Archipelago, Brazil (4–20 m)	*Bacillus pumilus* Pc31	Firmicutes	Unidentified	DOI (20 mm)	*E. cloacae* AE	[77]	
*Petromica citrina*	Cagarras Archipelago, Brazil (4–20 m)	*Bacillus pumilus* Pc32	Firmicutes	Unidentified	DOI (20 mm)	*E. cloacae* AE	[77]	
*Haliclona* sp.	Cagarras Archipelago, Brazil (4–20 m)	*Pseudomonas fluorescens* H40	Proteobacteria	Unidentified	DOI (28 mm)	*Enterobacter hafniae*		
*Haliclona* sp.	Cagarras Archipelago, Brazil (4–20 m)	*Pseudomonas fluorescens* H41	Proteobacteria	Unidentified	DOI (21 mm)	*E. hafniae*	[77]	
*Haliclona* sp.	Cagarras Archipelago, Brazil (4–20 m)	*Pseudomonas aeruginosa* H51	Proteobacteria	Unidentified	DOI (23 mm)	*E. hafniae*	[77]	
*Petromica citrina*	Cagarras Archipelago, Brazil (4–20 m)	*Bacillus pumilus* Pc31	Firmicutes	Unidentified	DOI (18 mm)	*E. hafniae*	[77]	
*Axinella dissimilis*	Gurraig Sound, Kilkieran Bay, Ireland (15 m)	*Pseudovibrio* Ad30	Proteobacteria	Unidentified	ND	*Enterobacter aerogenes*	[78]	
*Xestospongia testudinaria*	Weizhou coral reef, China (ND)	*Aspergillus* sp.	Ascomycota	(−)-Sydonic acid	MIC (1.33 µg/mL	*Escherichia coli*	[48]	
				(*Z*)-5-(Hydroxymethyl)-2-(6′-methylhept-2′-en-2′-yl)phenol	MIC (2.33 µg/mL)			
				Aspergiterpenoid A	MIC (4.72 µg/mL			
				(−)-Sydonol	MIC (5.04 µg/mL)			
*Halocondria japonica*	Iriomote island, Japan (ND)	*Bacillus cereus* QNO3323	Firmicutes	Thiopeptide YM-266183	MIC (>100 µg/mL)	*E. coli* JCM 5491	[40,41]	
*Halocondria japonica*	Iriomote island, Japan (ND)	*Bacillus cereus* QNO3323	Firmicutes	Thiopeptide YM-266184	MIC (>100 µg/mL)	*E. coli* JCM 5491	[40,41]	
unidentified sponge	Vizhijam coast (10–12 m)	*Aspergillus clavatus* MFD15	Ascomycota	1*H*-1,2,4-Triazole-3-carboxaldehyde 5-methyl	MIC (800 ± 10 µg/mL)	*E. coli*	[91]	
*Spongia officinalis*	Southeast Coast India (10–15 m)	*Streptomyces* sp. MAPS15	Actinobacteria	2-Pyrrolidone	MIC (400 µg/mL)	*E. coli* PC1	[63]	
*Dysidea herbacea*	Koror, Republic Palau (1 m)	*Oscillatoria spongeliae*	Cyanobacteria	2-(2′,4′-Dibromophenyl)-4,6-dibromophenol	ND	*E. coli*	[64]	
*Hyrtios altum*	Aragusuku island, Japan (ND)	*Vibrio sp*	Proteobacteria	Trisindoline	DOI (16 mm)	*E. coli*	[65]	
*Xestospongia testudinaria*	Bidong Island, Malaysia (ND)	*Serratia marcescens* IBRL USM 84	Proteobacteria	Prodigiosin	DOI (≤9 mm)	*E. coli*	[66]	
unidentified	South China Sea (10 m)	*Nocardiopsis sp.* 13-33-15 and 13-12-13	Actinobacteria	1,6-Dihydroxyphenazine	DOI (8 ± 0.4 mm)	*E. coli* SJ42	[67]	
unidentified	South China Sea (10 m)	*Nocardiopsis sp.* 13-33-15 and 13-12-13	Actinobacteria	1,6-Dimethoxyphenazine	DOI (10 ± 0.6mm)	*E. coli* SJ42	[67]	
*Aplysina aerophoba*	Banyuls-sur-Mer, France (5–15 m)	*Bacillus subtilis* A184	Firmicutes	Surfactin Iturin Fengycin	ND	*E. coli*	[68]	
*Aplysina aerophoba*	Banyuls-sur-Mer, France (5–15 m)	*Bacillus subtilis* A190	Firmicutes	Surfactin	ND	*E. coli*	[68]	
*Aplysina aerophoba*	Banyuls-sur-Mer, France (5–15 m)	*Bacillus subtilis* A202	Firmicutes	Iturin	ND	*E. coli*	[68]	
*Niphates olemda*	Bali Bata National Park, Indonesia (ND)	*Curvularia lunata*	Ascomycota	1,3,8-Trihydroxy-6-methoxyanthraquinone (lunatin)	DOI (11 mm)	*E. coli*	[70]	
*Niphates olemda*	Bali Bata National Park, Indonesia (ND)	*Curvularia lunata*	Ascomycota	Bisanthraquinone cytoskyrin A	DOI (11 mm)	*E. coli*	[70]	
*Niphates olemda*	Bali Bata National Park, Indonesia (ND)	*Curvularia lunata*	Ascomycota	1,3,8-Trihydroxy-6-methoxyanthraquinone (lunatin)	DOI (10.5 mm)	*E.coli* HBI-101	[70]	
*Niphates olemda*	Bali Bata National Park, Indonesia (ND)	*Curvularia lunata*	Ascomycota	Bisanthraquinone cytoskyrin A	DOI (9 mm)	*E.coli* HBI-101	[70]	
*Polymastia boletiformis*, *Axinella dissimilis* and *Haliclona simulans*	Gurraig Sound, Kilkieran Bay, Ireland (15 m)	*Pseudovibrio* sp. W64, W69, W89, W74	Proteobacteria	Tropodithietic acid	DOI (≥ 2 mm)	*E. coli*	[72]	
*Polymastia boletiformis*, *Axinella dissimilis* and *Haliclona simulans*	Gurraig Sound, Kilkieran Bay, Ireland (15 m)	*Pseudovibrio* sp. JIC5, JIC6, JIC17, W62, W65, W71, W99, WC43, W85, W78, W94, W96, WM31, WM33, WM34, WM40, WC13, WC21, WC22, WC30, WC32, WC41, HC6, HMMA3	Proteobacteria	Unidentified	DOI (≥2 mm)	*E. coli*	[72]	
*Dendrilla nigra*	Southwest Coast of India	*Nocardiopsis dassonvillei* MAD08	Actinobacteria	Unidentified	MIC (300 µg/mL)	*E. coli* PC1	[93]	
*Hymeniacidon perleve*	Nanji Island, China (ND)	*Pseudomonas* sp. NJ6-3-1	Proteobacteria	Unidentified	DOI (1–3 mm)	*E. coli*	[74]	
*Callyspongia* spp	Kovalam Coast, India (5–10 m)	*Aspergillus flavus* GU815344	Ascomycota	Unidentified	DOI (42 mm)	*E. coli*	[75]	
*Haliclona* sp.	Cagarras Archipelago, Brazil (4–20 m)	*Pseudomonas fluorescens* H40	Proteobacteria	Unidentified	DOI (25 mm)	*E. coli*	[77]	
*Haliclona* sp.	Cagarras Archipelago, Brazil (4–20 m)	*Pseudomonas fluorescens* H41	Proteobacteria	Unidentified	DOI (15 mm)	*E. coli*	[77]	
*Haliclona* sp.	Cagarras Archipelago, Brazil (4–20 m)	*Pseudomonas aeruginosa* H51	Proteobacteria	Unidentified	DOI (22 mm)	*E. coli*	[77]	
*Petromica citrina*	Cagarras Archipelago, Brazil (4–20 m)	*Bacillus pumilus* Pc31	Proteobacteria	Unidentified	DOI (18 mm)	*E. coli*	[77]	
*Petromica citrina*	Cagarras Archipelago, Brazil (4–20 m)	*Bacillus pumilus* Pc32	Proteobacteria	Unidentified	DOI (16 mm)	*E. coli*	[77]	
*Mycale microsigmatosa*	Cagarras Archipelago, Brazil (4–20 m)	*Pseudovibrio denitrificans* Mm37	Proteobacteria	Unidentified	DOI (12 mm)	*E. coli*	[77]	
*Axinella dissimilis*	Gurraig Sound, Kilkieran Bay, Ireland (15 m)	*Pseudovibrio* Ad30	Proteobacteria	Unidentified	ND	*E. coli*	[78]	
*Dendrilla nigra*	Southeast coast of India (ND)	*Streptomyces* sp. BTL7	Actinobacteria	Unidentified	DOI (16 mm)	*E. coli*	[80]	
*Mycale* sp.	Gulei Port, Fujian, China (ND)	*Bacillus* sp. HNS005	Firmicutes	Unidentified	DOI (8–10 mm)	*E. coli*	[81]	
*Mycale* sp.	Gulei Port, Fujian, China (ND)	*Vibrio* sp. HNS038;	Proteobacteria	Unidentified	DOI (8–10 mm)	*E. coli*	[81]	
*Mycale* sp.	Gulei Port, Fujian, China (ND)	*Streptomyces* sp. HNS047; *Nocardiopsis* sp. HNS051, HNS055, HNS056	Actinobacteria			**		
*Mycale* sp.	Gulei Port, Fujian, China (ND)	*Streptomyces* sp. HNS054; *Nocardiopsis* sp. HNS058	Actinobacteria	Unidentified	DOI (10–15 mm)	*E. coli*	[81]	
*Callyspongia diffusa*	Bay of Bengal, India (10–15 m)	*Micromonospora* sp. CPI 12	Actinobacteria	Unidentified	DOI (7.5m )	*E. coli*	[92]	
*Sigmadocia fibulatus*	Hare Island, India (5-10 m)	*Bacillus* sp. SC3	Firmicutes	Unidentified	DOI (26 mm)	*E. coli*	[96]	
*Aplysina aerophoba* and *Aplysina cavernicola*	Marseille and Banyuls sur Mer, France (ND)	*Bacillus* SB8, SB17	Firmicutes	Unidentified	DOI (12–16 mm)	*E. coli*	[84]	
*Aplysina aerophoba* and *Aplysina cavernicola*	Marseille and Banyuls sur Mer, France (ND)	*Enterococcus* SB91	Proteobacteria	Unidentified	DOI (12–16 mm)	*E. coli*	[84]	
*Aplysina aerophoba* and *Aplysina cavernicola*	Marseille and Banyuls sur Mer, France (ND)	*Arthrobacter* SB95	Actinobacteria	Unidentified	DOI (12–16 mm)	*E. coli*	[84]	
*Aplysina aerophoba* and *Aplysina cavernicola*	Marseille and Banyuls sur Mer, France (ND)	unidentified low G + C Gram positive SB122 and SB144,	Unidentified	Unidentified	DOI (12–16 mm)	*E. coli*	[84]	
*Aplysina aerophoba* and *Aplysina cavernicola*	Marseille and Banyuls sur Mer, France (ND)	α-Proteobacteria SB6, SB55, SB63, SB89, SB156, SB197, SB202, SB207, SB214,	Proteobacteria	Unidentified	DOI (12–16 mm)	*E. coli*	[84]	
*Halichondria panicea*	Kiel Fjord, Baltic Sea, Germany (ND)	*Streptomyces* sp. HB107	Actinobacteria	Unidentified	ND	*E. coli*	[44]	
*Halichondria panicea*	Kiel Fjord, Baltic Sea, Germany (ND)	*Streptomyces* sp. HB132	Actinobacteria	Unidentified	ND	*E. coli*	[44]	
*Halichondria panicea*	Kiel Fjord, Baltic Sea, Germany (ND)	*Streptomyces* sp. HB253	Actinobacteria	Unidentified	ND	*E. coli*	[44]	
*Halichondria panicea*	Kiel Fjord, Baltic Sea, Germany (ND)	*Streptomyces* sp. HB298	Actinobacteria	Unidentified	ND	*E. coli*	[44]	
*Amphilectus fucorum*	Lough Hyne, Ireland (8–15 m)	*Pseudovibrio* sp. 117V, 115 V and 112 V	Proteobacteria	Unidentified	ND	*E. coli*	[97]	
*Amphilectus fucorum*	Lough Hyne, Ireland (8–15 m)	*Pseudovibrio* sp. 113V	Proteobacteria	Unidentified	ND	*E. coli*	[97]	
*Eurypon major*	Lough Hyne, Ireland (8–15 m)	*Pseudovibrio* sp. 107L, 108L, 109L	Proteobacteria	Unidentified	ND	*E. coli*	[97]	
*Haliclona simulans*	Gurraig Sound Kilkieran Bay, Ireland (15 m)	Streptomyces sp. SM2 and SM4	Actinobacteria	Unidentified	DOI (25 mm)	*E. coli*	[88]	
*Dysidea granulosa*	Kavaratti Island, India (ND)	*Enterobacter* sp. TTAG	Proteobacteria	Unidentified	ND	*E. coli*	[85]	
*Callyspongia diffusa*	Southwest Coast of India (6–7 m)	*Shewanella algae* VCDB KC623651	Proteobacteria	Unidentified	DOI (10 mm)	*E. coli*	[99]	
*Haliclona* sp.	Cagarras Archipelago, Brazil (4–20 m)	*Pseudomonas fluorescens* H40	Proteobacteria	Unidentified	DOI (20 mm)	*E. coli* 54AE (susceptible to amp, chl, sxt, tet)	[77]	
*Haliclona* sp.	Cagarras Archipelago, Brazil (4–20 m)	*Pseudomonas fluorescens* H41	Proteobacteria	Unidentified	DOI (17 mm)	*E. coli* 54AE	[77]	
*Haliclona* sp.	Cagarras Archipelago, Brazil (4–20 m)	*Pseudomonas aeruginosa* H51	Proteobacteria	Unidentified	DOI (20 mm)	*E. coli* 54AE	[77]	
*Petromica citrina*	Cagarras Archipelago, Brazil (4–20 m)	*Bacillus pumilus* Pc31	Firmicutes	Unidentified	DOI (35 mm)	*E. coli* 54AE	[77]	
*Petromica citrina*	Cagarras Archipelago, Brazil (4–20 m)	*Bacillus pumilus* Pc32	Firmicutes	Unidentified	DOI (39 mm)	*E. coli* 54AE	[77]	
*Clathrina aurea*	Cagarras Archipelago, Brazil (4–20 m)	*Pseudovibrio ascidiaceicola* Ca31	Proteobacteria	Unidentified	DOI (15 mm)	*E. coli* 54AE	[77]	
*Paraleucilla magna*	Cagarras Archipelago, Brazil (4–20 m)	*Pseudovibrio ascidiaceicola* Pm31	Proteobacteria	Unidentified	DOI (21 mm)	*E. coli* 54AE	[77]	
*Mycale microsigmatosa*	Cagarras Archipelago, Brazil (4–20 m)	*Pseudovibrio denitrificans* Mm37	Proteobacteria	Unidentified	DOI (25 mm)	*E. coli* 54AE	[77]	
*Petrosia ficiformis*	Paraggi, Ligurian Sea, Italy (8 m)	*Pseudoalteromonas* sp. F6	Proteobacteria	Unidentified	ND	*Escherichia faecalis*	[86]	
*Halichondria panicea*	Kiel Fjord, Baltic Sea, Germany (ND)	*Streptomyces* sp. HB202	Actinobacteria	Mayamycin	IC50 (1.16 µg/mL)	*Klebsiella pneumoniae*	[45]	
*Spongia officinalis*	Southeast Coast India (10–15 m)	*Streptomyces* sp. MAPS15	Actinobacteria	2-Pyrrolidone	MIC (700 µg/mL)	*K. pneumonia* PC7	[63]	
*Haliclona* sp.	Cagarras Archipelago, Brazil (4–20 m)	*Pseudomonas fluorescens* H40	Proteobacteria	Unidentified	DOI (25 mm)	*K. pneumoniae*	[77]	
*Haliclona* sp.	Cagarras Archipelago, Brazil (4–20 m)	*Pseudomonas fluorescens* H41	Actinobacteria	Unidentified	DOI (24 mm)	*K. pneumoniae*	[77]	
*Dendrilla nigra*	Southeast coast of India (ND)	*Streptomyces* sp. BTL7	Actinobacteria	Unidentified	DOI (<10 mm)	*K. pneumoniae*	[80]	
*Callyspongia diffusa*	Bay of Bengal, India (10–15 m)	*Streptomyces* sp. CPI 13	Actinobacteria	Unidentified	DOI (9.2 mm)	*K. pneumoniae*	[92]	
*Callyspongia diffusa*	Bay of Bengal, India (10–15 m)	*Saccharomonospora* sp. CPI 3	Actinobacteria	Unidentified	DOI (6.3 mm)	*K. pneumoniae*	[92]	
*Dysidea granulosa*	Cagarras Archipelago, Brazil (4–20 m)	*Enterobacter* sp. TTAG	Proteobacteria	Unidentified	DOI (22 mm)	*K. pneumoniae*	[85]	
*Haliclona* sp.	Cagarras Archipelago, Brazil (4–20 m)	*Pseudomonas fluorescens* H40	Proteobacteria	Unidentified	DOI (18 mm)	*K. pneumoniae* 52 AE	[77]	
*Haliclona* sp.	Cagarras Archipelago, Brazil (4–20 m)	*Pseudomonas fluorescens* H41	Proteobacteria	Unidentified	DOI (15 mm)	*K. pneumoniae* 52 AE	[77]	
*Haliclona* sp.	Cagarras Archipelago, Brazil (4–20 m)	*Pseudomonas aeruginosa* H51	Proteobacteria	Unidentified	DOI (21 mm)	*K. pneumoniae* 52 AE	[77]	
*Haliclona* sp.	Cagarras Archipelago, Brazil (4–20 m)	*Pseudomonas fluorescens* H40	Proteobacteria	Unidentified	DOI (16 mm)	*K. pneumoniae* 19AE (susceptible to amp, atm, caz, cpd, fox)	[77]	
*Haliclona* sp.	Cagarras Archipelago, Brazil (4–20 m)	*Pseudomonas fluorescens* H41	Proteobacteria	Unidentified	DOI (20 mm)	*K. pneumoniae* 19AE	[77]	
*Haliclona* sp.	Cagarras Archipelago, Brazil (4–20 m)	*Pseudomonas aeruginosa* H51	Proteobacteria	Unidentified	DOI (32 mm)	*K. pneumoniae* 19AE	[77]	
*Haliclona* sp.	Cagarras Archipelago, Brazil (4–20 m)	*Pseudomonas fluorescens* H40	Proteobacteria	Unidentified	DOI (20 mm)	*Neisseria gonorrhoeae*	[77]	
*Haliclona* sp.	Cagarras Archipelago, Brazil (4–20 m)	*Pseudomonas fluorescens* H41	Proteobacteria	Unidentified	DOI (27 mm)	*N. gonorrhoeae*	[77]	
*Haliclona* sp.	Cagarras Archipelago, Brazil (4–20 m)	*Pseudomonas aeruginosa* H51	Proteobacteria	Unidentified	DOI (52 mm)	*N. gonorrhoeae*	[77]	
*Petromica citrina*	Cagarras Archipelago, Brazil (4–20 m)	*Bacillus pumilus* Pc31	Firmicutes	Unidentified	DOI (28 mm)	*N. gonorrhoeae*	[77]	
*Petromica citrina*	Cagarras Archipelago, Brazil (4–20 m)	*Bacillus pumilus* Pc32	Firmicutes	Unidentified	DOI (29 mm)	*N. gonorrhoeae*	[77]	
*Haliclona* sp.	Cagarras Archipelago, Brazil (4–20 m)	*Pseudomonas fluorescens* H40	Proteobacteria	Unidentified	DOI (24 mm)	*N. gonorrhoeae* 4277 (susceptible to pen)	[77]	
*Haliclona* sp.	Cagarras Archipelago, Brazil (4–20 m)	*Pseudomonas fluorescens* H41	Proteobacteria	Unidentified	DOI (24 mm)	*N. gonorrhoeae* 4277	[77]	
*Haliclona* sp.	Cagarras Archipelago, Brazil (4–20 m)	*Pseudomonas aeruginosa* H51	Proteobacteria	Unidentified	DOI (32 mm)	*N. gonorrhoeae* 4277	[77]	
*Petromica citrina*	Cagarras Archipelago, Brazil (4–20 m)	*Bacillus pumilus* Pc31	Firmicutes	Unidentified	DOI (32 mm)	*N. gonorrhoeae* 4277	[77]	
*Petromica citrina*	Cagarras Archipelago, Brazil (4–20 m)	*Bacillus pumilus* Pc32	Firmicutes	Unidentified	DOI (32 mm)	*N. gonorrhoeae* 4277	[77]	
*Haliclona* sp.	Cagarras Archipelago, Brazil (4–20 m)	*Pseudomonas fluorescens* H40	Proteobacteria	Unidentified	DOI (24 mm)	*N. gonorrhoeae* 4957 (susceptible to cip)	[77]	
*Haliclona* sp.	Cagarras Archipelago, Brazil (4–20 m)	*Pseudomonas fluorescens* H41	Proteobacteria	Unidentified	DOI (29 mm)	*N. gonorrhoeae* 4957	[77]	
*Haliclona* sp.	Cagarras Archipelago, Brazil (4–20 m)	*Pseudomonas aeruginosa* H51	Proteobacteria	Unidentified	DOI (36 mm)	*N. gonorrhoeae* 4957	[77]	
*Petromica citrina*	Cagarras Archipelago, Brazil (4–20 m)	*Bacillus pumilus* Pc31	Firmicutes	Unidentified	DOI (23 mm)	*N. gonorrhoeae* 4957	[77]	
*Petromica citrina*	Cagarras Archipelago, Brazil (4–20 m)	*Bacillus pumilus* Pc32	Firmicutes	Unidentified	DOI (23 mm)	*N. gonorrhoeae* 4957	[77]	
*Haliclona* sp.	Cagarras Archipelago, Brazil (4–20 m)	*Pseudomonas fluorescens* H40	Proteobacteria	Unidentified	DOI (20 mm)	*N. gonorrhoeae* 5728 (cip, pen, tet)	[77]	
*Haliclona* sp.	Cagarras Archipelago, Brazil (4–20 m)	*Pseudomonas fluorescens* H41	Proteobacteria	Unidentified	DOI (19 mm)	*N. gonorrhoeae* 5728	[77]	
*Haliclona* sp.	Cagarras Archipelago, Brazil (4–20 m)	*Pseudomonas aeruginosa* H51	Proteobacteria	Unidentified	DOI (46 mm)	*N. gonorrhoeae* 5728	[77]	
*Petromica citrina*	Cagarras Archipelago, Brazil (4–20 m)	*Bacillus pumilus* Pc31	Firmicutes	Unidentified	DOI (29 mm)	*N. gonorrhoeae* 5728	[77]	
*Petromica citrina*	Cagarras Archipelago, Brazil (4–20 m)	*Bacillus pumilus* Pc32	Firmicutes	Unidentified	DOI (32 mm)	*N. gonorrhoeae* 5728	[77]	
*Haliclona* sp.	Cagarras Archipelago, Brazil (4–20 m)	*Pseudomonas fluorescens* H40	Proteobacteria	Unidentified	DOI (18 mm)	*N. gonorrhoeae* 5729 (susceptible to azm, pen, tet)	[77]	
*Haliclona* sp.	Cagarras Archipelago, Brazil (4–20 m)	*Pseudomonas fluorescens* H41	Proteobacteria	Unidentified	DOI (16 mm)	*N. gonorrhoeae* 5729	[77]	
*Haliclona* sp.	Cagarras Archipelago, Brazil (4–20 m)	*Pseudomonas aeruginosa* H51	Proteobacteria	Unidentified	DOI (35 mm)	*N. gonorrhoeae* 5729	[77]	
*Petromica citrina*	Cagarras Archipelago, Brazil (4–20 m)	*Bacillus pumilus* Pc31	Firmicutes	Unidentified	DOI (25 mm)	*N. gonorrhoeae* 5729	[77]	
*Petromica citrina*	Cagarras Archipelago, Brazil (4–20 m)	*Bacillus pumilus* Pc32	Firmicutes	Unidentified	DOI (32 mm)	*N. gonorrhoeae* 5729	[77]	
*Haliclona* sp.	Cagarras Archipelago, Brazil (4–20 m)	*Pseudomonas fluorescens* H40	Proteobacteria	Unidentified	DOI (20 mm)	*N. gonorrhoeae* 6002 (susceptible to tet)	[77]	
*Haliclona* sp.	Cagarras Archipelago, Brazil (4–20 m)	*Pseudomonas fluorescens* H41	Proteobacteria	Unidentified	DOI (26 mm)	*N. gonorrhoeae* 6002	[77]	
*Haliclona* sp.	Cagarras Archipelago, Brazil (4–20 m)	*Pseudomonas aeruginosa* H51	Proteobacteria	Unidentified	DOI (28 mm)	*N. gonorrhoeae* 6002	[77]	
*Petromica citrina*	Cagarras Archipelago, Brazil (4–20 m)	*Bacillus pumilus* Pc31	Firmicutes	Unidentified	DOI (28 mm)	*N. gonorrhoeae* 6002	[77]	
*Petromica citrina*	Cagarras Archipelago, Brazil (4–20 m)	*Bacillus pumilus* Pc32	Firmicutes	Unidentified	DOI (26 mm)	*N. gonorrhoeae* 6002	[77]	
*Haliclona* *occulata*	Gulf of Mannar, India (ND)	*Bacillus licheniformis* T6-1	Firmicutes	Fluorophore compound	DOI (6 mm) at 50 µM	*Salmonella typhi*	[87]	
*Dysidea granulosa*	Kavaratti Island, India (ND)	*Enterobacter* sp. TTAG	Proteobacteria	Unidentified	DOI (19 mm)	*S. typhi*	[85]	
*Callyspongia diffusa*	Southwest Coast of India (6–7 m)	*Shewanella algae* VCDB KC623651	Proteobacteria	Unidentified	DOI (11 mm)	*S. typhi*	[99]	
*Dendrilla nigra*	Southeast coast of India (ND)	*Streptomyces* sp. BTL7	Actinobacteria	Unidentified	DOI (16 mm)	*S. typhi*	[80]	
*Axinella dissimilis*	Gurraig Sound, Kilkieran Bay, Ireland (15 m)	*Pseudovibrio* Ad30	Proteobacteria	Unidentified	ND	*Salmonella typhimurium*	[78]	
*Polymastia boletiformis*, *Axinella dissimilis* and *Haliclona simulans*	Gurraig Sound, Kilkieran Bay, Ireland (15 m)	*Pseudovibrio* sp. W64, W69, W89, W74	Proteobacteria	Tropodithietic acid	DOI (≥2 mm)	*S. typhimurium*	[72]	
*Polymastia boletiformis*, *Axinella dissimilis* and *Haliclona simulans*	Gurraig Sound, Kilkieran Bay, Ireland (15 m)	*Pseudovibrio* sp. JIC5, W63, W65, W71, W99, W96, WM40, WC32, WC41, HC6	Proteobacteria	Unidentified	DOI (≥2 mm)	*S. typhimurium*	[72]	
*Polymastia boletiformis*, *Axinella dissimilis* and *Haliclona simulans*	Gurraig Sound, Kilkieran Bay, Ireland (15 m)	*Pseudovibrio* sp. W10, W62, WC43, W85, W78, W94, WM31, WM34, WC13, WC21, WC30	Proteobacteria	Unidentified	DOI (≥1 mm)	*S. typhimurium*	[72]	
*Haliclona* sp.	Cagarras Archipelago, Brazil (4–20 m)	*Pseudomonas fluorescens* H40	Proteobacteria	Unidentified	DOI (21 mm)	*Salmonella enterica*	[77]	
*Haliclona* sp.	Cagarras Archipelago, Brazil (4–20 m)	*Pseudomonas fluorescens* H41	Proteobacteria	Unidentified	DOI (17 mm)	*S. enterica*	[77]	
*Haliclona* sp.	Cagarras Archipelago, Brazil (4–20 m)	*Pseudomonas aeruginosa* H51	Proteobacteria	Unidentified	DOI (25 mm)	*S. enterica*	[77]	
*Clathrina aurea*	Cagarras Archipelago, Brazil (4–20 m)	*Pseudovibrio ascidiaceicola* Ca31	Proteobacteria	Unidentified	DOI (14 mm)	*S. enterica*	[77]	
*Paraleucilla magna*	Cagarras Archipelago, Brazil (4–20 m)	*Pseudovibrio ascidiaceicola* Pm31	Proteobacteria	Unidentified	DOI (12 mm)	*S. enterica*	[77]	
*Mycale microsigmatosa*	Cagarras Archipelago, Brazil (4–20 m)	*Pseudovibrio denitrificans* Mm37	Proteobacteria	Unidentified	DOI (14 mm)	*S. enterica*	[77]	
*Halichondria panicea*	Kiel Fjord, Baltic Sea, Germany (ND)	*Streptomyces* sp. HB202	Actinobacteria	Mayamycin	IC50 (1.16 µg/mL)	*Pseudomonas aeruginosa*	[45]	
*Haliclona* sp.	Cagarras Archipelago, Brazil (4–20 m)	*Pseudomonas fluorescens* H40, H41 and *Pseudomonas aeruginosa* H51	Proteobacteria	Diketopiperazine cyclo-(l-Leu-l-Pro)	MIC (512 µg/mL )	*P. aeruginosa*	[62]	
*Halichondria* sp.	West Coast of India (10 m)	*Bacillus licheniformis* SAB1	Firmicutes	Indole	DOI (4–6 mm)	*P. aeruginosa*	[69]	
*Halichondria* sp.	West Coast of India (10 m)	*Bacillus licheniformis* SAB1	Firmicutes	3-Phenylpropionic	DOI (4–6 mm)	*P. aeruginosa*	[69]	
*Haliclona* sp.	Cagarras Archipelago, Brazil (4–20 m)	*Pseudomonas fluorescens* H40	Proteobacteria	Unidentified	DOI (23 mm)	*P. aeruginosa*	[77]	
*Haliclona* sp.	Cagarras Archipelago, Brazil (4–20 m)	*Pseudomonas fluorescens* H41	Proteobacteria	Unidentified	DOI (20 mm)	*P. aeruginosa*	[77]	
*Petromica citrina*	Cagarras Archipelago, Brazil (4–20 m)	*Bacillus pumilus* Pc31	Firmicutes	Unidentified	DOI (35 mm)	*P. aeruginosa*	[77]	
*Petromica citrina*	Cagarras Archipelago, Brazil (4–20 m)	*Bacillus pumilus* Pc32	Firmicutes	Unidentified	DOI (35 mm)	*P. aeruginosa*	[77]	
*Haliclona* sp.	Cagarras Archipelago, Brazil (4–20 m)	*Pseudomonas aeruginosa* H51	Proteobacteria	Unidentified	DOI (30 mm)	*P. aeruginosa*	[77]	
*Clathrina aurea*	Cagarras Archipelago, Brazil (4–20 m)	*Pseudovibrio ascidiaceicola* Ca31	Proteobacteria	Unidentified	DOI (30 mm)	*P. aeruginosa*	[77]	
*Paraleucilla magna*	Cagarras Archipelago, Brazil (4–20 m)	*Pseudovibrio ascidiaceicola* Pm31	Proteobacteria	Unidentified	DOI (22 mm)	*P. aeruginosa*	[77]	
*Mycale microsigmatosa*	Cagarras Archipelago, Brazil (4–20 m)	*Pseudovibrio denitrificans* Mm37	Proteobacteria	Unidentified	DOI (30 mm)	*P. aeruginosa*	[77]	
*Axinella dissimilis*	Gurraig Sound, Kilkieran Bay, Ireland (15 m)	*Pseudovibrio* Ad30	Proteobacteria	Unidentified	ND	*P. aeruginosa*	[78]	
*Dendrilla nigra*	Southeast coast of India (ND)	*Streptomyces* sp. BTL7	Actinobacteria	Unidentified	DOI (21 mm)	*P. aeruginosa*	[80]	
*Callyspongia diffusa*	Bay of Bengal, India (10–15 m)	*Streptomyces* sp. CPI 13	Actinobacteria	Unidentified	DOI (7.7 mm)	*P. aeruginosa*	[92]	
*Callyspongia diffusa*	Bay of Bengal, India (10–15 m)	*Micromonospora* sp. CPI 12	Actinobacteria	Unidentified	DOI (6.9 mm)	*P. aeruginosa*	[92]	
*Callyspongia diffusa*	Bay of Bengal, India (10–15 m)	*Saccharomonospora* sp. CPI 9	Actinobacteria	Unidentified	DOI (6.3 mm)	*P. aeruginosa*	[92]	
*Callyspongia diffusa*	Bay of Bengal, India (10–15 m)	*Saccharomonospora* sp. CPI 3	Actinobacteria	Unidentified	DOI (6.3 mm)	*P. aeruginosa*	[92]	
*Haliclona* sp.	Cagarras Archipelago, Brazil (4–20 m)	*Pseudomonas aeruginosa* H51	Proteobacteria	Unidentified	DOI (32 mm)	*P. aeruginosa* 3AE (susceptible to atm, tzp)	[77]	
*Haliclona* sp.	Cagarras Archipelago, Brazil (4–20 m)	*Pseudomonas fluorescens* H40	Proteobacteria	Unidentified	DOI (20 mm)	*P. aeruginosa* 3AE	[77]	
*Clathrina aurea*	Cagarras Archipelago, Brazil (4–20 m)	*Pseudovibrio ascidiaceicola* Ca31	Proteobacteria	Unidentified	DOI (14 mm)	*P. aeruginosa* 3AE	[77]	
*Paraleucilla magna*	Cagarras Archipelago, Brazil (4–20 m)	*Pseudovibrio ascidiaceicola* Pm31	Proteobacteria	Unidentified	DOI (12 mm)	*P. aeruginosa* 3AE	[77]	
*Mycale microsigmatosa*	Cagarras Archipelago, Brazil (4–20 m)	*Pseudovibrio denitrificans* Mm37	Proteobacteria	Unidentified	DOI (15 mm)	*P. aeruginosa* 3AE	[77]	
*Halichondria panicea*	Kiel Fjord, Baltic Sea, Germany (ND)	*Streptomyces* sp. HB107	Actinobacteria	Unidentified	ND	*Pseudomonas fluorescens*	[44]	
*Halichondria panicea*	Kiel Fjord, Baltic Sea, Germany (ND)	*Streptomyces* sp. HB132	Actinobacteria	Unidentified	ND	*P. fluorescens*	[44]	
*Halichondria panicea*	Kiel Fjord, Baltic Sea, Germany (ND)	*Streptomyces* sp. HB202	Actinobacteria	Unidentified	ND	*P. fluorescens*	[44]	
*Halichondria panicea*	Kiel Fjord, Baltic Sea, Germany (ND)	*Streptomyces* sp. HB107	Actinobacteria	Unidentified	ND	*Pseudomonas syringae*	[44]	
*Halichondria panicea*	Kiel Fjord, Baltic Sea, Germany (ND)	*Streptomyces* sp. HB138	Actinobacteria	Unidentified	ND	*P. syringae*	[44]	
*Halichondria panicea*	Kiel Fjord, Baltic Sea, Germany (ND)	*Streptomyces* sp. HB272	Actinobacteria	Unidentified	ND	*P. syringae*	[44]	
*Halichondria panicea*	Kiel Fjord, Baltic Sea, Germany (ND)	*Streptomyces* sp. HB298	Actinobacteria	Unidentified	ND	*P. syringae*	[44]	
*Callyspongia* sp.	Kyung-Po beach, Korea (12 m)	*Brevibacterium* sp. KMD 003	Actinobacteria	6-Hydroxymethyl-1-phenazine- carboxamide	MIC (1.26 µg/mL)	*Micrococcus luteus*	[94]	
				1,6-Phenazinedimethanol	MIC (1.20 µg/mL)	*M. luteus*	[94]	
*Isodictya setifera*	Ross island, Antartica (30–40 m)	*Pseudomonas aeruginosa*	Proteobacteria	Phenazine-1-carboxylic acid and phenazine-1-carboxamide	MIC (>4.99 µg/mL)	*M. luteus*	[55]	
*Dendrilla nigra*	Southeast coast of India (ND)	*Streptomyces* sp. BTL7	Actinobacteria	Unidentified	DOI (19 mm), MIC (44 g protein/mL)	*M. luteus*	[80]	
*Callyspongia diffusa*	Bay of Bengal, India (10–15 m)	*Saccharomonospora* sp. CPI 9	Actinobacteria	Unidentified	DOI (6.6 mm)	*M. luteus*	[92]	
*Callyspongia diffusa*	Bay of Bengal, India (10–15 m)	*Saccharomonospora* sp. CPI 3	Actinobacteria	Unidentified	DOI (6.6 mm )	*M. luteus*	[92]	
unidentified	South China Sea (10 m)	*Nocardiopsis sp.* 13-33-15 and 13-12-13	Actinobacteria	1,6-Dihydroxyphenazine	DOI (18 ± 0.9 mm)	*M. luteus* SJ47	[67]	
unidentified	South China Sea (10 m)	*Nocardiopsis sp.* 13-33-15 and 13-12-13	Actinobacteria	1,6-Dimethoxyphenazine	DOI (23 ± 0.5 mm)	*M. luteus* SJ47	[67]	
*Xestospongia testudinaria*	Weizhou coral reef, China (ND)	*Aspergillus* sp.	Ascomycota	(−)-Sydonic acid	MIC (5.33 µg/mL)	*Micrococcus tetragenus*	[48]	
*Xestospongia testudinaria*	Weizhou coral reef, China (ND)	*Aspergillus* sp.	Ascomycota	(Z)-5-(Hydroxymethyl)-2-(6′-methylhept-2′-en-2′-yl)phenol	MIC (2.33 µg/mL)	*M. tetragenus*	[48]	
*Xestospongia testudinaria*	Weizhou coral reef, China (ND)	*Aspergillus* sp.	Ascomycota	Aspergiterpenoid A	MIC (2.36 µg/mL)	*M. tetragenus*	[48]	
*Xestospongia testudinaria*	Weizhou coral reef, China (ND)	*Aspergillus* sp.	Ascomycota	(−)-Sydonol	MIC (0.32 µg/mL),	*M. tetragenus*	[48]	
*Xestospongia testudinaria*	Bidong Island, Malaysia (ND)	*Serratia marcescens* IBRL USM 84	Proteobacteria	Prodigiosin	DOI (≤9 mm)	*Micrococcus* sp.	[66]	
*Petrosia ficiformis*	Paraggi, Ligurian Sea, Italy (8 m)	*Rhodococcus* sp. E1	Actinobacteria	Unidentified	ND	*Micrococcus* sp.	[86]	
*Halichondria panicea*	Kiel Fjord, Baltic Sea, Germany (ND)	*Streptomyces* sp. HB202	Actinobacteria	Mayamycin	IC50 (3.45 µg/mL)	*Brevibacterium epidermidis*	[45]	
*Halichondria panicea*	Kiel Fjord, Baltic Sea, Germany (ND)	*Streptomyces* sp. HB202	Actinobacteria	Mayamycin	IC50 (3.89 µg/mL)	*Dermabacter hominis*	[45]	
*Halichondria panicea*	Kiel Fjord, Baltic Sea, Germany (ND)	*Streptomyces* sp. HB202	Actinobacteria	Mayamycin	IC50 (14.48 µg/mL)	*Propionibacterium acnes*	[45]	
*Halichondria panicea*	Kiel Fjord, Baltic Sea, Germany (ND)	*Streptomyces* sp. HB202	Actinobacteria	Mayamycin	IC50 (13.92 µg/mL)	*Xanthomonas campestris*	[45]	
*Dysidea tupha*	Rovinj, Croatia (ND)	*Streptomyces* sp. RV15	Actinobacteria	Naphthacene glycoside SF2446A2	IC50 (2.81 ± 0.24 µg/mL)	*Chlamydia trachomatis*	[46]	
*unidentified*	ND	*Trichoderma* sp. 05FI48	Ascomycota	Trichoderin A	MIC (0.1 µg/mL)	*Mycobacterium smegmatis*	[49]	
*unidentified*	ND	*Trichoderma* sp. 05FI48	Ascomycota	Trichoderin A1	MIC (1.56 µg/mL)	*M. smegmatis*	[49]	
*unidentified*	ND	*Trichoderma* sp. 05FI48	Ascomycota	Trichoderin B	MIC (0.63 µg/mL)	*M. smegmatis*	[49]	
*unidentified*	ND	*Trichoderma* sp. 05FI48	Ascomycota	Trichoderin A	MIC (0.02 µg/mL)	*Mycobacterium bovis* BCG	[49]	
*unidentified*	ND	*Trichoderma* sp. 05FI48	Ascomycota	Trichoderin A1	MIC (0.16 µg/mL)	*M. bovis* BCG	[49]	
*unidentified*	ND	*Trichoderma* sp. 05FI48	Ascomycota	Trichoderin B	MIC (0.02 µg/mL)	*M. bovis* BCG	[49]	
*unidentified*	ND	*Trichoderma* sp. 05FI48	Ascomycota	Trichoderin A	MIC (0.12 µg /mL)	*Mycobacterium tuberculosis* H37rv	[49]	
*unidentified*	ND	*Trichoderma* sp. 05FI48	Ascomycota	Trichoderin A1	MIC (2.0 µg/mL)	*M. tuberculosis* H37rv	[49]	
*unidentified*	ND	*Trichoderma* sp. 05FI48	Ascomycota	Trichoderin B	MIC (0.13 µg/mL)	*M. tuberculosis* H37rv	[49]	
*Xestospongia testudinaria*	Weizhou coral reef, China (ND)	*Aspergillus* sp.	Ascomycota	(−)-Sydonic acid	MIC (2.66 µg/mL)	*Vibrio parahaemolyticus*	[48]	
*Asbestopluma hypogea*	La Ciotat, France (17 m)	*Streptomyces* sp. S1CA	Actinobacteria	Unidentified	ND	*V. parahaemolyticus*	[100]	
*Mycale* sp.	Gulei Port, Fujian, China (ND)	*Bacillus* sp. HNS010	Firmicutes	Unidentified	DOI (8–10 mm)	*V. parahaemolyticus*	[81]	
*Mycale* sp.	Gulei Port, Fujian, China (ND)	*Cobetia* sp. HNS023; *Nocardiopsis* HNS055; HNS058	Actinobacteria	Unidentified	DOI (8–10 mm)	*V. parahaemolyticus*	[81]	
*Mycale* sp.	Gulei Port, Fujian, China (ND)	*Streptomyces* sp. HNS054	Actinobacteria	Unidentified	DOI (10–15 mm)	*V. parahaemolyticus*	[81]	
*Phorbas tenacior*	Mediterranean Sea, Marseille, France (15 m)	*Citricoccus* sp.P1S7	Actinobacteria	Unidentified	DOI (3–6 mm)	*V. parahaemolyticus*	[101]	
*Phorbas tenacior*	Mediterranean Sea, Marseille, France (15 m)	*Pseudovibrio* sp. P1Ma4 and *Vibrio* sp. P1MaNal1	Proteobacteria	Unidentified	DOI (2–3 mm)	*V. parahaemolyticus*	[101]	
*Xestospongia testudinaria*	Weizhou coral reef, China (ND)	*Aspergillus* sp.	Ascomycota	(−)-Sydonic acid	MIC (1.33 µg/mL)	*Vibrio anguillarum*	[48]	
*Haliclona simulans*	Gurraig Sound Kilkieran Bay, Ireland (15 m)	*Bacillus subtilis* MMA7	Firmicutes	Subtilomycin	ND	*V. anguillarum*	[71]	
*Polymastia boletiformis*, *Axinella dissimilis* and *Haliclona simulans*	Gurraig Sound, Kilkieran Bay, Ireland (15 m)	*Pseudovibrio* sp. W64,	Proteobacteria	Tropodithietic acid	DOI (≥4 mm)	*V. anguillarum*	[72]	
*Polymastia boletiformis*, *Axinella dissimilis* and *Haliclona simulans*	Gurraig Sound, Kilkieran Bay, Ireland (15 m)	*Pseudovibrio* sp. W69, W89,	Proteobacteria	Tropodithietic acid	DOI (≥2 mm)	*V. anguillarum*	[72]	
*Polymastia boletiformis*, *Axinella dissimilis* and *Haliclona simulans*	Gurraig Sound, Kilkieran Bay, Ireland (15 m)	*Pseudovibrio* sp. W74	Proteobacteria	Tropodithietic acid	DOI (≥1 mm)	*V. anguillarum*	[72]	
*Polymastia boletiformis*, *Axinella dissimilis* and *Haliclona simulans*	Gurraig Sound, Kilkieran Bay, Ireland (15 m)	*Pseudovibrio* sp. JIC5, W65, W99, W85, WM31, WM34, HC6	Proteobacteria	Unidentified	DOI (≥4 mm)	*V. anguillarum*	[72]	
*Polymastia boletiformis*, *Axinella dissimilis* and *Haliclona simulans*	Gurraig Sound, Kilkieran Bay, Ireland (15 m)	*Pseudovibrio* sp. JIC6, JIC17, WM33, WC15, WC22	Proteobacteria	Unidentified	DOI (≥1 mm)	*V. anguillarum*	[72]	
*Polymastia boletiformis*, *Axinella dissimilis* and *Haliclona simulans*	Gurraig Sound, Kilkieran Bay, Ireland (15 m)	*Pseudovibrio* sp. W62, W71, WC43, W78, W94,W96, WM40, WC13, WC21, WC30, WC32, WC41, HMMA3	Proteobacteria	Unidentified	DOI (≥2 mm)	*V. anguillarum*	[72]	
*Phorbas tenacior*	Mediterranean Sea, Marseille, France (15 m)	*Citricoccus* sp.P1S7	Actinobacteria	Unidentified	DOI (3–6 mm)	*V. anguillarum*	[101]	
*Callyspongia diffusa*	Southwest Coast of India (6–7 m)	*Shewanella algae* VCDB KC623651	Proteobacteria	Unidentified	DOI (10 mm)	*Vibrio anguillarum*	[99]	
*Dendrilla nigra*	Southeast coast of India (ND)	*Streptomyces* sp. BTL7	Actinobacteria	Unidentified	DOI (15 mm), MIC (176 g protein/mL)	*Vibrio fisheri*	[80]	
*Phorbas tenacior*	Mediterranean Sea, Marseille, France (15 m)	*Citricoccus* sp.P1S7	Actinobacteria	Unidentified	DOI (3–6 mm)	*Vibrio algynoliticus*	[101]	
*Dysidea herbacea*	Koror, Republic Palau (1 m)	*Oscillatoria spongeliae*	Cyanobacteria	2-(2′,4′-Dibromophenyl)-4,6-dibromophenol	ND	*Vibrio harveyi*,	[64]	
*Halichondria* sp.	West Coast of India (10 m)	*Bacillus licheniformis* SAB1	Firmicutes	4,4′-Oxybis(3-phenylpropionic acid)	DOI (4–6 mm)	*Vibrio cholerae*	[69]	
*Mycale* sp.	Gulei Port, Fujian, China (ND)	*Vibrio* sp. HNS022, HNS029; *Streptomyces* sp. HNS049, HNS054, HNS056; *Nocardiopsis* sp. HNS055	Proteobacteria	Unidentified	DOI (8–10 mm)	*Vibrio diabolicus*	[81]	
*Callyspongia diffusa*	Southwest Coast of India (6–7 m)	*Shewanella algae* VCDB KC623651	Proteobacteria	Unidentified	DOI (14 mm)	*Vibrio fluvialis*	[99]	
*Asbestopluma hypogea*	La Ciotat, France (17 m)	*Streptomyces* sp. S1CA	Actinobacteria	Unidentified	ND	*Vibrio* sp*.* S2SW	[100]	
*Asbestopluma hypogea*	La Ciotat, France (17 m)	*Streptomyces* sp. S1CA	Actinobacteria	Unidentified	ND	*Vibrio* sp. S3SW	[100]	
*Xestospongia testudinaria*	Bidong Island, Malaysia (ND)	*Serratia marcescens* IBRL USM 84	Proteobacteria	Prodigiosin	DOI (10-14 mm)	*Agrobacterium tumefaciens*	[66]	
*Aplysina aerophoba*	Banyuls-sur-Mer, France (5–15 m)	*Bacillus subtilis* A184	Firmicutes	Surfactin iturin fengycin	ND	*tumefaciens*	[68]	
*Hymeniacidon perleve*	Nanji Island, China (ND)	*Pseudomonas* sp. NJ6-3-1	Proteobacteria	Unidentified	DOI (3–5 mm)	*tumefaciens*	[74]	
*Xestospongia testudinaria*	Bidong Island, Malaysia (ND)	*Serratia marcescens* IBRL USM 84	Proteobacteria	Prodigiosin	DOI (≤9 mm)	*Acinetobacter anitratus*	[66]	
*Haliclona* sp.	Cagarras Archipelago, Brazil (4–20 m)	*Pseudomonas fluorescens* H40	Proteobacteria	Unidentified	DOI (20 mm)	*baumanii*	[77]	
*Haliclona* sp.	Cagarras Archipelago, Brazil (4–20 m)	*Pseudomonas fluorescens* H41	Proteobacteria	Unidentified	DOI (20 mm)	*baumanii*	[77]	
*Haliclona* sp.	Cagarras Archipelago, Brazil (4–20 m)	*Pseudomonas fluorescens* H40	Proteobacteria	Unidentified	DOI (19 mm)	*Acinetobacter calcoaceticus*	[77]	
*Haliclona* sp.	Cagarras Archipelago, Brazil (4–20 m)	*Pseudomonas fluorescens* H41	Proteobacteria	Unidentified	DOI (18 mm)	*calcoaceticus*	[77]	
*Haliclona* sp.	Cagarras Archipelago, Brazil (4–20 m)	*Pseudomonas aeruginosa* H51	Proteobacteria	Unidentified	DOI (30 mm)	*calcoaceticus*	[77]	
*Petromica citrina*	Cagarras Archipelago, Brazil (4–20 m)	*Bacillus pumilus* Pc31	Firmicutes	Unidentified	DOI (35 mm)	*calcoaceticus*	[77]	
*Petromica citrina*	Cagarras Archipelago, Brazil (4–20 m)	*Bacillus pumilus* Pc32	Firmicutes	Unidentified	DOI (30 mm)	*calcoaceticus*	[77]	
*Clathrina aurea*	Cagarras Archipelago, Brazil (4–20 m)	*Pseudovibrio ascidiaceicola* Ca31	Proteobacteria	Unidentified	DOI (18 mm)	*calcoaceticus*	[77]	
*Paraleucilla magna*	Cagarras Archipelago, Brazil (4–20 m)	*Pseudovibrio ascidiaceicola* Pm31	Proteobacteria	Unidentified	DOI (23 mm)	*calcoaceticus*	[77]	
*Mycale microsigmatosa*	Cagarras Archipelago, Brazil (4–20 m)	*Pseudovibrio denitrificans* Mm37	Firmicutes	Unidentified	DOI (23 mm)	*calcoaceticus*	[77]	
*Petromica citrina*	Cagarras Archipelago, Brazil (4–20 m)	*Bacillus pumilus* Pc31	Firmicutes	Unidentified	DOI (45 mm)	*Acinetobacter* sp	[77]	
*Petromica citrina*	Cagarras Archipelago, Brazil (4–20 m)	*Bacillus pumilus* Pc32	Firmicutes	Unidentified	DOI (45 mm)	*Acinetobacter* sp	[77]	
*Halichondria* sp.	West Coast of India (10 m)	*Bacillus licheniformis* SAB1	Firmicutes	Indole	DOI (1–3 mm)	*Acinetobacter* sp.	[69]	
*Halichondria* sp.	West Coast of India (10 m)	*Bacillus licheniformis* SAB1	Firmicutes	3-Phenylpropionic	DOI (4–6 mm)	*Acinetobacter* sp	[69]	
*Xestospongia testudinaria*	Bidong Island, Malaysia (ND)	*Serratia marcescens* IBRL USM 84	Proteobacteria	Prodigiosin	DOI (≤9 mm)	*Erwinia* sp	[66]	
*Aplysina aerophoba*	Banyuls-sur-Mer, France (5–15 m)	*Bacillus subtilis* A184	Firmicutes	Surfactin Iturin Fengycin	ND	*Clavibacter michiganensis*	[68]	
*Aplysina aerophoba*	Banyuls-sur-Mer, France (5–15 m)	*Bacillus subtilis* A190	Firmicutes	Surfactin	ND	*Clavibacter michiganensis*	[68]	
*Aplysina aerophoba*	Banyuls-sur-Mer, France (5–15 m)	*Bacillus subtilis* A202	Firmicutes	Iturin	ND	*Clavibacter michiganensis*	[68]	
*Aplysina aerophoba*	Banyuls-sur-Mer, France (5–15 m)	*Bacillus subtilis* A184	Firmicutes	Surfactin Iturin Fengycin	ND	*Proteus vulgaris*	[68]	
*Aplysina aerophoba*	Banyuls-sur-Mer, France (5–15 m)	*Bacillus subtilis* A190	Firmicutes	Surfactin	ND	*Proteus vulgaris*	[68]	
*Aplysina aerophoba*	Banyuls-sur-Mer, France (5–15 m)	*Bacillus subtilis* A202	Firmicutes	Iturin	ND	*Proteus vulgaris*	[68]	
*Callyspongia diffusa*	Southwest Coast of India (6–7 m)	*Shewanella algae* VCDB KC623651	Proteobacteria	Unidentified	DOI (10 mm)	*Proteus vulgaris*	[99]	
*Callyspongia diffusa*	Bay of Bengal, India (10–15 m)	*Micromonospora* sp. CPI 12	Actinobacteria	Unidentified	DOI (8 mm)	*Proteus mirabilis*	[92]	
*Callyspongia diffusa*	Bay of Bengal, India (10–15 m)	*Saccharomonospora* sp. CPI 9	Actinobacteria	Unidentified	DOI (6 mm	*Proteus mirabilis*	[92]	
*Callyspongia diffusa*	Bay of Bengal, India (10–15 m)	*Saccharomonospora* sp. CPI 3	Actinobacteria	Unidentified	DOI (6 mm)	*Proteus mirabilis*	[92]	
*Dysidea avara*	Mediterranean sea (ND)	*Actinokinespora* sp. EG49	Actinobacteria	1,6-Dihydroxyphenazine (result of co-culture)	DOI (15 mm)	*Actinokinespora* sp. EG49	[98]	
*Spheciospongia vagabunda*	Red Sea (ND)	*Nocardiopsis* sp. RV163	Actinobacteria					
*Haliclona simulans*	Gurraig Sound Kilkieran Bay, Ireland (15 m)	*Bacillus subtilis* MMA7	Firmicutes	Subtilomycin	ND	*Listeria monocytogenes*	[71]	
*Axinella dissimilis*	Gurraig Sound, Kilkieran Bay, Ireland (15 m)	*Pseudovibrio* Ad30	Proteobacteria	Unidentified	ND	*Listeria monocytogenes*	[78]	
*Haliclona simulans*	Gurraig Sound, Kilkieran Bay, Ireland (15 m)	Streptomyces sp. SM2 and SM4	Actinobacteria	Unidentified	ND	*Listeria monocytogenes*	[88]	
*Haliclona simulans*	Gurraig Sound Kilkieran Bay, Ireland (15 m)	*Bacillus subtilis* MMA7	Firmicutes	Subtilomycin	ND	*Listeria innocua*	[71]	
*Haliclona simulans*	Gurraig Sound Kilkieran Bay, Ireland (15 m)	*Bacillus subtilis* MMA7	Firmicutes	Subtilomycin	ND	*Clostridium sporogenes*	[71]	
*Axinella dissimilis*	Gurraig Sound, Kilkieran Bay, Ireland (15 m)	*Pseudovibrio* Ad30	Proteobacteria	Unidentified	ND	*Clostridium perfringens*	[78]	
*Axinella dissimilis*	Gurraig Sound, Kilkieran Bay, Ireland (15 m)	*Pseudovibrio* Ad30	Proteobacteria	Unidentified	ND	*Clostridium difficile*	[78]	
*Dendrilla nigra*	Southeast coast of India (15 m)	*Streptomyces* sp. BTL7	Actinobacteria	Unidentified	DOI (10 mm)	*Clostridium botulinum*	[80]	
*Haliclona simulans*	Gurraig Sound, Kilkieran Bay, Ireland (15 m)	*Streptomyces* sp. SM2 and SM4	Actinobacteria	Unidentified	ND	*Clostridium difficile*	[88]	
*Haliclona simulans*	Gurraig Sound Kilkieran Bay, Ireland (15 m)	*Bacillus subtilis* MMA7	Firmicutes	Subtilomycin	ND	*Lactobacillus lactis*	[71]	
*Callyspongia diffusa*	Southwest Coast of India (6–7 m)	*Shewanella algae* VCDB KC623651	Firmicutes	Unidentified	DOI (10 mm)	*L. lactis*	[99]	
*Haliclona simulans*	Gurraig Sound Kilkieran Bay, Ireland (15 m)	*Bacillus subtilis* MMA7	Firmicutes	Subtilomycin	ND	*Aeromonas hydrophila*	[71]	
*Haliclona simulans*	Gurraig Sound Kilkieran Bay, Ireland (15 m)	*Bacillus subtilis* MMA7	Firmicutes	Subtilomycin	ND	*Alteromonas* sp.	[71]	
*Polymastia boletiformis*, *Axinella dissimilis* and *Haliclona simulans*	Gurraig Sound, Kilkieran Bay, Ireland (15 m)	*Pseudovibrio* sp. W64, W69, W89, W74	Proteobacteria	Tropodithietic acid	DOI (≥4 mm)	*Yersinia ruckerri*	[72]	
*Polymastia boletiformis*, *Axinella dissimilis* and *Haliclona simulans*	Gurraig Sound, Kilkieran Bay, Ireland (15 m)	*Pseudovibrio* sp. JIC5, JIC17, W10, W62, W63, W65, W71, W99, W85, W96, WM31, WM34, WM40, WC13, WC22, WC30, WC32, WC41, HC6	Proteobacteria	Unidentified	DOI (≥4 mm)	*Y. ruckerri*	[72]	
*Polymastia boletiformis*,* Axinella dissimilis and Haliclona simulans*	Gurraig Sound, Kilkieran Bay, Ireland (15 m)	*Pseudovibrio* sp. WC43, W78, W94,WM33, WC21, HMMA3	Proteobacteria	Unidentified	DOI (≥4 mm)	*Y. ruckerri*	[72]	
*Polymastia boletiformis*,* Axinella dissimilis and Haliclona simulans*	Gurraig Sound, Kilkieran Bay, Ireland (15 m)	*Pseudovibrio* sp. JIC6, WC15	Proteobacteria	Unidentified	DOI (≥1 mm)	*Y. ruckerri*	[72]	
*Polymastia boletiformis*, *Axinella dissimilis* and *Haliclona simulans*	Gurraig Sound, Kilkieran Bay, Ireland (15 m)	*Pseudovibrio* sp. W64, W69, W89	Proteobacteria	Tropodithietic acid	DOI (≥4 mm)	*Edwardsialla tarda*	[72]	
*Polymastia boletiformis*, *Axinella dissimilis* and *Haliclona simulans*	Gurraig Sound, Kilkieran Bay, Ireland (15 m)	*Pseudovibrio* sp. W74	Proteobacteria	Tropodithietic acid	DOI (≥2 mm)	*E. tarda*	[72]	
*Polymastia boletiformis*, *Axinella dissimilis* and *Haliclona simulans*	Gurraig Sound, Kilkieran Bay, Ireland (15 m)	*Pseudovibrio* sp. JIC5, W63, W99	Proteobacteria	Unidentified	DOI (≥4 mm)	*E. tarda*	[72]	
*Polymastia boletiformis*, *Axinella dissimilis* and *Haliclona simulans*	Gurraig Sound, Kilkieran Bay, Ireland (15 m)	*Pseudovibrio* sp. JIC6, JIC17, W10, W62, W65, W71, W85, W96, WM31,WM34, WM40, WC13, WC32, WC41, HC6	Proteobacteria	Unidentified	DOI (≥2 mm)	*E. tarda*	[72]	
*Polymastia boletiformis*, *Axinella dissimilis* and *Haliclona simulans*	Gurraig Sound, Kilkieran Bay, Ireland (15 m)	*Pseudovibrio* sp. W78, W94, WM33, WC21, WC22, WC30, HMMA3	Proteobacteria	Unidentified	DOI (≥1 mm)	*E. tarda*	[72]	
*Polymastia boletiformis*, *Axinella dissimilis* and *Haliclona simulans*	Gurraig Sound, Kilkieran Bay, Ireland (15 m)	*Pseudovibrio* sp. W64, W74	Proteobacteria	Tropodithietic acid	DOI (≥4 mm)	*Morganella morganii*	[72]	
*Polymastia boletiformis*, *Axinella dissimilis* and *Haliclona simulans*	Gurraig Sound, Kilkieran Bay, Ireland (15 m)	*Pseudovibrio* sp. W69, W89,	Proteobacteria	Tropodithietic acid	DOI (≥ 2 mm)	*M. morganii*	[72]	
*Polymastia boletiformis*, *Axinella dissimilis* and *Haliclona simulans*	Gurraig Sound, Kilkieran Bay, Ireland (15 m)	*Pseudovibrio* sp. JIC5, W62, W65, W71, W99, W78, WM34, HC6	Proteobacteria	Unidentified	DOI (≥4 mm)	*M. morganii*	[72]	
*Polymastia boletiformis*, *Axinella dissimilis* and *Haliclona simulans*	Gurraig Sound, Kilkieran Bay, Ireland (15 m)	*Pseudovibrio* sp. JIC17, W94, W96, WM40, WC13, WC21, WC32, WC41	Proteobacteria	Unidentified	DOI (≥2 mm)	*M. morganii*	[72]	
*Polymastia boletiformis*, *Axinella dissimilis* and *Haliclona simulans*	Gurraig Sound, Kilkieran Bay, Ireland (15 m)	*Pseudovibrio* sp. JIC6, W10, WC43, W85, WM31, WC15, WC22,WC30, HMMA3	Proteobacteria	Unidentified	DOI (≥1 mm)	*M. morganii*	[72]	
*Polymastia boletiformis*, *Axinella dissimilis* and *Haliclona simulans*	Gurraig Sound, Kilkieran Bay, Ireland (15 m)	*Pseudovibrio* sp. W64, W69, W89	Proteobacteria	Tropodithietic acid	DOI (≥2 mm)	*Pandoraea sputorum*	[72]	
*Polymastia boletiformis*, *Axinella dissimilis* and *Haliclona simulans*	Gurraig Sound, Kilkieran Bay, Ireland (15 m)	*Pseudovibrio* sp. W74	Proteobacteria	Tropodithietic acid	DOI (≥1 mm)	*P. sputorum*	[72]	
*Polymastia boletiformis*, *Axinella dissimilis* and *Haliclona simulans*	Gurraig Sound, Kilkieran Bay, Ireland (15 m)	*Pseudovibrio* sp. W63	Proteobacteria	Unidentified	DOI (4 mm)	*P. sputorum*	[72]	
*Polymastia boletiformis*, *Axinella dissimilis* and *Haliclona simulans*	Gurraig Sound, Kilkieran Bay, Ireland (15 m)	*Pseudovibrio* sp. W62, WM40	Proteobacteria	Unidentified	DOI (≥2 mm)	*P. sputorum*	[72]	
*Polymastia boletiformis*, *Axinella dissimilis* and *Haliclona simulans*	Gurraig Sound, Kilkieran Bay, Ireland (15 m)	*Pseudovibrio* sp. JIC5, JIC6, JIC17, W10, W65, W71, W99, WC43, W85, W78, W96, WM34, WC32, HC6	Proteobacteria	Unidentified	DOI (≥1 mm)	*P. sputorum*	[72]	
*Haliclona* sp.	Cagarras Archipelago, Brazil (4–20 m)	*Pseudomonas fluorescens* H40	Proteobacteria	Unidentified	DOI (23 mm)	*Corynebacterium fimi*	[77]	
*Haliclona* sp.	Cagarras Archipelago, Brazil (4–20 m)	*Pseudomonas fluorescens* H41	Proteobacteria	Unidentified	DOI (26 mm)	*Corynebacterium fimi*	[77]	
*Haliclona* sp.	Cagarras Archipelago, Brazil (4–20 m)	*Pseudomonas aeruginosa* H51	Proteobacteria	Unidentified	DOI (43 mm)	*Corynebacterium fimi*	[77]	
*Haliclona sp.*	Cagarras Archipelago, Brazil (4–20 m)	*Virgibacillus pantothenticus* H31	Firmicutes	Unidentified	DOI (17 mm)	*Corynebacterium fimi*	[77]	
*Haliclona sp.*	Cagarras Archipelago, Brazil (4–20 m)	*Bacillus flexus* H42	Firmicutes	Unidentified	DOI (21 mm)	*Corynebacterium fimi*	[77]	
*Dragmacidon reticulatus*	Cagarras Archipelago, Brazil (4–20 m)	*Bacillus pumilus* Dr31	Firmicutes	Unidentified	DOI (20 mm)	*Corynebacterium fimi*	[77]	
*Petromica citrina*	Cagarras Archipelago, Brazil (4–20 m)	*Bacillus pumilus* Pc31	Firmicutes	Unidentified	DOI (46 mm)	*Corynebacterium fimi*	[77]	
*Petromica citrina*	Cagarras Archipelago, Brazil (4–20 m)	*Bacillus pumilus* Pc32	Firmicutes	Unidentified	DOI (42 mm)	*Corynebacterium fimi*	[77]	
*Clathrina aurea*	Cagarras Archipelago, Brazil (4–20 m)	*Pseudovibrio ascidiaceicola* Ca31	Proteobacteria	Unidentified	DOI (31 mm)	*Corynebacterium fimi*	[77]	
*Paraleucilla magna*	Cagarras Archipelago, Brazil (4–20 m)	*Pseudovibrio ascidiaceicola* Pm31	Proteobacteria	Unidentified	DOI (24 mm)	*Corynebacterium fimi*	[77]	
*Paraleucilla magna*	Cagarras Archipelago, Brazil (4–20 m)	*Pseudovibrio ascidiaceicola* Pm52	Proteobacteria	Unidentified	DOI (15 mm)	*Corynebacterium fimi*	[77]	
*Mycale microsigmatosa*	Cagarras Archipelago, Brazil (4–20 m)	*Pseudovibrio denitrificans* Mm37	Proteobacteria	Unidentified	DOI (34 mm)	*Corynebacterium fimi*	[77]	
*Haliclona* sp.	Cagarras Archipelago, Brazil (4–20 m)	*Pseudomonas fluorescens* H40	Proteobacteria	Unidentified	DOI (18 mm)	*Serratia marcescens*	[77]	
*Haliclona* sp.	Cagarras Archipelago, Brazil (4–20 m)	*Pseudomonas fluorescens* H41	Proteobacteria	Unidentified	DOI (17 mm)	*S. marcescens*	[77]	
*Haliclona* sp.	Cagarras Archipelago, Brazil (4–20 m)	*Pseudomonas aeruginosa* H51	Proteobacteria	Unidentified	DOI (20 mm)	*S. marcescens*	[77]	
*Clathrina aurea*	Cagarras Archipelago, Brazil (4–20 m)	*Pseudovibrio ascidiaceicola* Ca31	Proteobacteria	Unidentified	DOI (13 mm)	*Stenotrophomonas maltophilia*	[77]	
*Paraleucilla magna*	Cagarras Archipelago, Brazil (4–20 m)	*Pseudovibrio ascidiaceicola* Pm31	Proteobacteria	Unidentified	DOI (13 mm)	*S. maltophilia*	[77]	
*Mycale microsigmatosa*	Cagarras Archipelago, Brazil (4–20 m)	*Pseudovibrio denitrificans* Mm37	Proteobacteria	Unidentified	DOI (15 mm)	*S. maltophilia*	[77]	
*Haliclona* sp.	Cagarras Archipelago, Brazil (4–20 m)	*Pseudomonas fluorescens* H40	Proteobacteria	Unidentified	DOI (19 mm)	*Citrobacter freundii*	[77]	
*Haliclona* sp.	Cagarras Archipelago, Brazil (4–20 m)	*Pseudomonas fluorescens* H41	Proteobacteria	Unidentified	DOI (16 mm)	*C. freundii*	[77]	
*Haliclona* sp.	Cagarras Archipelago, Brazil (4–20 m)	*Pseudomonas aeruginosa* H51	Proteobacteria	Unidentified	DOI (26 mm)	*C. freundii*	[77]	
*Paraleucilla magna*	Cagarras Archipelago, Brazil (4–20 m)	*Pseudovibrio ascidiaceicola* Pm31	Proteobacteria	Unidentified	DOI (10 mm)	*C. freundii*	[77]	
*Xestospongia testudinaria*	Weizhou coral reef, China (ND)	*Aspergillus* sp.	Ascomycota	(−)-Sydonic acid	MIC (0.66 µg/mL)	*Sarcina lutea*	[48]	
*Dysidea herbacea*	Koror, Republic Palau (1 m)	*Oscillatoria spongeliae*	Cyanobacteria	2-(2′,4′-Dibromophenyl)-4,6-dibromophenol	ND	*Synechococcus* sp.	[64]	
*Asbestopluma hypogea*	La Ciotat, France (17 m)	*Streptomyces* sp. S1CA	Actinobacteria	Unidentified	ND	*Ruegeria* sp. S13SW	[100]	
*Asbestopluma hypogea*	La Ciotat, France (17 m)	*Streptomyces* sp. S1CA	Actinobacteria	Unidentified	ND	*Sulfitobacter* sp. S16SW	[100]	
*Asbestopluma hypogea*	La Ciotat, France (17 m)	*Streptomyces* sp. S1CA	Actinobacteria	Unidentified	ND	*Pseudoalteromonas distincta*	[100]	
*Phorbas tenacior*	Mediterranean Sea, Marseille, France (15 m)	*Citricoccus* sp.P1S7	Actinobacteria	Unidentified	3–6 mm	*P. distincta*	[101]	
*Phorbas tenacior*	Mediterranean Sea, Marseille, France (15 m)	*Pseudovibrio* sp. P1Ma4 and *Vibrio* sp. P1MaNal1	Proteobacteria	Unidentified	2–3 mm	*P. distincta*	[101]	
*Dendrilla nigra*	Vizhinjam coast, India (10–15 m)	*Streptomyces* sp. MSI051	Actinobacteria	Unidentified	MIC (32 ± 0.61 µg protein/mL)	unidentified biofilm bacterium EB1	[73]	
*Dendrilla nigra*	Vizhinjam coast, India (10–15 m)	*Streptomyces* sp. MSI051	Actinobacteria	Unidentified	MIC (34 ± 2.18 µg protein/mL)	unidentified biofilm bacterium EB4	[73]	
*Halichondria panicea*	Kiel Fjord, Baltic Sea, Germany (ND)	*Streptomyces* sp. HB107	Actinobacteria	Unidentified	ND	*Xanthomonas campestris*	[44]	
*Halichondria panicea*	Kiel Fjord, Baltic Sea, Germany (ND)	*Streptomyces* sp. HB132	Actinobacteria	Unidentified	ND	*X. campestris*	[44]	
*Halichondria panicea*	Kiel Fjord, Baltic Sea, Germany (ND)	*Streptomyces* sp. HB138	Actinobacteria	Unidentified	ND	*X. campestris*	[44]	
*Halichondria panicea*	Kiel Fjord, Baltic Sea, Germany (ND)	*Streptomyces* sp. HB202	Actinobacteria	Unidentified	ND	*X. campestris*	[44]	
*Halichondria panicea*	Kiel Fjord, Baltic Sea, Germany (ND)	*Streptomyces* sp. HB253	Actinobacteria	Unidentified	ND	*X. campestris*	[44]	
*Halichondria panicea*	Kiel Fjord, Baltic Sea, Germany (ND)	*Streptomyces* sp. HB291	Actinobacteria	Unidentified	ND	*X. campestris*	[44]	
*Halichondria panicea*	Kiel Fjord, Baltic Sea, Germany (ND)	*Streptomyces* sp. HB298	Actinobacteria	Unidentified	ND	*X. campestris*	[44]	
*Halichondria panicea*	Kiel Fjord, Baltic Sea, Germany (ND)	*Streptomyces* sp. HB132	Actinobacteria	Unidentified	ND	*Erwinia amylovora*	[44]	
*Halichondria panicea*	Kiel Fjord, Baltic Sea, Germany (ND)	*Streptomyces* sp. HB202	Actinobacteria	Unidentified	ND	*E. amylovora*	[44]	
*Halichondria panicea*	Kiel Fjord, Baltic Sea, Germany (ND)	*Streptomyces* sp. HB320	Actinobacteria	Unidentified	ND	*E. amylovora*	[44]	
*Halichondria panicea*	Kiel Fjord, Baltic Sea, Germany (ND)	*Streptomyces* sp. HB328	Actinobacteria	Unidentified	ND	*E. amylovora*	[44]	
*Halichondria panicea*	Kiel Fjord, Baltic Sea, Germany (ND)	*Streptomyces* sp. HB100	Actinobacteria	Unidentified	ND	*Ralstonia solanacearum*	[44]	
*Halichondria panicea*	Kiel Fjord, Baltic Sea, Germany (ND)	*Streptomyces* sp. HB107	Actinobacteria	Unidentified	ND	*R. solanacearum*	[44]	
*Halichondria panicea*	Kiel Fjord, Baltic Sea, Germany (ND)	*Streptomyces* sp. HB117	Actinobacteria	Unidentified	ND	*R. solanacearum*	[44]	
*Halichondria panicea*	Kiel Fjord, Baltic Sea, Germany (ND)	*Streptomyces* sp. HB142	Actinobacteria	Unidentified	ND	*R. solanacearum*	[44]	
*Halichondria panicea*	Kiel Fjord, Baltic Sea, Germany (ND)	*Streptomyces* sp. HB156	Actinobacteria	Unidentified	ND	*R. solanacearum*	[44]	
*Halichondria panicea*	Kiel Fjord, Baltic Sea, Germany (ND)	*Streptomyces* sp. HB238	Actinobacteria	Unidentified	ND	*R. solanacearum*	[44]	
*Halichondria panicea*	Kiel Fjord, Baltic Sea, Germany (ND)	*Streptomyces* sp. HB253	Actinobacteria	Unidentified	ND	*R. solanacearum*	[44]	
*Halichondria panicea*	Kiel Fjord, Baltic Sea, Germany (ND)	*Streptomyces* sp. HB254	Actinobacteria	Unidentified	ND	*R. solanacearum*	[44]	
*Halichondria panicea*	Kiel Fjord, Baltic Sea, Germany (ND)	*Streptomyces* sp. HB272	Actinobacteria	Unidentified	ND	*R. solanacearum*	[44]	
*Halichondria panicea*	Kiel Fjord, Baltic Sea, Germany (ND)	*Streptomyces* sp. HB274	Actinobacteria	Unidentified	ND	*R. solanacearum*	[44]	
*Halichondria panicea*	Kiel Fjord, Baltic Sea, Germany (ND)	*Streptomyces* sp. HB375	Actinobacteria	Unidentified	ND	*R. solanacearum*	[44]	
*Pseudoceratina clavata*	Heron Island, Great Barrier Reef (14 m)	*Salinispora* sp. M101	Actinobacteria	Unidentified	DOI (>5 mm)	unidentified marine bacterial isolate SW09 from sponge *P.* *clavata* (high G + C Gram-positive)	[79]	
*Pseudoceratina clavata*	Heron Island, Great Barrier Reef (14 m)	*Salinispora* sp. M102, M403, M413	Actinobacteria	Unidentified	DOI (>5 mm)	unidentified marine bacterial isolate SW09	[79]	
*Pseudoceratina clavata*	Heron Island, Great Barrier Reef (14 m)	*Salinispora* sp. M412	Actinobacteria	Unidentified	DOI (>5 mm)	unidentified marine bacterial isolate SW09	[79]	
*Pseudoceratina clavata*	Heron Island, Great Barrier Reef (14 m)	*Salinispora* sp. M414, SW10, SW 15 and SW 17	Actinobacteria	Unidentified	DOI (>5 mm)	unidentified marine bacterial isolate SW09	[79]	
*Pseudoceratina clavata*	Heron Island, Great Barrier Reef (14 m)	*Salinispora* sp. SW02	Actinobacteria	Unidentified	DOI (>5 mm)	unidentified marine bacterial isolate SW09	[79]	
*Pseudoceratina clavata*	Heron Island, Great Barrier Reef (14 m)	*Salinispora* sp. M101	Actinobacteria	Unidentified	DOI (>5 mm)	unidentified marine bacterial isolate DE06 from sponge *P. clavata*: (low G + C Gram-positive)	[79]	
*Pseudoceratina clavata*	Heron Island, Great Barrier Reef (14 m)	*Salinispora* sp. M102, M403, M413	Actinobacteria	Unidentified	DOI (>5 mm)	unidentified marine bacterial isolate DE06	[79]	
*Pseudoceratina clavata*	Heron Island, Great Barrier Reef (14 m)	*Salinispora* sp. M412	Actinobacteria	Unidentified	DOI (>5 mm)	unidentified marine bacterial isolate DE06	[79]	
*Pseudoceratina clavata*	Heron Island, Great Barrier Reef (14 m)	*Salinispora* sp. SW02	Actinobacteria	Unidentified	DOI (>5 mm)	unidentified marine bacterial isolate DE06	[79]	
*Pseudoceratina clavata*	Heron Island, Great Barrier Reef (14 m)	*Salinispora sp. M414*,* SW10*,* SW 15 and SW 17*	Actinobacteria		DOI (>5 mm)	unidentified marine bacterial isolate DE06	[79]	
*Pseudoceratina clavata*	Heron Island, Great Barrier Reef (14 m)	*Salinispora* sp. M101	Actinobacteria	Unidentified	DOI (>5 mm)	unidentified bacterial isolate DE05 from sponge *P.* *clavata* (γ-proteobacteria)	[79]	
*Pseudoceratina clavata*	Heron Island, Great Barrier Reef (14 m)	*Salinispora* sp. M102, M403, M413	Actinobacteria	Unidentified	DOI (>5 mm)	unidentified bacterial isolate DE05 (γ-proteobacteria)	[79]	
*Pseudoceratina clavata*	Heron Island, Great Barrier Reef (14 m)	*Salinispora* sp. M412	Actinobacteria	Unidentified	DOI (>5 mm)	unidentified bacterial isolate DE05 (γ-proteobacteria)	[79]	
*Pseudoceratina clavata*	Heron Island, Great Barrier Reef (14 m)	*Salinispora* sp. M414, SW10, SW 15 and SW 17	Actinobacteria	Unidentified	DOI (>5 mm)	Unidentified bacterial isolate DE05 (γ-proteobacteria)	[79]	
*Pseudoceratina clavata*	Heron Island, Great Barrier Reef (14 m)	*Salinispora* sp. SW02	Actinobacteria	Unidentified	DOI (>5 mm)	Unidentified bacterial isolate DE05 (γ-proteobacteria)	[79]	

Table 2 is organised according to the target bacteria. IC_50_: half maximum inhibitory concentration; MIC: minimum inhibitory concentration; DOI: diameter of inhibition; ND: not determined. Susceptible to [77]: amp = ampicillin; atm = aztreonam; azm = azithromycin; caz = ceftazidimine; cef = cefalotin; chl = chloramphenicol; cip = ciprofloxacin; cpd = cefpodoxime; fox = cefoxitin; gen = gentamicin; oxa = oxacillin; pen = penicillin; sxt = trimethoprim/sulfamethoxazole; tet = tetracycline; tzp = piperacillin/tazobactam; van = vancomycin.

**Table 3:** Bioactive compounds with antifungal activity from sponge-associated microbes.

Sponge	Origin (Depth)	Microorganism	Phylum	Compound	Property	Target	Reference
*Aplysina fistularis*	Sharm El-Sheikh, Egypt (ND)	*Streptomyces* sp. Hedaya48	Actinobacteria	Saadamycin	MIC (2.22 µg/mL)	*Candida albicans*	[107]
*Aplysina fistularis*	Sharm El-Sheikh, Egypt (ND)	*Streptomyces* sp. Hedaya48	Actinobacteria	5,7-Dimethoxy-4-*p*-methoxylphenylcoumarin	MIC (15 µg/mL)	*C. albicans*	[107]
*Halichondria japonica*	Iriomote island, Japan (ND)	*Phoma* sp. Q60596	Ascomycota	YM-202204	IC_80_ (6.25 µg/mL)	*C. albicans*	[108]
*Haliclona simulans*	Gurraig Sound Kilkieran Bay, Ireland (15 m)	*Streptomyces* sp. SM8	Actinobacteria	Mixture of kitamycin A or B, and antimycin A3 or A7	MIC (240 µg/mL)	*C. albicans*	[95]
*Haliclona simulans*	Gurraig Sound Kilkieran Bay, Ireland (15 m)	*Streptomyces* sp. SM8	Actinobacteria	Antimycin A2, A8, A11, or A17	MIC (210 µg/mL)	*C. albicans*	[95]
*Haliclona simulans*	Gurraig Sound Kilkieran Bay, Ireland (15 m)	*Streptomyces* sp. SM8	Actinobacteria	Antimycin A3 or A7	MIC (80 µg/mL)	*C. albicans*	[95]
*Haliclona simulans*	Gurraig Sound Kilkieran Bay, Ireland (15 m)	*Streptomyces* sp. SM8	Actinobacteria	Antimycin A2, A8, A11, or A17, antimycin A3 or A7	MIC (90 µg/mL)	*C. albicans*	[95]
*Halichondria* sp.	West Coast of India (10 m)	*Bacillus* sp. SAB1	Firmicutes	3-Phenylpropionic acid	DOI (7–10 mm) at 50µg/disk	*C. albicans*	[69]
*Halichondria* sp.	West Coast of India (10 m)	*Bacillus* sp. SAB1	Firmicutes	4,4′-Oxybis(3-phenylpropionic acid)	DOI (4–6 mm) at 50µg/disk	*C. albicans*	[69]
*Xestospongia exigua*	Bali Sea, Indonesia (ND)	*Penicillium* *cf.* montanense	Ascomycota	Xestodecalactone B	MIC (28.03 µg/disk)	*C. albicans*	[111]
unidentified	Iriomote island, Japan (ND)	*Streptomyces* sp. Ni-80	Actinobacteria	Urauchimycins A and B	MIC (10 µg/mL)	*C. albicans*	[112]
*Haliclona* sp.	Tateyama, Japan (ND)	*Streptomyces bambergiensis*	Actinobacteria	Unidentified	DOI (5 mm)	*C. albicans*	[113]
*Haliclona* sp.	Tateyama, Japan (ND)	*Streptomyces javensis*	Actinobacteria	Unidentified	DOI (11 mm)	*C. albicans*	[113]
unidentified	Nagura Bay, Ishigaki, Japan (ND)	*Streptomyces albidoflavus*	Actinobacteria	Unidentified	DOI (16 mm)	*C. albicans*	[113]
unidentified	Nagura Bay, Ishigaki, Japan (ND)	*Streptomyces variabilis*	Actinobacteria	Unidentified	DOI (19 mm)	*C. albicans*	[113]
unidentified	Nagura Bay, Ishigaki, Japan (ND)	*Streptomyces luteosporeus*	Actinobacteria	Unidentified	DOI (24 mm)	*C. albicans*	[113]
*Spheciospongia vagabunda*	Rovinj, Croatia (3–20 m)	*Actinokineospora* sp. EG49	Actinobacteria	Unidentified	DOI (12 mm)	*C. albicans*	[82]
*Dysidea tupha*	Rovinj, Croatia (3–20 m)	*Streptomyces* sp. RV15	Actinobacteria	Unidentified	DOI (4–6 mm)	*C. albicans*	[82]
*Sigmadocia fibulatus*	Hare Island, India (5-10 m)	*Bacillus* sp. SC3	Firmicutes	Unidentified	DOI (15 mm)	*C. albicans*	[96]
*Sigmadocia fibulatus*	Hare Island, India (5-10 m)	*Pseudomonas* sp. SC11	Proteobacteria	Unidentified	DOI (7 mm)	*C. albicans*	[96]
*Echinodictyum* sp.	Hare Island, India (5-10 m)	*Idiomarina baltica* SA7	Proteobacteria	Unidentified	DOI (10 mm)	*C. albicans*	[96]
*Spongia* sp.	Hare Island, India (5-10 m)	*Staphylococcus equorum* SB11	Firmicutes	Unidentified	DOI (10 mm)	*C. albicans*	[96]
*Aplysina aerophoba*	Banyuls-sur-Mer, France (5–15 m)	*Bacillus subtilis* A184	Firmicutes	Surfactin, iturin, and fengycin	ND	*C. albicans*	[68]
*Aplysina aerophoba*	Banyuls-sur-Mer, France (5–15 m)	*Bacillus subtilis* A190	Firmicutes	Surfactin	ND	*C. albicans*	[68]
*Aplysina aerophoba*	Banyuls-sur-Mer, France (5–15 m)	*Bacillus subtilis* A202	Firmicutes	Iturin	ND	*C. albicans*	[68]
*Leucosolenia* sp.	Lough Hyne, Co. Cork, Ireland (15 m)	*Vibrio* sp. SC-C1-5	Proteobacteria	Unidentified	ND	*C. albicans*	[83]
*Leucosolenia* sp.	Lough Hyne, Co. Cork, Ireland (15 m)	*Vibrio* sp. BSw21697	Proteobacteria	Unidentified	ND	*C. albicans*	[83]
*Leucosolenia* sp.	Lough Hyne, Co. Cork, Ireland (15 m)	*Vibrio splendidus* LGP32	Proteobacteria	Unidentified	ND	*C. albicans*	[83]
*Leucosolenia* sp.	Lough Hyne, Co. Cork, Ireland (15 m)	*Bacillus amyloliquefaciens*	Proteobacteria	Unidentified	ND	*C. albicans*	[83]
*Leucosolenia* sp.	Lough Hyne, Co. Cork, Ireland (15 m)	*Vibrio* sp. SC-C1-5	Proteobacteria	Unidentified	ND	*Candida glabrata*	[83]
*Leucosolenia* sp.	Lough Hyne, Co. Cork, Ireland (15 m)	*Vibrio* sp. BSw21697	Proteobacteria	Unidentified	ND	*C. glabrata*	[83]
*Leucosolenia* sp.	Lough Hyne, Co. Cork, Ireland (15 m)	*Vibrio splendidus* LGP32	Proteobacteria	Unidentified	ND	*C. glabrata*	[83]
*Leucosolenia* sp.	Lough Hyne, Co. Cork, Ireland (15 m)	*Bacillus amyloliquefaciens*	Firmicutes	Unidentified	ND	*C. glabrata*	[83]
*Leucosolenia* sp.	Lough Hyne, Co. Cork, Ireland (15 m)	*Pseudoalteromonas* sp. A2B10	Proteobacteria	Unidentified	ND	*C. glabrata*	[83]
*Leucosolenia* sp.	Lough Hyne, Co. Cork, Ireland (15 m)	*Pseudoalteromonas* sp.* K2B-2*	Proteobacteria	Unidentified	ND	*C. glabrata*	[83]
*Leucosolenia* sp.	Lough Hyne, Co. Cork, Ireland (15 m)	*Pseudoalteromonas* sp.* LJ1*	Proteobacteria	Unidentified	ND	*C. glabrata*	[83]
*Leucosolenia* sp.	Lough Hyne, Co. Cork, Ireland (15 m)	*Pseudoalteromonas* sp.* S3178*	Proteobacteria	Unidentified	ND	*C. glabrata*	[83]
*Aplysina fistularis*	Sharm El-Sheikh, Egypt (ND)	*Streptomyces* sp. Hedaya48	Actinobacteria	Saadamycin	MIC (5 µg/mL*)*	*Trichophyton rubrum*	[107]
*Aplysina fistularis*	Sharm El-Sheikh, Egypt (ND)	*Streptomyces* sp. Hedaya48	Actinobacteria	5,7-Dimethoxy-4-*p*-methoxylphenylcoumarin	MIC (7.5 µg/mL*)*	*T. rubrum*	[107]
*Aplysina fistularis*	Sharm El-Sheikh, Egypt (ND)	*Streptomyces* sp. Hedaya48	Actinobacteria	Saadamycin	MIC (1.5 µg/mL)	*Trichophyton mentagrophytes*	[107]
*Aplysina fistularis*	Sharm El-Sheikh, Egypt (ND)	*Streptomyces* sp. Hedaya48	Actinobacteria	5,7-Dimethoxy-4-*p*-methoxylphenylcoumarin	MIC (90 µg/mL),	*T. mentagrophytes*	[107]
*Aplysina fistularis*	Sharm El-Sheikh, Egypt (ND)	*Streptomyces* sp. Hedaya48	Actinobacteria	Saadamycin	MIC (1.25 µg/mL)	*Microsporum gypseum*	[107]
*Aplysina fistularis*	Sharm El-Sheikh, Egypt (ND)	*Streptomyces* sp. Hedaya48	Actinobacteria	5,7-Dimethoxy-4-*p*-methoxylphenylcoumarin	MIC (100 µg/mL)	*M. gypseum*	[107]
*Aplysina fistularis*	Sharm El-Sheikh, Egypt (ND)	*Streptomyces* sp. Hedaya48	Actinobacteria	Saadamycin	MIC (1.0 µg/mL)	*Epidermophyton floccosum*	[107]
*Aplysina fistularis*	Sharm El-Sheikh, Egypt (ND)	*Streptomyces* sp. Hedaya48	Actinobacteria	5,7-Dimethoxy-4-*p*-methoxylphenylcoumarin	MIC (50 µg/mL)	*E. floccosum*	[107]
*Aplysina fistularis*	Sharm El-Sheikh, Egypt (ND)	*Streptomyces* sp. Hedaya48	Actinobacteria	Saadamycin	MIC (1.2 µg/mL)	*Fusarium oxysporum*	[107]
*Aplysina fistularis*	Sharm El-Sheikh, Egypt (ND)	*Streptomyces* sp. Hedaya48	Actinobacteria	5,7-Dimethoxy-4-*p*-methoxylphenylcoumarin	MIC (22 µg/mL)	*F. oxysporum*	[107]
*Aplysina fistularis*	Sharm El-Sheikh, Egypt (ND)	*Streptomyces* sp. Hedaya48	Actinobacteria	Saadamycin	MIC (5.16 µg/mL)	*Cryptococcus humicolus*	[107]
*Aplysina fistularis*	Sharm El-Sheikh, Egypt (ND)	*Streptomyces* sp. Hedaya48	Actinobacteria	5,7-Dimethoxy-4-*p*-methoxylphenylcoumarin	MIC (10 µg/mL)	*C. humicolus*	[107]
*Halichondria japonica*	Iriomote island, Japan (ND)	*Phoma* sp. Q60596	Ascomycota	YM-202204	IC_80_ (1.56 µg/mL)	*Cryptococcus neoformans*	[108]
*Aplysina fistularis*	Sharm El-Sheikh, Egypt (ND)	*Streptomyces* sp. Hedaya48	Actinobacteria	Saadamycin	MIC (1.6 µg/mL)	*Aspergillus fumigatus*	[107]
*Aplysina fistularis*	Sharm El-Sheikh, Egypt (ND)	*Streptomyces* sp. Hedaya48	Actinobacteria	5,7-Dimethoxy-4-*p*-methoxylphenyl-coumarin	MIC (10 µg/mL)	*A. fumigatus*	[107]
*Halichondria japonica*	Iriomote island, Japan (ND)	*Phoma* sp. Q60596	Ascomycota	YM-202204	IC_80_ (12.5 µg/mL)	*A. fumigatus*	[108]
*Leucosolenia* sp.	Lough Hyne, Co. Cork, Ireland (15 m)	*Staphylococcus saprophyticus*	Firmicutes	Unidentified	ND	*A. fumigatus*	[83]
*Leucosolenia* sp.	Lough Hyne, Co. Cork, Ireland (15 m)	*Staphylococcus* sp. HJB003	Firmicutes	Unidentified	ND	*A. fumigatus*	[83]
*Leucosolenia* sp.	Lough Hyne, Co. Cork, Ireland (15 m)	*Vibrio litoralis* MANO22P	Proteobacteria	Unidentified	ND	*A. fumigatus*	[83]
*Leucosolenia* sp.	Lough Hyne, Co. Cork, Ireland (15 m)	*Vibrio* sp. SC-C1-5	Proteobacteria	Unidentified	ND	*A. fumigatus*	[83]
*Leucosolenia* sp.	Lough Hyne, Co. Cork, Ireland (15 m)	*Vibrio* sp. BSw21697	Proteobacteria	Unidentified	ND	*A. fumigatus*	[83]
*Leucosolenia* sp.	Lough Hyne, Co. Cork, Ireland (15 m)	*Vibrio splendidus* LGP32	Proteobacteria	Unidentified	ND	*A. fumigatus*	[83]
*Leucosolenia* sp.	Lough Hyne, Co. Cork, Ireland (15 m)	*Bacillus amyloliquefaciens*	Firmicutes	Unidentified	ND	*A. fumigatus*	[83]
*Aplysina fistularis*	Sharm El-Sheikh, Egypt (ND)	*Streptomyces* sp. Hedaya48	Actinobacteria	Saadamycin	MIC (1.0 µg/mL)	*Aspergillus niger*	[107]
*Aplysina fistularis*	Sharm El-Sheikh, Egypt (ND)	*Streptomyces* sp. Hedaya48	Actinobacteria	5,7-Dimethoxy-4-*p*-methoxylphenylcoumarin	MIC (20 µg/mL)	*A. niger*	[107]
*Halichondria* sp.	West Coast of India (10 m)	*Bacillus* sp. SAB1	Firmicutes	3-Phenylpropionic acid	DOI (1–3 mm) at 50 µg/disc	*A. niger*	[69]
*Halichondria* sp.	West Coast of India (10 m)	*Bacillus* sp. SAB1	Firmicutes	4,4′-Oxybis(3-phenylpropionic acid)	DOI (4–6 mm) at 50 µg/disc	*A. niger*	[69]
*Halichondria* sp.	West Coast of India (10 m)	*Bacillus* sp. SAB1	Firmicutes	3-Phenylpropionic acid	DOI (4–6 mm) at 50 µg/disc	*Rhodotorula* sp.	[69]
*Halichondria* sp.	West Coast of India (10 m)	*Bacillus* sp. SAB1	Firmicutes	4,4′-Oxybis(3-phenylpropionic acid)	DOI (7–10 mm) at 50 µg/disc	*Rhodotorula* sp.	[69]
*Halichondria japonica*	Iriomote island, Japan (ND)	*Phoma* sp. Q60596	Ascomycota	YM-202204	IC_80_ (1.56 µg/mL)	*Saccharomyces cerevisiae*	[108]
*Hymeniacidon perleve*	Nanji island, China (ND)	*Pseudoalteromonas piscicida NJ6-3-1*	Proteobacteria	Norharman (a beta-carboline alkaloid)	DOI (3–5 mm)	*S. cerevisiae*	[59]
*Hymeniacidon perleve*	Nanji island, China (ND)	*Bacillus megaterium NJ6-3-2*	Firmicutes	Unidentified	DOI (3–5 mm)	*S. cerevisiae*	[59]
*Leucosolenia* sp.	Lough Hyne, Co. Cork, Ireland (15 m)	*Vibrio litoralis* MANO22P	Proteobacteria	Unidentified	ND	*S. cerevisiae*	[83]
*Leucosolenia* sp.	Lough Hyne, Co. Cork, Ireland (15 m)	*Vibrio* sp. SC-C1-5	Proteobacteria	Unidentified	ND	*S. cerevisiae*	[83]
*Leucosolenia* sp.	Lough Hyne, Co. Cork, Ireland (15 m)	*Vibrio* sp. BSw21697	Proteobacteria	Unidentified	ND	*S. cerevisiae*	[83]
*Leucosolenia* sp.	Lough Hyne, Co. Cork, Ireland (15 m)	*Vibrio splendidus* LGP32	Proteobacteria	Unidentified	ND	*S. cerevisiae*	[83]
*Leucosolenia* sp.	Lough Hyne, Co. Cork, Ireland (15 m)	*Bacillus amyloliquefaciens*	Firmicutes	Unidentified	ND	*S. cerevisiae*	[83]
*Psammocinia* sp.	Sdot-Yam, Israel (ND)	*Aspergillus insuetus*	Ascomycota	Insuetolides A	MIC (60.09 µg/mL)	*Neurospora crassa*	[114]
*Psammocinia* sp.	Sdot-Yam, Israel (ND)	*Aspergillus insuetus*	Ascomycota	Strobilactone A	MIC (69.97 µg/mL)	*N. crassa*	[114]
*Psammocinia* sp.	Sdot-Yam, Israel (ND)	*Aspergillus insuetus*	Ascomycota	(*E*,*E*)-6-(60,70-Dihydroxy-20,40-octadienoyl)-strobilactone A	MIC (71.79 µg/mL)	*N. crassa*	[114]
*Myxilla incrustans*	The Caribbean Island of Dominica (ND)	*Microsphaeropsis* sp.	Ascomycota	Microsphaeropsisin	ND	*Eurotium repens*	[115]
*Myxilla incrustans*	The Caribbean Island of Dominica (ND)	*Microsphaeropsis* sp.	Ascomycota	(*R*)-Mellein	ND	*E. repens*	[115]
*Myxilla incrustans*	The Caribbean Island of Dominica (ND)	*Microsphaeropsis* sp.	Ascomycota	(3*R*,4*R*)-Hydroxymellein	ND	*E. repens*	[115]
*Myxilla incrustans*	The Caribbean Island of Dominica (ND)	*Microsphaeropsis* sp.	Ascomycota	4,8-Dihydroxy-3,4-dihydro-2*H*-naphthalen-1-one	ND	*E. repens*	[115]
*Ectyoplasia ferox*	The Caribbean Island of Dominica (ND)	*Coniothyrium* sp.	Ascomycota	(3*R*)-6-Methoxymellein	ND	*E. repens*	[115]
*Ectyoplasia ferox*	The Caribbean Island of Dominica (ND)	*Coniothyrium* sp.	Ascomycota	(3*R*)-6-Methoxy-7-chloromellein	ND	*E. repens*	[115]
*Ectyoplasia ferox*	The Caribbean Island of Dominica (ND)	*Coniothyrium* sp.	Ascomycota	(*p*-Hydroxyphenyl) ethanol	ND	*E. repens*	[115]
*Ectyoplasia ferox*	The Caribbean Island of Dominica (ND)	*Coniothyrium* sp.	Ascomycota	Phenylethanol	ND	*E. repens*	[115]
*Myxilla incrustans*	The Caribbean Island of Dominica (ND)	*Microsphaeropsis* sp.	Ascomycota	Microsphaeropsisin	ND	*Ustilago violacea*	[115]
*Myxilla incrustans*	The Caribbean Island of Dominica (ND)	*Microsphaeropsis* sp.	Ascomycota	(*R*)-Mellein	ND	*U. violacea*	[115]
*Myxilla incrustans*	The Caribbean Island of Dominica (ND)	*Microsphaeropsis* sp.	Ascomycota	(3*R*,4*R*)-Hydroxymellein	ND	*U. violacea*	[115]
*Myxilla incrustans*	The Caribbean Island of Dominica (ND)	*Microsphaeropsis* sp.	Ascomycota	4,8-Dihydroxy-3,4-dihydro-2*H*-naphthalen-1-one	ND	*U. violacea*	[115]
*Ectyoplasia ferox*	The Caribbean Island of Dominica (ND)	*Coniothyrium* sp.	Ascomycota	(3*R*)-6-Methoxymellein	ND	*U. violacea*	[115]
*Ectyoplasia ferox*	The Caribbean Island of Dominica (ND)	*Coniothyrium* sp.	Ascomycota	(3*R*)-6-Methoxy-7-chloromellein	ND	*U. violacea*	[115]
*Ectyoplasia ferox*	The Caribbean Island of Dominica (ND)	*Coniothyrium* sp.	Ascomycota	(*p*-Hydroxyphenyl) ethanol	ND	*U. violacea*	[115]
*Ectyoplasia ferox*	The Caribbean Island of Dominica (ND)	*Coniothyrium* sp.	Ascomycota	Phenylethanol	ND	*U. violacea*	[115]
*Ectyoplasia ferox*	The Caribbean Island of Dominica (ND)	*Coniothyrium* sp.	Ascomycota	(3*S*)-(3’,5’-Dihydroxyphenyl)butan-2-one	ND	*U. violacea*	[115]
*Ectyoplasia ferox*	The Caribbean Island of Dominica (ND)	*Coniothyrium* sp.	Ascomycota	(3*S*)-(3’,5’-Dihydroxyphenyl)butan-2-one	ND	*Mycotypha microspora*	[115]

Table 3 is organised according to the target fungi. IC_50_: half maximum inhibitory concentration; IC_80_: 80% inhibitory concentration; MIC: minimum inhibitory concentration; DOI: diameter of inhibition; ND: not determined.

**Table 4:** Bioactive compounds with antiprotozoal activity from sponge-associated microbes.

Sponge	Origin (Depth)	Microorganism	Phylum	Compound	Property	Target	References
*Homophymia* sp.	Touho, New Caledonia (ND)	*Pseudomonas* sp. 1531-E7	Proteobacteria	2-Undecyl-4-quinolone	IC_50_ (1 μg/mL)	*Plasmodium falciparum*	[25]
*Acanthostrongylophora ingens*	Manado, Indonesia (ND)	*Micromonospora* sp. M42	Actinobacteria	Manzamine A	IC_50_ (0.0045 µg/mL)	*P. falciparum*	[124,125,126,127]
*Hyattella intestinalis*	Palk strait, Tamil Nadu, India (ND)	unidentified bacterial isolate THB20	Unidentified	Unidentified	IC_50_ (41.88 µg/mL)	*P. falciparum*	[131]
*Stylissa carteri*	Palk strait, Tamil Nadu, India (ND)	unidentified bacterial isolate THB17	Unidentified	Unidentified	IC_50_ (20.56 µg/mL)	*P. falciparum*	[132]
*Clathria indica*	Palk strait, Tamil Nadu, India (ND)	unidentified bacterial isolate THB23	Unidentified	Unidentified	IC_50_ (28.80 µg/mL)	*P. falciparum*	[133]
*Clathria vulpina*	Palk strait, Tamil Nadu, India (ND)	unidentified bacterial isolate THB15	Unidentified	Unidentified	IC_50_ (20.73 µg/mL)	*P. faciparum*	[134]
*Haliclona grant*	Palk strait, Tamil Nadu, India (ND)	unidentified bacterial isolate THB14	Unidentified	Unidentified	IC_50_ (11.98 µg/mL*)*	*P. faciparum*	[135]
*Acanthostrongylophora ingens*	Manado, Indonesia (ND)	*Micromonospora* sp. M42	Actinobacteria	Manzamine A	*In vivo* inhibition (90%) at concentration of 0.008 µg/mL	*Plasmodium berghei*	[116,125,126,127]
*Aplysina aerophoba*	Rovinj, Croatia (3–20 m)	*Micromonospora* sp. RV115	Actinobacteria	Diazepinomicin	IC_50_ (6.29 µg/mL)	*Trypanosoma brucei*	[136]
*Spheciospongia vagabunda*	Rovinj, Croatia (3–20 m)	*Actinokineospora* sp. EG49	Actinobacteria	Unidentified	Percentage of growth inhibition (48%)	*T. brucei*	[82]
unidentified	Rovinj, Croatia (3–20 m)	*Brevibacterium* sp. EG10	Actinobacteria	Unidentified	Percentage of growth inhibition growth inhibition (30%)	*T. brucei*	[82]
unidentified	Rovinj, Croatia (3–20 m)	*Gordonia* sp. EG50	Actinobacteria	Unidentified	Percentage of growth inhibition growth inhibition (28%)	*T. brucei*	[82]
*Dysidea tupha*	Rovinj, Croatia (3–20 m)	*Kocuria* sp. RV89	Actinobacteria	Unidentified	Percentage of growth inhibition growth inhibition (19%)	*T. brucei*	[82]
*Dysidea avara*	Mediterranean sea (ND)	*Nocardiopsis* sp.* RV163*	Actinobacteria	1,6-Dihydroxyphenazine (produced from co-culture)	IC_50_ (4.03 µg/mL)	*T*.* brucei*	[98]
*Spheciospongia vagabunda*	Red Sea (ND)	*Actinokinespora* sp. EG49	Actinobacteria	Actinosporin A	IC_50_ (19.19 µg/mL)	*T. brucei brucei*	[137]
*Aplysina* *polypoides*	Rovinj, Croatia (3–20 m)	*Streptomyces* sp. 34	Actinobacteria	Valinomycin	IC_50_ (0.0036 µg/mL)	*T. brucei brucei*	[128]
*Axinella aerophoba*	Rovinj, Croatia (3–20 m)	*Streptomyces* sp. 22	Actinobacteria	Valinomycin	IC_50_ (0.0036 µg/mL)	*T. brucei brucei*	[128]
*Tedania* sp*.*	Rovinj, Croatia (3–20 m)	*Streptomyces* sp. 11	Actinobacteria	Staurosporine	IC_50_ (0.0051 µg/mL)	*T. brucei brucei*	[128]
*Tethya* sp*.*	Rovinj, Croatia (3–20 m)	*Streptomyces* sp. T03	Actinobacteria	Butenolide	IC_50_ (7.92 µg/mL)	*T. brucei brucei*	[128]
*Petrosia ficiformis*	Milos, Greece (ND)	*Streptomyces* sp. SBT344	Actinobacteria	Unidentified	IC_50_ (<10 µg/mL)	*T. brucei brucei*	[138]
*Sarcotragus foetidus*	Milos, Greece (ND)	*Modestobacter* sp. SBT363	Actinobacteria	Unidentified	IC_50_ (<10 µg/mL)	*T. brucei brucei*	[138]
*Sarcotragus foetidus*	Milos, Greece (ND)	*Nonomuraea* sp. SBT364	Actinobacteria	Unidentified	IC_50_ (<10 µg/mL)	*T. brucei brucei*	[138]
*Phorbas tenacior*	Crete, Greece (ND)	*Micromonospora* sp. SBT687	Actinobacteria	Unidentified	IC_50_ (14.87 µg/mL)	*T. brucei brucei*	[138]
*Petrosia ficiformis*	Milos, Greece (ND)	*Streptomyces* sp. SBT348	Actinobacteria	Unidentified	IC_50_ (16.52 µg/mL)	*T. brucei brucei*	[138]
*Ircinia variabilis*	Milos, Greece (ND)	*Geodermatophilus* sp. SBT381	Actinobacteria	Unidentified	IC_50_ (18.60 µg/mL)	*T. brucei brucei*	[138]
*Spirastrella cunctatrix*	Milos, Greece (ND)	*Modestobacter* sp. SBT362	Actinobacteria	Unidentified	IC_50_ (19.34 µg/mL)	*T. brucei brucei*	[138]
*Spirastrella cunctatrix*	Milos, Greece (ND)	*Rhodococcus* sp. SBT367	Actinobacteria	Unidentified	IC_50_ (19.97 µg/mL)	*T. brucei brucei*	[138]
*Axinella polypoides*	Banyuls-sur-Mer, France (ND)	*Streptomyces axinellae* Pol001T	Actinobacteria	Tetromycin 1	IC_50_ (26.02 µg/mL)	*T. brucei brucei*	[139]
*Axinella polypoides*	Banyuls-sur-Mer, France (ND)	*Streptomyces axinellae* Pol001T	Actinobacteria	Tetromycin 2	IC_50_ (40.35 µg/mL)	*T. brucei brucei*	[139]
*Axinella polypoides*	Banyuls-sur-Mer, France (ND)	*Streptomyces axinellae* Pol001T	Actinobacteria	Tetromycin 3	IC_50_ (23.18 µg/mL)	*T. brucei brucei*	[139]
*Axinella polypoides*	Banyuls-sur-Mer, France (ND)	*Streptomyces axinellae* Pol001T	Actinobacteria	Tetromycin 4	IC_50_ (32.17 µg/mL)	*T. brucei brucei*	[139]
*Axinella polypoides*	Banyuls-sur-Mer, France (ND)	*Streptomyces axinellae* Pol001T	Actinobacteria	Tetromycin B	IC_50_ (17.20 µg/mL)	*T. brucei brucei*	[139]
*Aplysina polypoides*	Rovinj, Croatia (3–20 m)	*Streptomyces* sp. 34	Actinobacteria	Valinomycin	IC_50_ (<0.12 µg/mL)	*Leishmania major*	[128]
*Axinella aerophoba*	Rovinj, Croatia (3–20 m)	*Streptomyces* sp. 22	Actinobacteria	Valinomycin	IC_50_ (<0.12 µg/mL)	*L. major*	[128]
*Tedania* sp.	Rovinj, Croatia (3–20 m)	*Streptomyces* sp. 11	Actinobacteria	Staurosporine	IC_50_ (1.24 µg/mL)	*L. major*	[128]
*Axinella polypoides*	Banyuls-sur-Mer, France (ND)	*Streptomyces axinellae* Pol001T	Actinobacteria	Tetromycin 3	IC_50_ (31.72 µg/mL)	*L. major*	[139]
*Spheciospongia vagabunda*	Rovinj, Croatia (3–20 m)	*Actinokineospora* sp. EG49	Actinobacteria	Unidentified	growth inhibition (48%)	*L. major*	[82]
unidentified	Rovinj, Croatia (3–20 m)	*Gordonia* sp. EG50	Actinobacteria	Unidentified	growth inhibition (28%)	*L. major*	[82]
*Axinella corrugata*	The Arvoredo Biological Marine Reserve, Brazil (ND)	*Hypocrea lixii* F02	Ascomycota	Unidentified	MIC (250 µg/mL)	*Trichomonas vaginalis* ATCC 30236	[129]
*Axinella corrugata*	The Arvoredo Biological Marine Reserve, Brazil (ND)	*Hypocrea lixii* F02	Ascomycota	Unidentified	MIC (250 µg/mL)	*T. vaginalis* fresh isolate	[129]
*Axinella corrugata*	The Arvoredo Biological Marine Reserve, Brazil (ND)	*Hypocrea lixii* F02	Ascomycota	Unidentified	MIC (250 µg/mL)	*T. vaginalis* metronidazole-resistant LACM2	[129]
*Stoeba* sp.	The Arvoredo Biological Marine Reserve, Brazil (ND)	*Penicillium citrinum* F40	Ascomycota	Unidentified	MIC (250 µg/mL)	*T. vaginalis* ATCC 30236	[129]
*Stoeba* sp.	The Arvoredo Biological Marine Reserve, Brazil (ND)	*Penicillium citrinum* F40	Ascomycota	Unidentified	MIC (250 µg/mL)	*T. vaginalis* fresh isolate	[129]
*Stoeba* sp.	The Arvoredo Biological Marine Reserve, Brazil (ND)	*Penicillium citrinum* F40	Ascomycota	Unidentified	MIC (250 µg/mL)	*T.vaginalis* metronidazole-resistant LACM2	[129]

Table 4 is organised according to the target protozoa. IC_50_: half maximum inhibitory concentrations; MIC: minimum inhibitory concentration; ND: not determined.
